# Synthesis of Orthogonally Protected Labionin

**DOI:** 10.1021/acs.joc.0c02922

**Published:** 2021-02-18

**Authors:** Eliana Lo Presti, Alessandro Volonterio, Monica Sani

**Affiliations:** † National Research Council, Institute of Chemical Sciences and Technologies “Giulio Natta” (SCITEC), via Mario Bianco 9, 20131 Milan, Italy; ‡ Department of Chemistry, Materials, and Chemical Engineering “Giulio Natta”, Politecnico di Milano, via Mancinelli 7, 20141 Milan, Italy

## Abstract

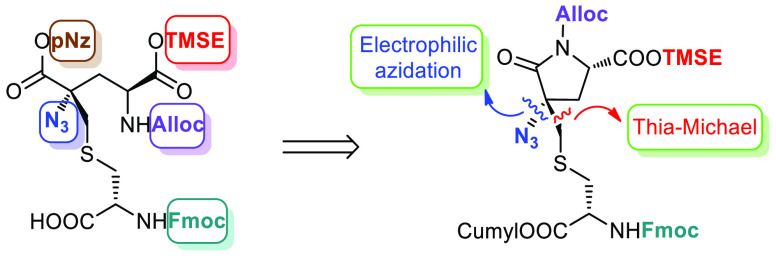

We report the first synthesis of the complex amino acid labionin in a fully orthogonally protected and stereopure form. The structure—which incorporates five orthogonal protecting groups and three stereogenic centers—was assembled using two key synthetic steps: (1) a thia-Michael addition for installing the thioether bridge; (2) an electrophilic azidation for creating the central quaternary α-amino acid carbon in a stereochemically pure form. This work is expected to enable the solid phase synthesis of both natural and synthetic analogues labyrinthopeptins.

Labyrinthopeptins constitute a class of ribosomally synthesized peptides discovered in 2010, that belong to the family of type III lantibiotics.^1^ These lantipeptides incorporate the distinctive post-translationally modified amino acid labionin (Lab **1**; Figure 1), which is a triamino triacid featuring an unusual central quaternary carbon. Structurally, Lab **1** can be described as a derivative of meso-lanthionine (Lan) (Figure 1) where the (*S*)-α-carbon is connected to a further amino acid through a methylene bridge.

**Figure 1:**
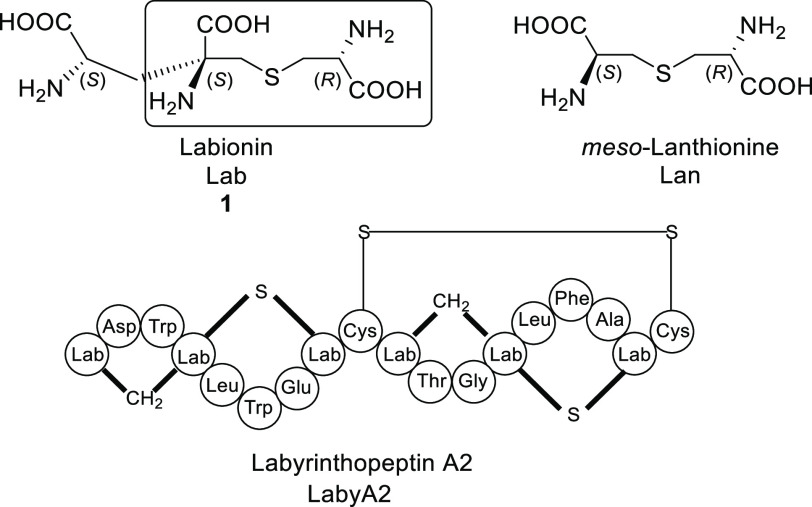
Structure of labionin, meso-lanthionine, and labyrinthopeptin A2.

Unlike lanthionine-containing peptides, labyrinthopeptins possess weak antibacterial activity. On the other hand, they display an antiviral activity in the low micromolar range especially against hRSV.^2^ More interestingly, labyrinthopeptin A2 (LabyA2) (Figure 1) was found effective in animal pain models, showing excellent antiallodynic activity, although the actual mechanism of action is still unknown.^1^ Since these compounds can be considered as potential novel drugs for the treatment of neuropathic pain, there is a strong need to perform structure–activity relationship (SAR) studies in order to identify key structural features important for their bioactivity.

In this context, solid phase peptide synthesis (SPPS) represents an attractive tool to prepare combinatorial libraries of synthetic analogues, which cannot be obtained by biosynthetic manipulation. Nevertheless, a SPPS approach to these lantipeptides is nontrivial because, involving the direct incorporation of labionin into the growing peptide chain, it requires the availability of opportunely protected and stereochemically pure labionin amino acid. Although the literature reported several reviews about the synthesis of lanthionine,^3^ a solution chemical route to labionin is still missing. As far as we know there is only a preliminary study toward this goal, published in 2001 by Süssmuth et al.^4^

From a synthetic point of view, labionin features two main challenges: (1) the presence of three stereogenic centers, one of which is quaternary, and (2) the chemodifferentiation for the three carboxylic acid and three amino functional groups, using orthogonal protective groups compatible with both solution and solid phase techniques.

Herein we report, for the first time, the synthesis of orthogonally protected Labionin **2** (Figure 2) in diastereomerically pure form, which can be considered an excellent building block for a future SPPS approach to labionin-contaning peptides. In fact, all selected protecting groups are orthogonal to each other as well as to the transient Fmoc and permanent Boc/tBu used in Fmoc SPPS. They can be chemoselectively removed as follows: (a) palladium-catalyzed transfer of the allyl unit to a scavenger for Alloc carbamate; (b) Staudinger reaction for the azido group; (c) fluoride mediated removal for TMSE ester; (d) reduction with SnCl_4_ in nearly neutral condition for pNz ester.^5^

**Figure 2:**
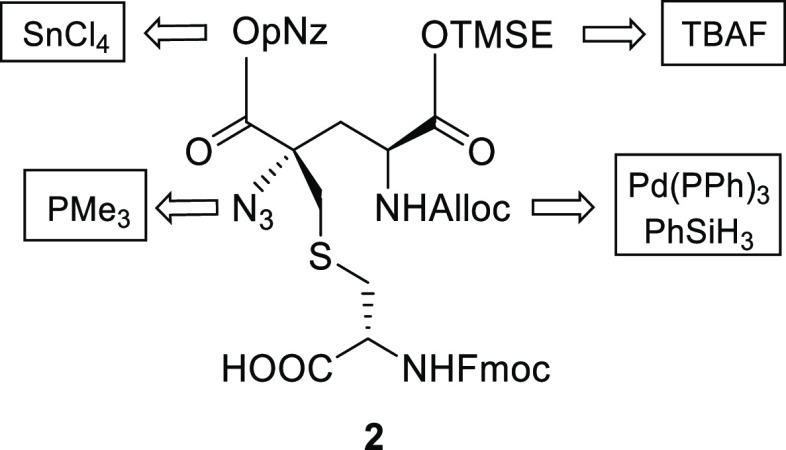
Fully orthogonal protected Labionin **2** and chemoselective removal conditions of each of five functional groups.

Our retrosynthetic analysis suggested that a thia-Michael addition followed by an electrophilic azidation could represent a viable entry to introduce the α,α-carbon stereocenter in a stereoselective way. The key intermediate α,β-unsaturated lactam **4** could, in turn, be obtained through a α-methylenation reaction performed on the opportunely protected l-pyroglutamic acid **5**, which already incorporates one of the stereocenters with the correct stereochemistry (Scheme 1)

**Scheme 1:**
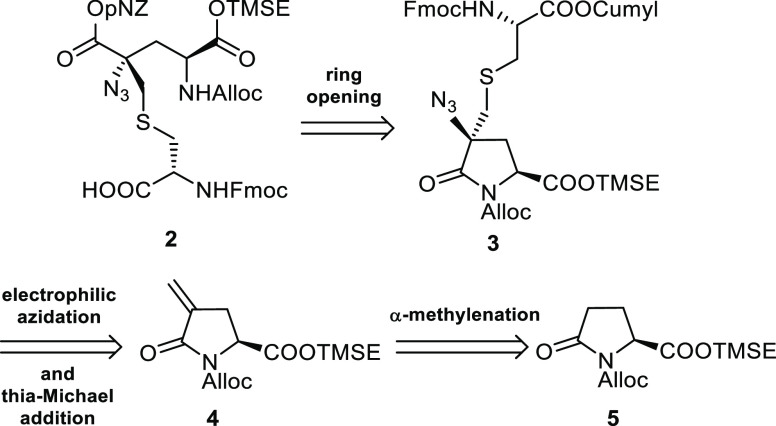
Retrosynthetic Analysis of Labionin **2**

Accordingly, (*S*)-pyroglutamic acid **6** was converted into 4-methylenic derivative **4** in 60% overall yield through five scalable synthetic steps (Scheme 2).

**Scheme 2:**
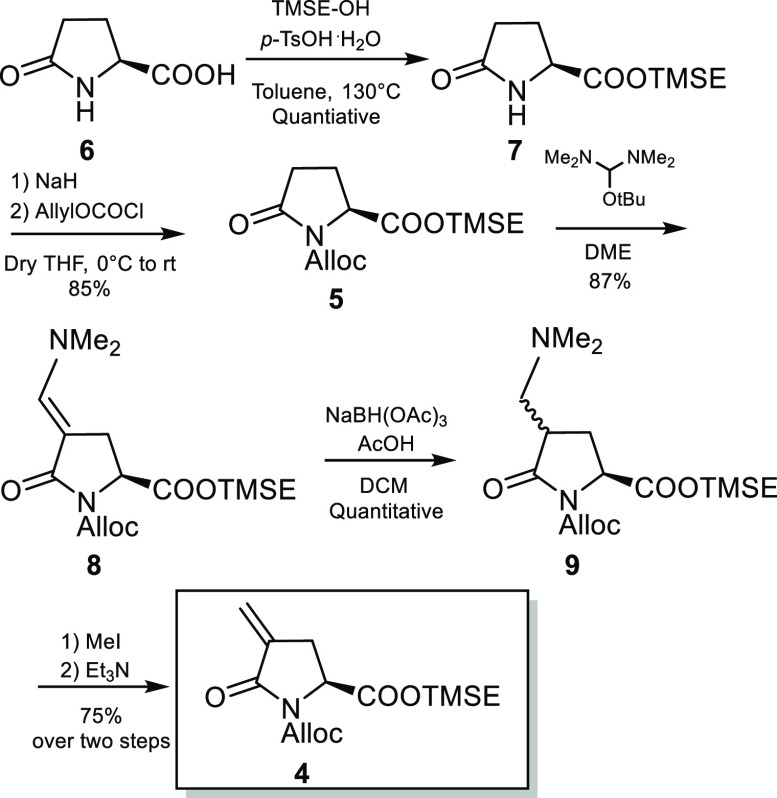
Synthesis of α,β-Unsaturated Lactam **4**

First, the carboxylic acid group and the lactamic nitrogen of **6** were respectively protected as trimethylsilyl ethyl ester^6^ and allyloxycarbonyl carbamate.^7^ N-protected-pyroglutamate **8** was then submitted to efficient α-methylenation by means of a two-steps sequence involving a C-4 enamination followed by a Hoffman’ s elimination. Enaminone **8** was obtained in high yields according to the previously reported procedure^8^ by reaction with *t*-BuOCH(NMe_2_)_2_ (Bredereck’s reagent) at 75 °C in 1,2-dimethoxyethane. Although Young et al. reported DIBAL as reagent for carrying out the enaminone reduction, we had to choose a more selective reducing agent in order to avoid side reactions due to the presence of the allyloxycarbonyl group. We were delighted to find that by using NaBH(OAc)_3_ in the presence of AcOH we were able to obtain in high yields a diastereoisomeric mixture of amines **9** that, in turn, were efficiently converted into the desired α-methylene lactam **4** by treatment with MeI followed by Et_3_N.^9^

We next turned our attention to the synthesis of the second fully protected chiral building block, the (*R*)-cysteine derivative **12**. Two additional masking groups had to be selected for the future chemoselective manipulation: a Fmoc group for the transient protection of the amino function and a cumyl ester for the carboxylic moiety. Cumyl ester was chosen because of its mild removal conditions (2% vol of TFA in DCM) which fulfills the requirement of orthogonality to the other protecting groups^6^ (Scheme 3).

**Scheme 3:**
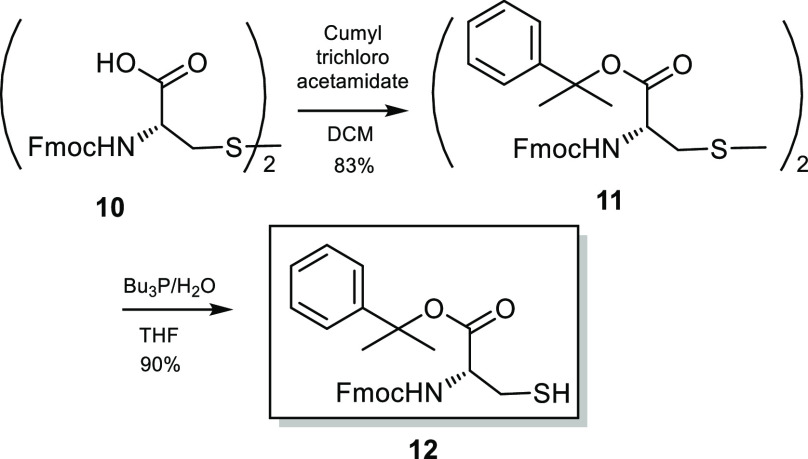
Synthesis of the Orthogonally Protected (*R*)-Cysteine **12**

Accordingly, orthogonally protected (*R*)-cysteine **12** was obtained in excellent yields, starting from commercially available bis-Fmoc-l-cystine. The procedure involved esterification of the carboxylic acid of **10** with cumyl trichloroacetamidate, synthesized as previously reported in the literature,^9^ followed by a selective cleavage of the disulfide bridge with Bu_3_P and water (Scheme 3). With the building blocks **4** and **12** in hand, we focused our attention on the two key steps of our strategy: the thioether bridge formation and the installation of the quaternary stereocenter. Et_3_N-catalyzed thia-Michael addition of cysteine **12** to α,β-unsaturated lactam **4** proved to be an excellent approach to install the thioether moiety. Indeed the reaction proceeded in high yield, affording straightforwardly a 3:1 mixture of diastereoisomers, which were easily separated by flash chromatography (FC) (Scheme 4).

**Scheme 4:**
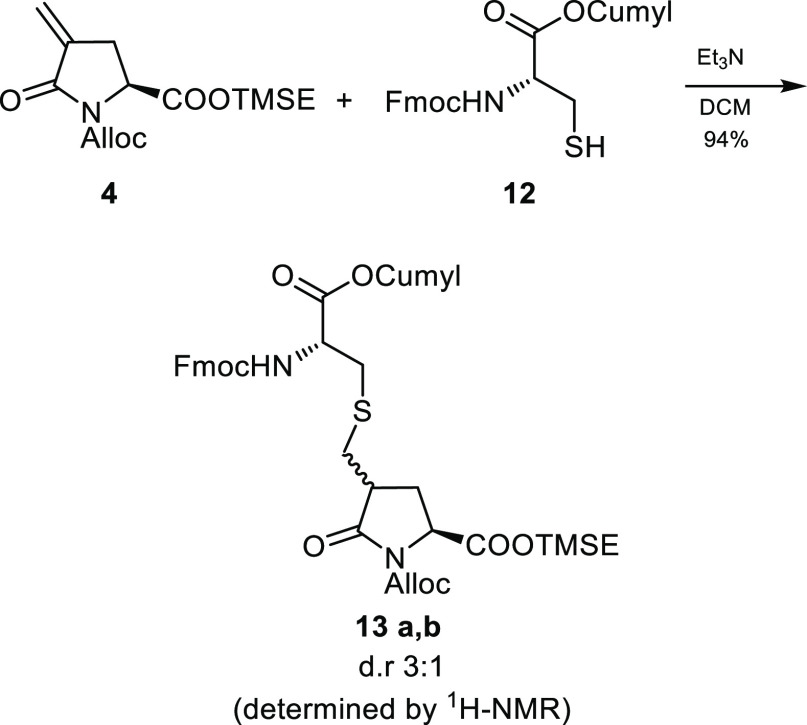
Thioether Bridge Creation by Base-Catalyzed Thia-Michael Addition

With compounds **13a** and **13b** in hand, we turned our attention on the construction of the α,α-disubstituted central carbon by mean of an electrophilic azidation.

Lactam enolates derived from N-urethane-protected pyroglutamates are known to undergo monofunctionalization in the position 4 without loss of optical purity and in a stereocontrolled manner.^10^ On the other hand, double substitutions are much less common and limited to double alkylations.^11^ Moreover, to the best of our knowledge, no examples of direct azide incorporation into pyroglutamates have been reported until now.

The reaction was performed following the protocol developed by Evans et al. for the electrophilic azidation of imide enolates.^12^ The mixture of diastereomers **13a**,**b** was treated with 2.2 equiv of LiHMDS (an extra equivalent of base is needed to scavenge the acidic amide proton) in order to generate the pyroglutamic Li-enolate, which was reacted with 2,4,6-triisopropylbenzenesulfonyl azide (trisyl azide). The reaction produced in good yield a 1.8:1 epimeric mixture of α,α-disubstituted pyroglutamates **3a** and **3b**, that can be easily separated by FC (Scheme 5).

**Scheme 5:**
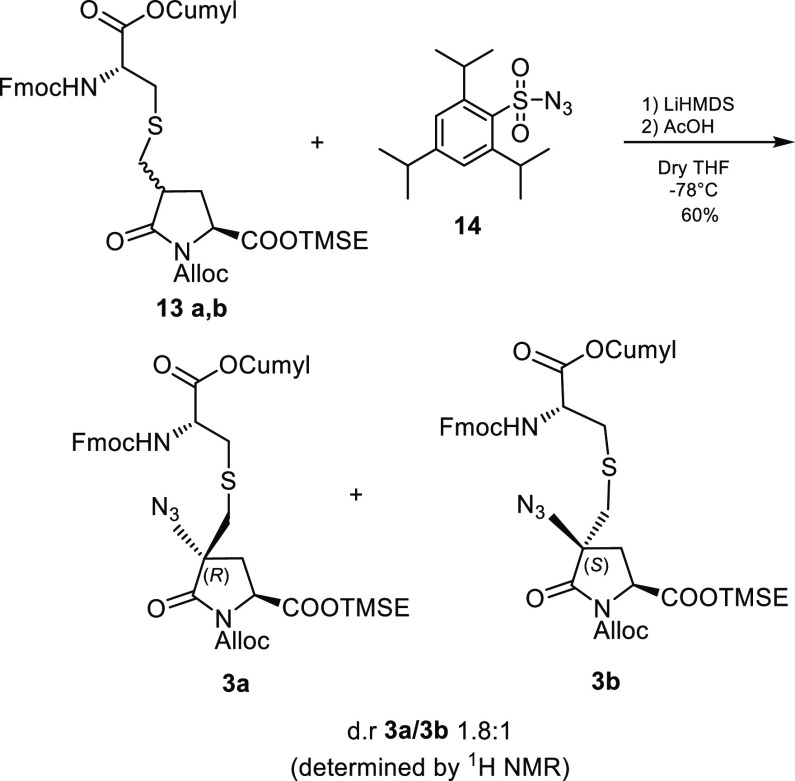
α,α-Disubstituted Central Carbon Installation by Direct Electrophilic Azidation

Assignment of the absolute stereochemistry of the newly quaternary stereocenter was performed by ^1^H NMR and 2D-NMR studies, allowing us to assess the configuration S to the minor epimer (see Supporting Information). This result is in line with that obtained by Ezquerra et al. in the double alkylation of N-Boc-pyroglutamate.^10a^ It is worth noting that the quaternary stereocenter in the natural labionin has configuration (S). However, the possibility to have both diastereoisomers in pure form is favorable for SARs purposes. With the aim of improving either the yield or/and the diastereoselection of the process, we studied in detail the influence of the reaction conditions such as metal coordination, temperature, solvent, and the stereochemistry of the starting materials. Unfortunately all attempts failed to reach our goal. Indeed when KHMDS or HMPA were used to generate a less coordinated enolate, the reaction did not afford the desired compounds, leading to the degradation of the starting material, suggesting that the pyroglutamic enolate requires a coordinating metal in order to be stable and reactive. Performing the reaction on stereochemically pure **13a** and **13b**, we did not observe remarkable improvement in terms of *S:R* ratio and yield. Lastly, similar results were obtained when a lower dielectric constant solvent as toluene was used.

Fully protected Labionin **2** was then successfully obtained by a three-step process. Selective ring opening of the lactam **3b** was achieved by basic hydrolysis with LiOH in THF/H_2_O 5:2 at 0 °C, leading to the derivative **15b** in high yield. The free carboxylic function of **15b** was subsequently protected as 4-nitrobenzyl ester in dry DMF with 4-nitrobenzyl bromide and KI in a 74% yield. Final cleavage of cumyl ester with 2% TFA in DCM led to target compound Lab **2** in almost quantitative yield (Scheme 6). The same synthetic pathway was also performed on the 4R epimer (see Supporting Information).

**Scheme 6:**
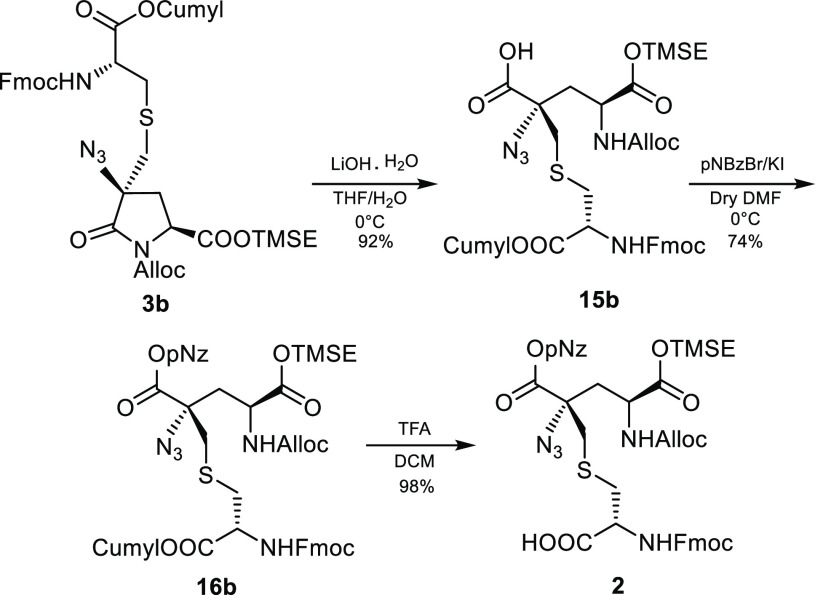
Completion of Labionin **2** Synthesis

In summary, we have developed for the first time a synthesis of orthogonally protected labionin in stereochemically pure form, overcoming the challenge of selecting six different protective groups suitable for SPPS and stable to the reaction conditions of our synthetic plan.

Labionin **2** can be considered an excellent building block for a future solid supported chemical synthesis of both natural labyrinthopeptins and non-native analogues that cannot be obtained by biosynthetic manipulation. Moreover this route should allow the access to all the possible labionin stereoisomers for stereochemistry–activity relationship studies in order to reveal the importance of the cross-links (the thioether and the methylenic bridges) stereochemistry to the biological activity. Future work toward the solid phase synthesis of labyrinthopeptins as well as attempts to obtain in a stereospecific fashion the amino acid labionin will be presented in due course.

## Experimental Section

### General Methods

Commercially available reagents, purchased from Sigma-Aldrich and Fluorochem, were employed without any further purification. Anhydrous THF was purchased by Sigma-Aldrich. Thin layer chromatography was performed on Merk silica gel 60 F_254_ plates. Flash chromatography was performed on Merk silica gel (60 Å, 230–400 mesh). ^1^H NMR spectra and 2D-NOESY spectra were recorded on a Bruker ARX400 (400 MHz) spectrometer. Chemical shifts are reported in ppm using residual CDCl_3_ or CD_3_OD as the internal standard (CDCl_3_ at 7.27 ppm and CD_3_OD at 3.31 ppm, respectively). ^13^C NMR spectra were recorded on a Bruker 400ARX (101 MHz) spectrometer. Chemical shifts are reported in ppm using residual CDCl_3_ or CD_3_OD as the internal standard (CDCl_3_ at 77.0 ppm and CD_3_OD at 49.0 respectively). NMR data are reported as multiplicity (s = singlet, d = doublet, t = triplet, q = quartet, m = multiplet), coupling constants in Hz, integration. ESI mass spectra were performed by a Bruker Esquire 3000+ instrument equipped with a MS detector composed by a ESI ionization source and a Single Quadrupole mass selective detector or by an Agilent Technologies 1200 Series HPLC system equipped with a DAD and a 6120 MS detector composed by an ESI ionization source and a Single Quadrupole mass selective detector. Optical rotations were measured on a Propol Digital Polarimeter with a sodium lamp.

### Experimental Procedures

#### 2-(Trimethylsilyl)ethyl (*S*)-5-oxopyrrolidine-2-carboxylate (**7**)

To a suspension of (*S*)-pyroglutamic acid (15 g, 0.12 mol, 1 equiv) in toluene (150 mL) were added 2-(trimethysilyl)ethanol (24.3 mL, 0.17 mol, 1.4 equiv) and *p*-toluensulfonic acid monohydrate (2.9 g, 0.015 mol, 0.11 equiv). The mixture was heated (oil bath) to 130 °C and water was removed by azeotropic distillation with a Dean–Stark apparatus for 2 h. After removal of the solvent, the resulting residue was dissolved in EtOAc (100 mL) and washed with a 10% K_2_CO_3_ solution (3 × 50 mL) and brine (1 × 50 mL). The organic layer was dried over anhydrous Na_2_SO_4_, filtered, and concentrated under reduced pressure affording **7** (23.2 g, quantitative) as a white solid, which was used in the next step without any further purification. Spectral data match with those reported in literature.^7^

#### 1-Allyl 2-(2-(trimethylsilyl)ethyl) (*S*)-5-oxopyrrolidine-1,2-dicarboxylate (**5**)

To a solution of **7** (2.0 g, 8.7 mmol, 1 equiv) in dry THF (100 mL), cooled to 0 °C and under a N_2_ atmosphere, was added portion wise NaH (60% in mineral oil, 384 mg, 9.6 mmol, 1.1 equiv). After stirring for 30 min, allylchloroformate (1.11 mL, 10.5 mmol, 1.2 equiv) was added and the reaction was stirred for additional 2 h at the same temperature. The reaction was quenched with saturated NH_4_Cl (50 mL), diluted with EtOAc (70 mL), and the phases were separated. The organic layer was washed with brine (1 × 50 mL), dried over anhydrous Na_2_SO_4_, filtered, and concentrated under reduced pressure. The crude was purified by FC (60:40 Hex/EtOAc) to give **5** (2.32 g, 85%) as a yellow oil. *R*_*f*_ 0.61 (60:40 Hex:EtOAc); [α]_D_^20^ −36.6 (*c* = 1.00, CHCl_3_); ^1^H NMR (400 MHz, CDCl3) δ 5.96–5.83 (m, 1H), 5.34 (dd, *J* = 17.2 Hz, 1.4 Hz, 1H), 5.21 (dd, *J* = 10.5 Hz, 1.4 Hz, 1H), 4.70–4.66 (m, 2H), 4.61 (dd, *J* = 9.4 Hz, 2.7 Hz, 1H), 4.24–4.18 (m, 2H), 2.65–2.55 (m, 1H), 2.50–2.42 (m, 1H), 2.37–2.25 (m, 1H), 2.08–1.98 (m, 1H), 0.99–0.94 (m, 2H), −0.03 (s, 9H); ^13^C{^1^H} NMR (101 MHz, CDCl_3_) δ 172.9, 171.2, 151.0, 131.3, 119.0, 67.4, 64.4, 58.9, 31.2, 21.9, 17.5, −1.5; MS-(ESI) *m*/*z* 335.9 [M + Na]^+^, 351.8 [M + K]^+^. Anal. Calcd for C_14_H_23_NO_5_Si: C 53.65; H 7.40; N 4.47; O 25.52; Si 8.96. Found: C 53.63; H 7.40; N 4.45; O 25.49; Si 8.95.

#### 1-Allyl 2-(2-(trimethylsilyl)ethyl) (*E*)-4-((dimethylamino)methylene)-5-oxopyrrolidine-1,2-dicarboxylate (**8**)

To a solution of **5** (2.186 g, 7 mmol, 1 equiv) in dimethoxyethane (6 mL) was added Bredereck’s reagent (2.16 mL, 10.5 mmol, 1.5 equiv) and the reaction was heated (oil bath) to 70 °C for 2.5 h. After concentration at reduced pressure, the crude was purified by FC (100% EtOAc) to give **8** (2.24 g, 87%) as a yellow oil. *R*_*f*_ 0.50 (EtOAc); [α]_D_^20^ −33.6 (*c* = 1.00, CHCl_3_); ^1^H NMR (400 MHz, CDCl_3_) δ 7.07 (bt, *J* = 1.5 Hz, 1H), 5.92–5.81 (m, 1H), 5.33 (dd, *J* = 17.2, 1.6 Hz, 1H), 5.15 (dd, *J* = 10.5, 1.6 Hz, 1H), 4.66–4.62 (m, 2H), 4.51 (dd, *J* = 10.6 Hz, 3.6 Hz, 1H), 4.19–4.13 (m, 2H), 3.22 (dd, *J* = 14.5, 11.8 Hz, 1H), 2.96 (s, 6H), 2.84 (dd, *J* = 14.5 Hz, 3.5 Hz, 1H), 0.96–0.91 (m, 2H), −0.03 (s, 9H); ^13^C{^1^H} NMR (101 MHz, CDCl_3_) δ 171.8, 168.9, 152.0, 146.7, 131.8, 118.0, 90.6, 66.5, 63.8, 55.8, 41.9, 26.4, 17.3, −1.6; MS-(ESI) *m*/*z* 390.9 [M + Na]^+^, 406.8 [M + K]^+^. Anal. Calcd for C_17_H_28_N_2_O_5_Si: C 55.41, H 7.66, N 7.60, O 21.71, Si 7.62. Found: C 55.39, H 7.69, N 7.61, O 21.73, Si 7.60.

#### Synthesis of 1-Allyl 2-(2-(trimethylsilyl)ethyl) (2*S*)-4-((dimethylamino)methyl)-5-oxopyrrolidine-1,2-dicarboxylate (**9**)

To a solution of **8** (1.95 g, 5.3 mmol, 1 equiv) in DCM (40 mL), was added AcOH (4 mL). After cooling to 0 °C, was added portion wise NaBH(OAc)_3_ (6.74 g, 32 mmol, 6 equiv). The ice bath was removed and the reaction mixture was stirred at r.t. for 2.5 h. After quenching with water (20 mL), a K_2_CO_3_ 10% solution was added until pH = 9 was reached. The organic layer was separated, dried over anhydrous Na_2_SO_4_, filtered and concentrated at reduced pressure to give a 2:1 mixture of diastereoisomers **9** (1.95 g, quantitative, r.d. determined by ^1^H NMR analysis) as a yellow oil, which was used in the next step without any further purification. *R*_*f*_ 0.40 (90:10 DCM:MeOH); ^1^H NMR (400 MHz, CDCl_3_) 5.87–5.74 (m, 1H), 5.28 (dd, *J* = 17.2, 1.6 Hz, 1H), 5.13 (dd, *J* = 10.5, 1.6 Hz, 1H), 4.66–4.54 (m, 2H), 4.51 (*major*)/4.46 (*minor*) ([dd, *J* = 9.3, 1.9 Hz]/[dd, *J* = 9.2, 6.0 Hz], 1H), 4.18–4.08 (m, 2H), 2.76–2.65 (m, 1H), 2.61 (*major*)/2.52 (*minor*) ([dd, *J* = 12.6, 4.3 Hz]/[dd, *J* = 12.5, 4.6 Hz], 1H), 2.46–2.30 (m, 1H), 2.23–2.14 (m, 1H), 2.11 (*major*)/2.10 (*minor*) ([s/s], 3H), 1.98–1.88 (m, 1H), 0.93–0.86 (m, 2H), −0.07 (s, 9H); ^13^C{^1^H} NMR (101 MHz, CDCl_3_) 173.7 (*minor*), 173.6 (*major*), 171.2 (*minor*), 171.0 (*major*), 150.7, 131.0, 118.6 (*minor*), 118.5 (*major*), 67.1 (*minor*), 67.0 (*major*), 64.0 (*major*), 63.8 (*minor*), 59.8 (*minor*), 59.6 (*major*), 57.4 (*minor*), 57.0 (*major*), 45.6 (*major*), 45.3 (*minor*), 41.5, 40.6, 27.9, 26.1, 17.2, −1.7; MS-(ESI) *m*/*z* 371.1 [M + H]^+^. Anal. Calcd for C_17_H_30_N_2_O_5_Si: C 55.11; H 8.16; N 7.56; O 21.59; Si 7.58. Found: C 55.15; H 8.21; N 7.53; O 21.57; Si 7.56.

#### Synthesis of 1-Allyl 2-(2-(trimethylsilyl)ethyl) (*S*)-4-methylene-5-oxopyrrolidine-1,2-dicarboxylate (**4**)

To a solution of **9** (1.95 g, 5.3 mmol) in methanol (5 mL) was added MeI (10 mL). The reaction mixture was stirred overnight at r.t. After concentration, the residue was dissolved in a 1:4 mixture of Et_3_N/DCM (25 mL) and the reaction was stirred overnight at r.t. The solvents were evaporated and the residue diluted with EtOAc (50 mL). After washing with saturated NaHCO_3_ solution (1 × 30 mL) and 1 M HCl solution (2 × 30 mL), the layers were separated. The organic phase was dried over anhydrous Na_2_SO_4_, filtered, and evaporated to give pure **4** (1.30 g, 75% over the two steps) as a yellow oil. *R*_*f*_ 0.45 (70:30 Hex/EtOAc); [α]_D_^20^ −19.2 (*c* = 1.00, CHCl_3_);. ^1^H NMR (400 MHz, CDCl_3_) δ 6.24 (t, *J* = 2.4 Hz, 1H), 6.0–5.87 (m, 1H), 5.53 (t, *J* = 2.4 Hz, 1H), 5.41 (dd, *J* = 17.2, 1.3 Hz, 1H), 5.25 (dd, *J* = 10.5, 1.3 Hz, 1H), 4.80–4.69 (m, 2H), 4.64 (dd, *J* = 10.2, 3.1 Hz, 1H), 4.27–4.18 (m, 2H), 3.09 (ddt, *J* = 17.5, 10.2, 3.0 Hz, 1H), 2.79–2.68 (m, 1H), 1.02–0.94 (m, 2H), 0.02 (s, 9H).;^13^C{^1^H} NMR (101 MHz, CDCl_3_) δ 170.9, 165.1, 151.6, 136.4, 131.2, 121.5, 119.0, 67.5, 64.5, 55.7, 28.1, 17.5, −1.5; MS-(ESI) *m*/*z* 348.0 [M + Na]^+^, 364.0 [M + K]^+^. Anal. Calcd for C_15_H_23_NO_5_Si: C 55.36; H 7.12; N 4.30; O 24.58; Si 8.63. Found: C 55.90; H 7.14; N 4.28; O 24.57; Si 8.65.

#### Synthesis of Bis(2-phenylpropan-2-yl) 3,3′-disulfanediyl(2*R*,2′*R*)-bis(2-((((9*H*-fluoren-9-yl)methoxy)carbonyl)amino)propanoate) (**11**)

To a suspension of Fmoc-cystine **10** (750 mg, 1.16 mmol, 1 equiv) in dry DCM (20 mL) was added dropwise cumyl trichloroacetimidate^6^ (1.85 g, 6.6 mmol, 6 equiv). After stirring overnight at r.t., the reaction mixture was washed with 0.5% aqueous citric acid solution (3 × 10 mL) and saturated NaHCO_3_ solution (1 × 30 mL). The organic phase was dried over anhydrous Na_2_SO_4_, filtered, and concentrated at reduced pressure. The crude was purified by FC (70:30 Hex/EtOAc) to give **11** (886 mg, 83%) as a white foam. *R*_*f*_ 0.50 (70:30 Hex/EtOAc); [α]_D_^20^ −10.8 (*c* = 1.00, CHCl_3_); ^1^H NMR (400 MHz, CDCl_3_) δ 7.73 (d, *J* = 7.6 Hz, 4H), 7.55 (d, *J* = 6.2 Hz, 4H), 7.38–7.25 (m, 18H), 5.68 (d, *J* = 7.8 Hz, 2H), 4.71–4.63 (m, 2H), 4.40–4.28 (m, 4H), 4.21–4.14 (m, 2H), 3.33–3.10 (m, 4H), 1.82 (s, 6H), 1.80 (s, 6H); ^13^C{^1^H} NMR (100 MHz, CDCl_3_) δ 169.0, 155.9, 144.8, 143.9, 141.4, 128.5, 127.8, 127.6, 127.2, 125.3, 124.5, 120.1, 84.4, 67.4, 54.3, 47.2, 41.9, 28.6, 28.3; MS-(ESI) *m*/*z* + 943.6 [M + Na]^+^, 959.6 [M + K]^+^. Anal. Calcd for C_54_H_52_N_2_O_8_S_2_: C 70.41; H 5.69; N 3.04; O 13.90, S 6.96. Found: C 70.44; H 5.66; N 3.06; O 13.88; S 6.98.

#### 2-Phenylpropan-2-yl (((9*H*-fluoren-9-yl)methoxy)carbonyl)-l-cysteinate (**12**)

To a solution of **11** (811 mg, 0.881 mmol, 1 equiv) in THF (15 mL) and under a N_2_ atmosphere, were added H_2_O (32 μL, 1.76 mmol, 2 equiv) and Bu_3_P (434 μL, 1.76 mmol, 1 equiv). The reaction was stirred for 45 min at r.t. and then quenched with H_2_O (1.5 mL). After concentration under reduced pressure, the crude was purified by FC (80:20 Hex/EtOAc) to give **12** (360 mg, 90%) as a white foam. *R*_*f*_ 0.42 (80:20 Hex/EtOAc); [α]_D_^20^ −2.4 (*c* = 1.00, CHCl_3_); ^1^H NMR(400 MHz, CDCl_3_) δ 7.78 (d, *J* = 7.5 Hz, 2H), 7.60 (d, *J* = 7.5 Hz, 2H), 7.45–7.27 (m, 9H), 5.67 (d, *J* = 7.5 Hz, 1H), 4.70–4.59 (m, 1H), 4.51–4.34 (m, 2H), 4.23 (t, *J* = 7.0 Hz, 1H), 3.13–3.00 (m, 2H), 1.86 (s, 3H), 1.84 (s, 3H); ^13^C{^1^H} NMR (101 MHz, CDCl_3_) δ 168.6, 155.7, 144.7, 143.9, 143.8, 141.4, 128.5, 127.9, 127.6, 127.2, 125.2, 124.5, 120.1, 84.2, 67.2, 55.5, 47.3, 28.6, 28.3, 27.4; MS-(ESI): *m*/*z* 484.0 [M + Na]^+^, 500.0 [M + K]^+^. Anal. Calcd For C_27_H_27_NO_4_S: C 70.26; H 5.90; N 3.03; O 13.86; S 6.95. Found: C 70.27; H 5.87; N 3.00; O 13.85; S 6.97.

#### 1-Allyl 2-(2-(trimethylsilyl)ethyl)(2*S*)-4-(((-2-((((9*H*-fluoren-9-yl)methoxy)carbonyl)amino)-3-oxo-3-((2-phenylpropan-2-yl)oxy)propyl)thio)methyl)-5-oxopyrrolidine-1,2-dicarboxylate (**13**)

To a solution of **4** (375 mg, 1.15 mmol, 1 equiv) in DCM (15 mL), were added **12** (584 mg, 1.27 mmol, 1 equiv) and Et_3_N (16 μL, 0.12 mmol, 0.1 equiv). After stirring for 2 h at r.t., the solvent was concentrated at reduced pressure. The crude was purified by FC (75:25 Hex/EtOAc) to give a 3:1 mixture of diastereoisomers (851 mg, 94%, d.r. determined by ^1^H NMR analysis) as white foam.

**13a** (*major*): *R*_*f*_ 0.26 (75:25 Hex/EtOAc); [α]_D_^20^ −10.5 (*c* = 1.00, CHCl_3_); ^1^H NMR (400 MHz, CDCl_3_) δ 7.76 (d, *J* = 7.5 Hz, 2H), 7.61 (d, *J* = 7.4 Hz, 2H), 7.52–7.17 (m, 9H), 6.04–5.85 (m, 1H), 5.69 (bd, *J* = 7.7 Hz, 1H), 5.41 (dd, *J* = 17.2, 1.4 Hz, 1H), 5.27 (dd, *J* = 10.4, 1.4 Hz, 1H), 4.88–4.68 (m, 2H), 4.70–4.52 (m, 2H), 4.48–4.33 (m, 2H), 4.34–4.18 (m, 3H), 3.20–3.00 (m, 3H), 3.05–2.85 (m, 1H), 2.77 (dd, *J* = 12.7, 8.3 Hz, 1H), 2.45–2.13 (m, 2H), 1.83 (s, 3H), 1.81 (s, 3H), 1.14–0.98 (m, 2H), 0.06 (s, 9H); ^13^C{^1^H} NMR (101 MHz, CDCl_3_) δ 172.8, 170.9, 169.0, 155.8, 150.8, 144.7, 143.8, 141.3, 131.1, 128.4, 127.7, 127.4, 127.1, 125.2, 124.4, 120.0, 119.0, 84.1, 67.4, 67.2, 64.5, 57.0, 54.4, 47.1, 42.3, 35.6, 33.3, 28.5, 28.2, 27.9, 17.5, −1.5; MS-(ESI) *m*/*z* 809.5 [M + Na]^+^, 825.4 [M + K]^+^. Anal. Calcd For C_42_H_50_N_2_O_9_SSi: C 64.10; H 6.40; N 3.56; O 18.30; S 4.07; Si 3.57. Found: C 64.07; H 6.43; N 3.55; O 18.33; S 4.08; Si 3.55.

**13b** (*minor*): *R*_*f*_ 0.13 (75:25 Hex/EtOAc); [α]_D_^20^ +14.9 (*c* = 1.00, CHCl_3_); ^1^H NMR (400 MHz, CDCl_3_) δ 7.76 (d, *J* = 7.5 Hz, 2H), 7.60 (d, *J* = 7.4 Hz, 2H), 7.51–7.21 (m, 9H), 6.05–5.87 (m, 1H), 5.57 (bd, *J* = 7.5 Hz, 1H), 5.42 (dd, *J* = 17.2, 1.4 Hz, 1H), 5.28 (dd, *J* = 10.5, 1.4 Hz, 1H), 4.83–4.69 (m, 2H), 4.58–4.55 (m, 1H), 4.52 (dd, *J* = 8.8, 6.7 Hz, 1H*)*, 4.44–4.35 (m, 2H), 4.34–4.19 (m, 3H), 3.23–2.97 (m, 3H), 2.91–2.75 (m, 1H), 2.77–2.62 (m, 1H), 2.61–2.47 (m, 1H), 2.04–1.89 (m, 1H), 1.83 (s, 3H), 1.81 (s, 3H), 1.09–0.96 (m, 2H), 0.05 (s, 9H); ^13^C{^1^H} NMR (101 MHz, CDCl_3_) δ 172.7, 171.2, 169.0, 155.8, 151.0, 144.8, 143.9, 141.4, 131.1, 128.5, 127.8, 127.5, 127.2, 125.2, 124.5, 120.1, 119.2, 84.3, 67.6, 67.3, 64.5, 57.3, 54.3, 47.2, 43.2, 35.4, 34.2, 28.7, 28.2, 26.9, 17.5, −1.4; MS-(ESI) *m*/*z* 809.5 [M + Na]^+^, 825.4 [M + K]^+^. Anal. Calcd for C_42_H_50_N_2_O_9_SSi: C 64.10; H 6.40; N 3.56; O 18.30; S 4.07; Si 3.57. Found: C 64.12; H 6.42; N 3.57; O 18.34; S 4.06; Si 3.59.

#### 1-Allyl 2-(2-(trimethylsilyl)ethyl)(2*S*)-4-(((-2-((((9*H*-fluoren-9-yl)methoxy)carbonyl)amino)-3-oxo-3-((2-phenylpropan-2-yl)oxy)propyl)thio)methyl)-4-azido-5-oxopyrrolidine-1,2-dicarboxylate (**3**)

To a solution of **13a** and **13b** (691 mg, 0.88 mmol, 1 equiv, d.r. 3:1) in dry THF (25 mL) cooled to −78 °C and under a N_2_ atmosphere, was added dropwise LiHMDS (1 M in THF,1.94 mL, 1.94 mmol, 2.2 equiv). After stirring for 30 min at −78 °C, was added dropwise a solution of trisyl azide^13^**14** (354 mg, 1.14 mmol, 1.3 equiv) in dry THF (10 mL). The reaction mixture was stirred at −78 °C for 1 h and then quenched with AcOH (232 μL, 4.06 mmol). After stirring at r.t. overnight, AcOEt (25 mL) and water (25 mL) were added. The layers were separated and the organic phase was dried over anhydrous Na_2_SO_4_, filtered, and concentrated under reduced pressure. The crude was purified by FC (90:10 to 50:1 Hex/EtOAc) to give a 1.8:1 mixture of epimers (435 mg, 60%, d.r. determined by ^1^H NMR analysis) as white foams.

**3a**: *R*_*f*_ 0.55 (75:25 Hex:EtOAc), [α]_D_^20^ +26.3 (*c* = 1.00, CHCl_3_), ^1^H NMR (400 MHz, CDCl_3_) δ 7.77 (d, *J* = 7.5 Hz, 2H), 7.60 (d, *J* = 7.5 Hz, 2H), 7.48–7.24 (m, 9H), 6.04–5.86 (m, 1H), 5.58 (bd, *J* = 7.5 Hz, 1H), 5.44 (dd, *J* = 17.2, 1.3 Hz, 1H), 5.32 (dd, *J* = 10.6, 1.3 Hz, 1H), 4.85–4.72 (m, 2H), 4.71–4.54 (m, 2H), 4.51–4.35 (m, 2H), 4.34–4.20 (m, 3H), 3.33–3.15 (m, 2H), 3.16–2.98 (m, 2H), 2.39 (dd, *J* = 14.0, 5.8 Hz, 1H), 2.30 (dd, *J* = 14.0, 9.0 Hz, 1H), 1.85 (s, 3H), 1.83 (s, 3H), 1.10–0.97 (m, 2H), 0.05 (s, 9H); ^13^C{^1^H} NMR (101 MHz, CDCl_3_) δ 170.4, 169.5, 168.9, 155.8, 150.7, 144.7, 143.9, 141.4, 130.8, 128.5, 127.8, 127.6, 127.2, 125.2, 124.5, 120.1, 119.6, 84.4, 68.1, 67.5, 67.4, 64.9, 55.7, 54.6, 47.2, 37.1, 36.4, 31.9, 28.7, 28.2, 17.5, −1.4; MS – (ESI) *m*/*z* 850.7 [M + Na]^+^. Anal. Calcd For C_42_H_49_N_5_O_9_SSi: C 60.92; H 5.97; N 8.46; O 17.39; S 3.87; Si 3.39. Found: C 60.90; H 5.95; N 8.47; O 17.40; S 3.88; Si 3.37.

**3b**: *R*_*f*_ 0.42 (75:25 Hex:EtOAc) [α]_D_^20^ −36.8 (*c* = 1.00, CHCl_3_); ^1^H NMR (400 MHz, CDCl_3_) δ 7.76 (d, *J* = 7.5 Hz, 2H), 7.60 (d, *J* = 7.5 Hz, 2H), 7.53–7.11 (m, 9H), 6.05–5.85 (m, 1H), 5.60 (bd, *J* = 7.5 Hz, 1H), 5.43 (dd, *J* = 17.2, 1.4 Hz, 1H), 5.29 (dd, *J* = 10.5, 1.4 Hz, 1H), 4.89–4.74 (m, 2H), 4.70–4.52 (m, 2H), 4.48–4.17 (m, 5H), 3.28–2.96 (m, 4H), 2.58 (dd, *J* = 14.0, 9.9 Hz, 1H), 2.11 (dd, *J* = 14.0, 2.3 Hz, 1H), 1.85 (s, 3H), 1.82 (s, 3H), 1.12–0.97 (m, 2H), 0.05 (s, 9H); ^13^C{^1^H} NMR (101 MHz, CDCl_3_) δ 169.8, 169.5, 168.9, 155.9, 150.6, 144.7, 143.9, 141.4, 130.9, 128.5, 127.8, 127.6, 127.2, 125.2, 124.5, 120.1, 119.4, 84.4, 68.0, 67.4, 67.3, 64.8, 56.0, 54.5, 47.2, 36.9, 36.5, 32.4, 28.7, 28.2, 17.4, −1.5; MS-(ESI) *m*/*z* 850.7 [M + Na]^+^ . Anal. Calcd For C_42_H_49_N_5_O_9_SSi: C 60.92; H 5.97; N 8.46; O 17.39; S 3.87; Si 3.39. Found: C 60.95; H 5.99; N 8.48; O 17.36; S 3.88; Si 3.41.

#### (2*S*,4*S*)-2-((((*R*)-2-((((9*H*-Fluoren-9-yl)methoxy)carbonyl)amino)-3-oxo-3-((2-phenylpropan-2-yl)oxy)propyl)thio)methyl)-4-(((allyloxy)carbonyl)amino)-2-azido-5-oxo-5-(2-(trimethylsilyl)ethoxy)pentanoic acid (**15b**)

To a solution of **3b** (178 mg, 0.22 mmol, 1 equiv) in a 5:2 mixture of THF/H_2_O (7 mL), cooled to 0 °C, was added a 1 M aqueous solution of LiOH·H_2_O (258 μL, 0.26 mmol, 1.2 equiv). After stirring at the same temperature for 1 h, a 1 M aqueous solution of HCl was added until pH = 2 was reached. AcOEt (25 mL) was added and the layers were separated. The organic phase was washed with water (25 mL), dried over anhydrous Na_2_SO_4_, and concentrated at reduced pressure. The crude was purified by FC (100% DCM to 95:5 DCM/MeOH) to give **15b** (171 mg, 92%) as a white foam *R*_*f*_ 0.38 (95:5 DCM:MeOH); [α]_D_^20^ +40.0 (*c* = 1.00, CHCl_3_); ^1^H NMR (400 MHz, MeOD) δ 7.75 (d, *J* = 7.5 Hz, 2H), 7.62 (d, *J* = 7.5 Hz, 2H), 7.47–7.11 (m, 9H), 5.96–5.81 (m, 1H), 5.28 (d, *J* = 17.2 Hz, 1H), 5.14 (dd, *J* = 10.5, 1.0 Hz, 1H), 4.53–4.32 (m, 6H), 4.21–4.16 (m, 3H), 3.27–3.08 (m, 2H), 3.08–2.88 (m, 2H), 2.51–2.38 (m, 1H), 2.24 (dd, *J* = 14.5, 8.6 Hz, 1H), 1.76 (s, 6H), 1.05–0.92 (m, 2H), 0.01 (s, 9H); ^13^C{^1^H} NMR (101 MHz, MeOD) δ 173.6, 172.9, 170.9, 158.3, 158.0, 146.6, 145.1, 142.5, 134.1, 129.2, 128.7, 128.2, 128.0, 126.3, 125.4, 120.9, 117.6, 84.6, 68.2, 66.7, 64.8, 61.4, 56.4, 52.6, 48.3, 41.1, 38.6, 36.2, 29.0, 28.9, 18.1, −1.5; MS-(ESI) *m*/*z* 844.7 [M–H]^−^. Anal. Calcd For C_42_H_51_N_5_O_10_SSi: C 59.63; H 6.08; N 8.28; O 18.91; S 3.79; Si 3.32. Found: C 59.65; H 6.06; N 8.26; O 18.96; S 3.76; Si 3.31.

#### (2*R*,4*S*)-2-((((*R*)-2-((((9*H*-Fluoren-9-yl)methoxy)carbonyl)amino)-3-oxo-3-((2-phenylpropan-2-yl)oxy)propyl)thio)methyl)-4-(((allyloxy)carbonyl)amino)-2-azido-5-oxo-5-(2-(trimethylsilyl)ethoxy)pentanoic acid (**15a**)

**15a** was prepared following the same procedure reported for **15b**.Yellow foam. Yield 84%; White foam. *R*_*f*_ 0.38 (95:5 DCM:MeOH), [α]_D_^20^ +12.8 (*c* = 1.00 CHCl_3_) ^1^H NMR (400 MHz, MeOD) δ 7.75 (d, *J* = 7.5 Hz, 2H), 7.64 (d, *J* = 7.4 Hz, 2H), 7.47–7.11 (m, 9H), 5.98–5.83 (m, 1H), 5.29 (dd, *J* = 17.2, 1.4 Hz, 1H), 5.15 (d, *J* = 10.5 Hz, 1H), 4.56–4.40 (m, 4H), 4.38–4.27 (m, 2H), 4.27–4.11 (m, 3H), 3.26–3.11 (m, 2H), 3.11–2.91 (m, 2H), 2.44–2.22 (m, 2H), 1.76 (s, 6H), 1.05–0.92 (m, 2H), 0.01 (s, 9H); ^13^C{^1^H} NMR (101 MHz, MeOD) δ 173.4, 172.9, 170.8, 158.3, 158.0, 146.5, 145.1, 142.5, 134.1, 129.3, 128.7, 128.2, 126.3, 125.4, 120.9, 117.6, 84.7, 70.3, 68.2, 66.7, 64.9, 56.3, 52.2, 48.3, 41.9, 38.8, 36.2, 29.0, 28.9, 18.1, −1.4; MS-(ESI) *m*/*z* 844.7 [M–H]^−^. Anal. Calcd For C_42_H_51_N_5_O_10_SSi: C 59.63; H 6.08; N 8.28; O 18.91; S 3.79; Si 3.32. Found: C 59.66; H 6.05; N 8.25; O 18.90; S 3.81; Si 3.30.

#### 9-(4-Nitrobenzyl)-5-(2-phenylpropan-2-yl)-11-(2-(trimethylsilyl)ethyl)(5*R*,9*S*,11*S*)-9-azido-1-(9*H*-fluoren-9-yl)-3,13-dioxo-2,14-dioxa-7-thia-4,12-diazaheptadec-16-ene-5,9,11-tricarboxylate (**16b**)

To a solution of **15b** (152 mg, 0.18 mmol, 1 equiv) in dry DMF (2 mL), cooled to 0 °C and under a N_2_ atmosphere, were added K_2_CO_3_ (30 mg, 0.22 mmol,1.2 equiv), KI (60 mg, 0.36 mmol,2 equiv), and 4-nitrobenzyl bromide (77 mg, 0.36 mmol, 2 equiv). After stirring at r.t. overnight, the reaction was quenched with a 1 M HCl (1 mL), diluted with EtOAc (15 mL), and the layers were separated. The organic phase was washed with water (2 × 5 mL) and brine (3 × 5 mL), dried over anhydrous Na_2_SO_4_, filtered, and concentrated at reduced pressure. The crude was purified by FC (100% Hexane to 7:3 Hex/EtOAc) to give **16b** (130 mg, 74%) as a yellow foam. *R*_*f*_ 0.60 (7:3 Hex:EtOAc); [α]_D_^20^ +13.1 (*c* = 1.00, CHCl_3_) ^1^H NMR (400 MHz, CDCl_3_) δ 8.19 (d, *J* = 8.7 Hz, 2H), 7.75 (d, *J* = 7.5 Hz, 2H), 7.58 (d, *J* = 7.3 Hz, 2H), 7.51 (d, *J* = 8.7 Hz, 2H), 7.42–7.27 (m, 9H), 5.92–5.79 (m, 1H), 5.63 (d, *J* = 7.7 Hz, 1H), 5.36 (d, *J* = 8.1 Hz, 1H), 5.32–5.15 (m, 4H), 4.60–4.15 (m, 9H), 3.20–3.01 (m, 4H), 2.58 (dd, *J* = 14.7, 5.0 Hz, 1H), 2.29 (dd, *J* = 14.7, 6.9 Hz, 1H), 1.82 (s, 3H), 1.80 (s, 3H), 1.06–0.97 (m, 2H), 0.05 (s, 9H); ^13^C{^1^H} NMR (101 MHz, CDCl_3_) δ 171.1, 170.1, 168.8, 155.8, 155.6, 148.0, 144.7, 143.8, 141.6, 141.4, 132.4, 128.8, 128.5, 127.8, 127.5, 127.2, 125.2, 124.4, 124.0, 120.1, 118.2, 84.3, 68.3, 67.4, 66.8, 66.2, 64.7, 54.6, 50.9, 47.2, 40.4, 37.8, 36.4, 28.6, 28.2, 17.4, −1.4; MS-(ESI) *m*/*z* 1003.6 [M + Na]^+^, 1019.6 [M + K]^+^. Anal. Calcd For C_49_H_56_N_6_O_12_SSi: C 59.98; H 5.75; N 8.57; O 19.57; S 3.27; Si 2.86. Found: C 59.98; H 5.72; N 8.60; O 19.53; S 3.29; Si 2.85.

#### 9-(4-Nitrobenzyl)-5-(2-phenylpropan-2-yl)-11-(2-(trimethylsilyl)ethyl)(5*R*,9*R*,11*S*)-9-azido-1-(9*H*-fluoren-9-yl)-3,13-dioxo-2,14-dioxa-7-thia-4,12-diazaheptadec-16-ene-5,9,11-tricarboxylate (**16a**)

**16a** was prepared following the same procedure reported for **16b**. Yield 75%. Yellow foam. *R*_*f*_ 0.60 (7:3 Hex:EtOAc) [α]_D_^20^ +2.3 (*c* = 1.00, CHCl_3_) ^1^H NMR (400 MHz, CDCl_3_) δ 8.18 (d, *J* = 8.6 Hz, 2H), 7.76 (d, *J* = 7.5 Hz, 2H), 7.59 (d, *J* = 7.5 Hz, 2H), 7.51 (d, *J* = 8.7 Hz, 2H), 7.44–7.25 (m, 9H), 5.94–5.77 (m, 1H), 5.63 (bd, *J* = 7.4 Hz, 1H), 5.35–5.22 (m, 3H), 5.19 (dd, *J* = 10.4, 1.1 Hz, 1H), 5.13 (bd, *J* = 8.5 Hz, 1H), 4.66–4.44 (m, 4H), 4.42–4.31 (m, 2H), 4.28–4.14 (m, 3H), 3.30–2.97 (m, 4H), 2.43–2.20 (m, 2H), 1.81 (s, 3H), 1.80 (s, 3H), 1.03–0.92 (m, 2H), 0.04 (s, 9H); ^13^C{^1^H} NMR (101 MHz, CDCl_3_) δ 171.3, 170.3, 168.8, 155.8, 155.6, 148.0, 144.7, 143.8, 141.6, 141.3, 132.4, 128.8, 128.5, 127.8, 127.5, 127.2, 125.1, 124.4, 123.9, 120.1, 118.1, 84.3, 68.6, 67.3, 66.8, 66.1, 64.5, 54.5, 50.8, 47.2, 41.6, 38.6, 36.4, 28.5, 28.2, 17.4, −1.5; MS-(ESI) *m*/*z* 1003.6 [M + Na]^+^, 1019.5 [M + K]^+^. Anal. Calcd For C_49_H_56_N_6_O_12_SSi: C 59.98; H 5.75; N 8.57; O 19.57; S 3.27; Si 2.86. Found: C 59.97; H 5.76; N 8.59; O 19.55; S 3.26; Si 2.88.

#### *N*-(((9*H*-Fluoren-9-yl)methoxy)carbonyl)-*S*-((2*S*,4*S*)-4-(((allyloxy)carbonyl)amino)-2-azido-2-(((4-nitrobenzyl)oxy)carbonyl)-5-oxo-5-(2-(trimethylsilyl)ethoxy)pentyl)-l-cysteine (**2**)

**16b** (202 mg, 0.21 mmol, 1 equiv) was dissolved in a 2% vol solution of TFA in DCM (5 mL) and stirred for 30 min at r.t. After concentration at reduced pressure, the crude was purified by FC (1:9 Hex:EtOAc) to give **2** (178 mg, 98%) as a yellow foam. *R*_*f*_ 0.63 (1:9 Hex:EtOAc); [α]_D_^20^ −10.3 (*c* = 1.00, CHCl_3_); ^1^H NMR (400 MHz, MeOD) δ 8.18 (d, *J* = 8.8 Hz, 2H), 7.77 (d, *J* = 7.5 Hz, 2H), 7.70–7.55 (m, 4H), 7.37 (t, *J* = 7.4 Hz, 2H), 7.29 (t, *J* = 7.4 Hz, 2H), 5.95–5.82 (m, 1H), 5.35–5.23 (m, 3H), 5.15 (dd, *J* = 10.5, 1.4 Hz, 1H), 4.54–4.45 (m, 2H), 4.44–4.13 (m, 7H), 3.24–3.06 (m, 3H), 2.99–2.87 (m, 1H), 2.56 (dd, *J* = 14.7, 4.3 Hz, 1H), 2.28 (dd, *J* = 14.7, 8.4 Hz, 1H), 1.01–0.92 (m, 2H), 0.02 (s, 9H); ^13^C{^1^H} NMR (101 MHz, MeOD) δ 172.8, 171.3, 158.4, 158.1, 149.2, 145.3, 145.2, 143.7, 142.6, 134.1, 130.0, 128.8, 128.2, 126.4, 124.7, 120.9, 117.7, 79.5, 70.3, 68.2, 67.8, 66.8, 65.1, 52.0, 40.5, 38.6, 36.6, 18.1, −1.5; MS-(ESI) *m*/*z* 861.4 [M–H]^−^. Anal. Calcd For C_40_H_46_N_6_O_12_SSi: C 55.67; H 5.37; N 9.74; O 22.25; S 3.72; Si 3.25. Found: C 55.69; H 5.35; N 9.73; O 22.26; S 3.74; Si 3.26.

#### *N*-(((9*H*-Fluoren-9-yl)methoxy)carbonyl)-*S*-((2*R*,4*S*)-4-(((allyloxy)carbonyl)amino)-2-azido-2-(((4-nitrobenzyl)oxy)carbonyl)-5-oxo-5-(2-(trimethylsilyl)ethoxy)pentyl)-l-cysteine (**2a**)

**2a** was prepared following the same procedure reported for **2**. Yield 96%. Yellow foam; *R*_*f*_ 0.63 (1:9 Hex:EtOAc); [α]_D_^20^ +6.1 (*c* = 1.00 CHCl_3_); ^1^H NMR (400 MHz, MeOD) δ 8.10 (d, *J* = 8.4 Hz, 2H), 7.72 (d, *J* = 7.5 Hz, 2H), 7.61 (d, *J* = 7.5 Hz, 2H), 7.54 (d, *J* = 8.4 Hz, 2H), 7.34 (t, *J* = 7.4 Hz, 2H), 7.25 (t, *J* = 7.4 Hz, 2H), 5.93–5.78 (m, 1H), 5.35–5.16 (m, 3H), 5.13 (dd, *J* = 10.5, 1.5 Hz, 1H),4.51–4.36 (m, 4H), 4.36–4.10 (m, 5H), 3.31–3.25 (m, 1H), 3.20–3.07 (m, 2H), 2.95 (dd, *J* = 13.9, 8.4 Hz, 1H), 2.44–2.23 (m, 2H), 1.00–0.90 (m, 2H), 0.00 (s, 9H); ^13^C{^1^H} NMR (101 MHz, MeOD) δ 173.5, 172.8, 171.3, 158.2, 158.0, 149.0, 145.1, 143.6, 142.4, 134.0, 129.8, 128.7, 128.1, 126.3, 124.6, 120.9, 117.8, 79.4, 70.0, 68.2, 67.7, 66.7, 65.0, 51.8, 48.2, 42.4, 39.6, 36.7, 18.1, −1.5; MS-(ESI) *m*/*z* 861.4 [M–H]^−^. Anal. Calcd For C_40_H_46_N_6_O_12_SSi: C 55.67; H 5.37; N 9.74; O 22.25; S 3.72; Si 3.25. Found: C 55.65; H 5.35; N 9.72; O 22.27; S 3.75; Si 3.23.

## Supporting Information Available

Copies of ^1^H, ^13^C, 2D-NOESY experiments and mass spectra for all new compounds (PDF)
