# Supplementary material for: Synthesis of Orthogonally Protected Labionin
Source: J Org Chem. 2021 Feb 18;86(5):4313–9. doi: 10.1021/acs.joc.0c02922 (PMC9552182; doi:10.1021/acs.joc.0c02922)
Supplement: Supplementary file 1 — jo0c02922_si_001.pdf [file jo0c02922_si_001.pdf]

Synthesis of Orthogonally Protected Labionin

Eliana Lo Presti, [§] Alessandro Volonterio[†,§], Monica Sani*[,§]

[§]National Research Council, Institute of Chemical Sciences and Technologies “Giulio Natta” (SCITEC), Via Mario Bianco 9, 20131 Milan, Italy.

[†]Department of Chemistry, Materials, and Chemical Engineering “Giulio Natta”, Politecnico di Milano, via Mancinelli 7, 20141 Milano, Italy.

monica.sani@polimi.it

Table of contents

2D-NOESY Spectra of 3a and 3b S2

^{1}H NMR and ^{13}C NMR Spectra of compounds S5

Mass Spectra of compounds S21

2D-NOESY Spectra of 3a and 3b

Absolute stereochemistry for compound **3a** and **3b** was assigned by 2D-NOESY-NMR spectroscopy. In both derivatives, the relative position of Ha and Hb with respect to the lactam ring plan was identified by measuring their coupling constants with Hc. In derivative **3a**, Ha exhibits a J_{ab} =14.1 Hz and a J_{ac} = 9.4 Hz, while Hb shows a significantly lower J_{bc} = 4.9 Hz, suggesting that Hb is oriented on the opposite face of the pyroglutamic plan with respect to Hc. The presence of a NOE effect between Hb and Hd indicates that they are positioned on the same face of the molecular plan, permitting the attribution of the absolute stereochemistry of the newly generated quaternary carbon center as *R* (Figure S1 and S2). The same approach was used for the derivative **3b**. Hb shows a J_{ba} = 13.9 Hz and a J_{bc} = 2.3 Hz, while Ha showed a J_{ab} of 13.9 Hz and a J_{ac} = 9.7 Hz, suggesting that Ha is oriented on the same face of the pyroglutamic plan with respect to Hc. The presence of a NOE effect between Ha and Hd indicates that they are positioned on the same face of the molecular plan, permitting the attribution of the absolute stereochemistry of the newly generated quaternary carbon center as *S* (Figure S1 and S3).

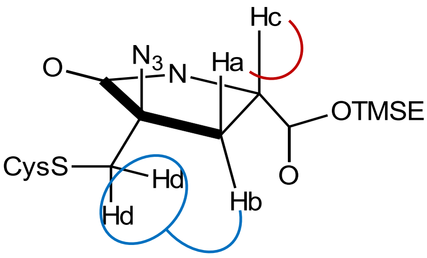

3a

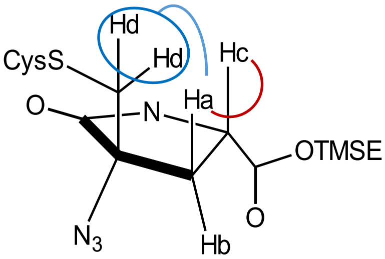

3b

Figure S1: Skew representation of **3a** and **3b**.

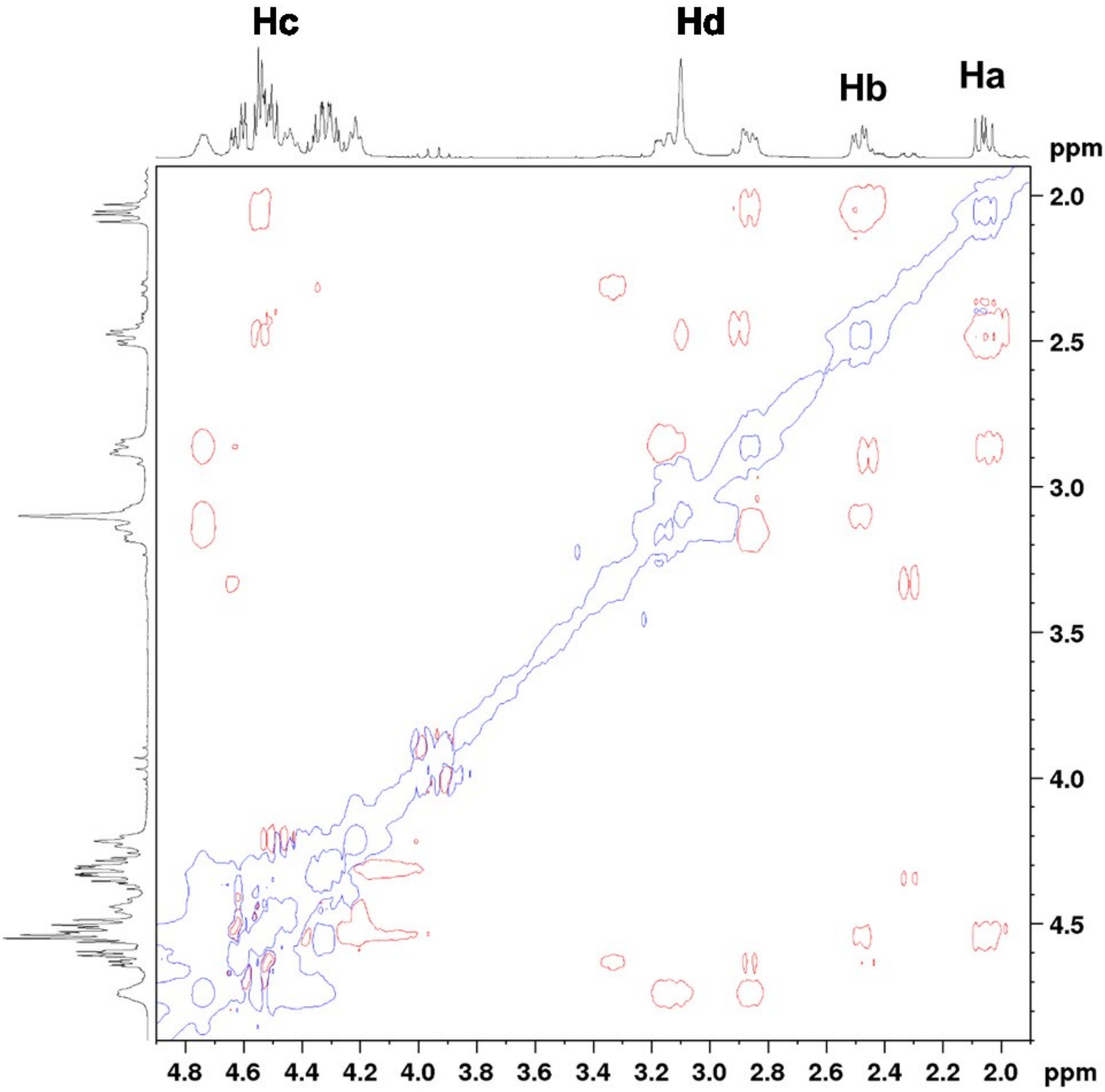

Figure S2: 2D-NOESY-NMR spectrum of compound **3a**, benzene d[6], mixing time 800 ms.

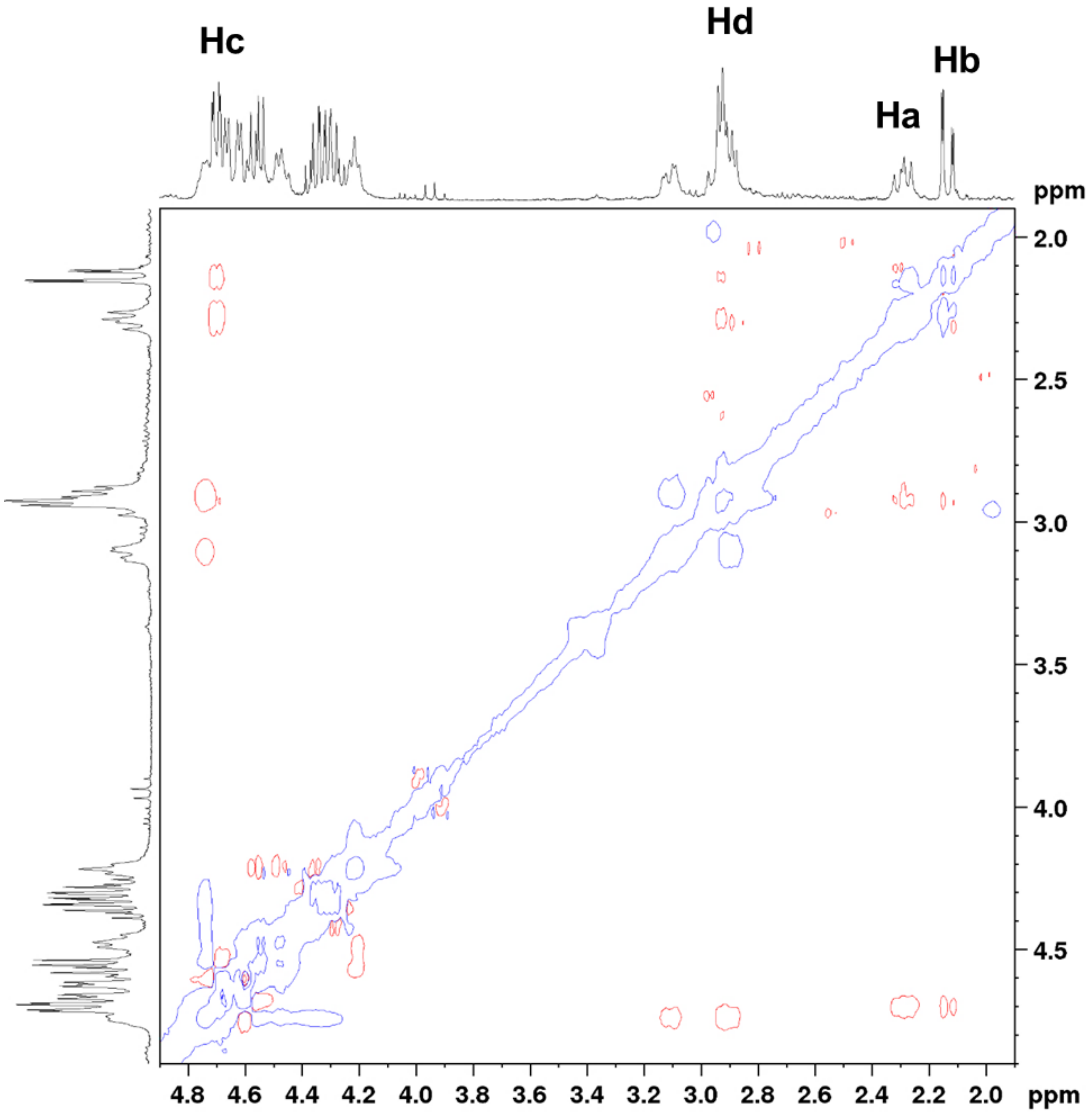

Figure S3: 2D-NOESY-NMR spectrum of compound **3b**, benzene d[6], mixing time 800 ms.

^{1}H NMR and ^{13}C NMR Spectra of compounds

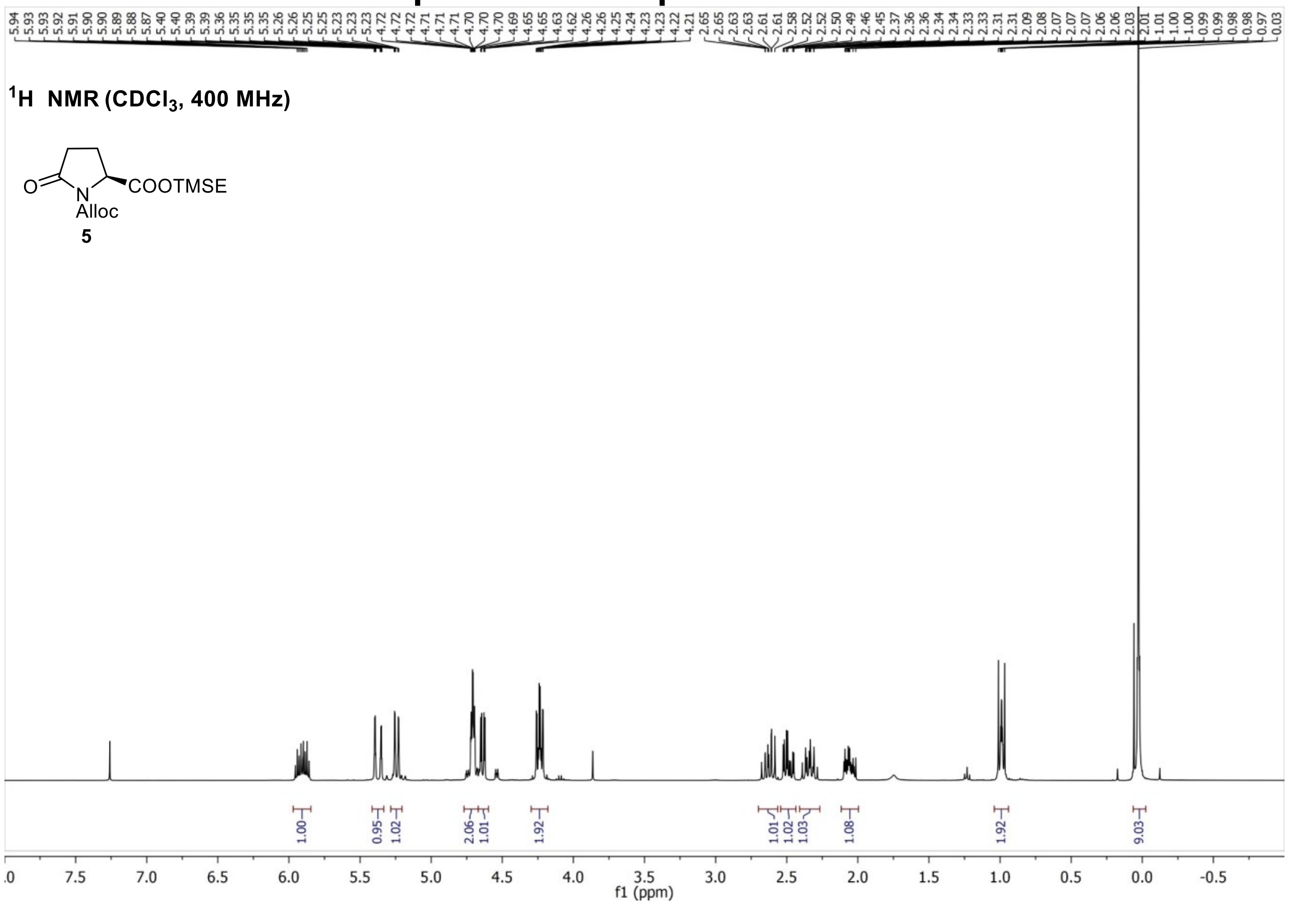

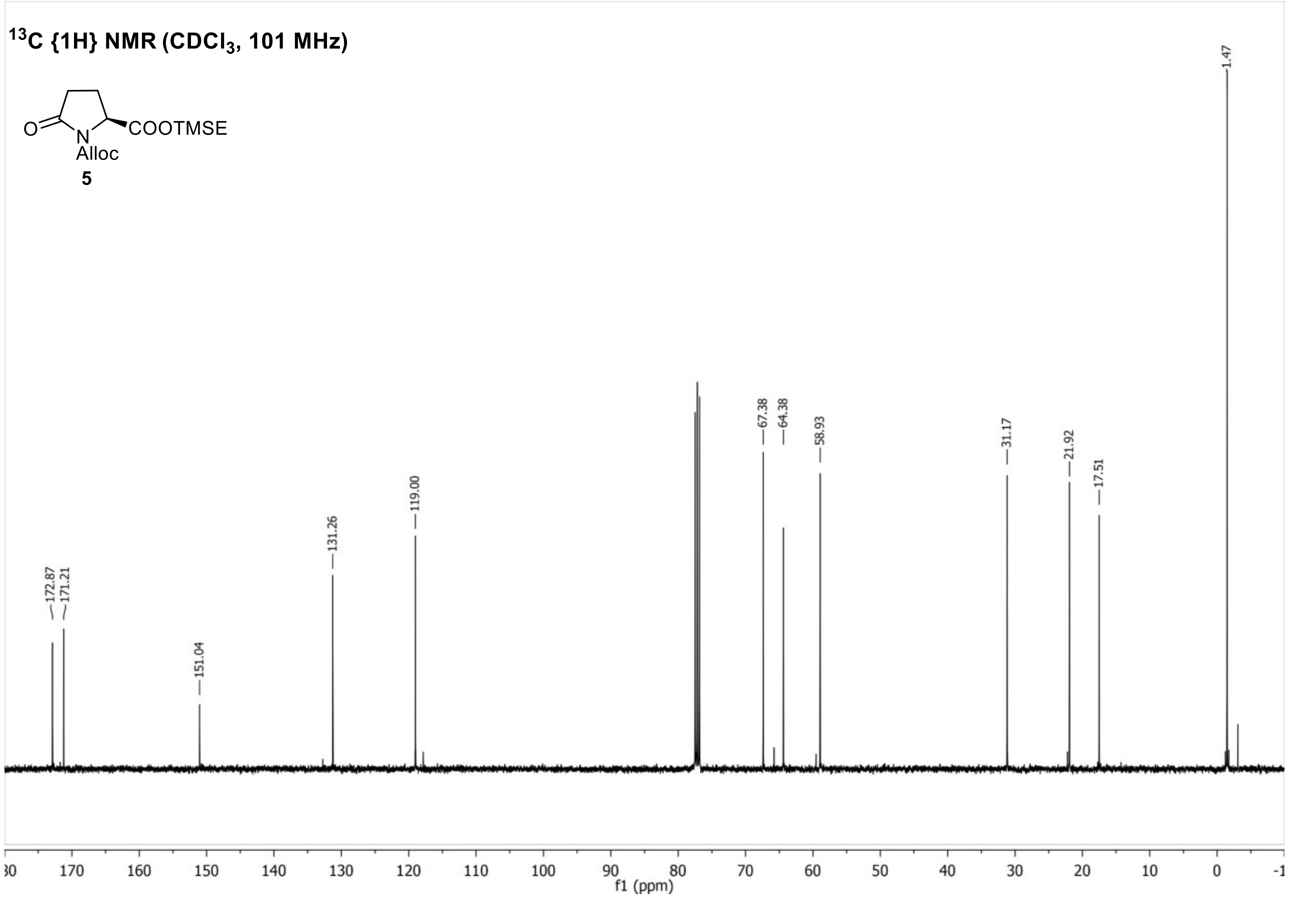

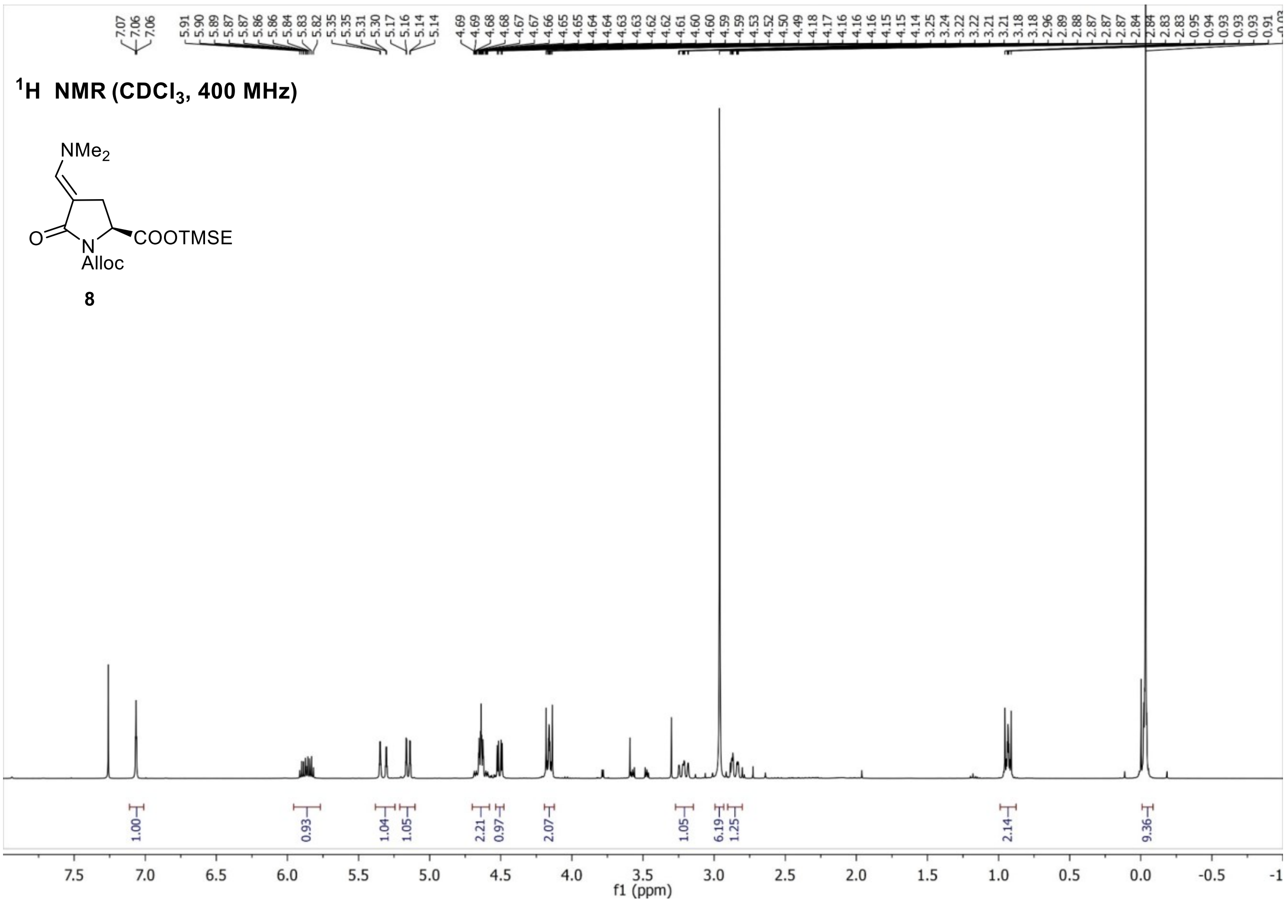
1H NMR (CDCl3, 400 MHz)
NMe2
O
N
COOTMSE
Alloc
8
1.00
0.93
1.04
1.05
2.21
0.97
2.07
1.05
6.19
1.25
2.14
9.36
f1 (ppm)

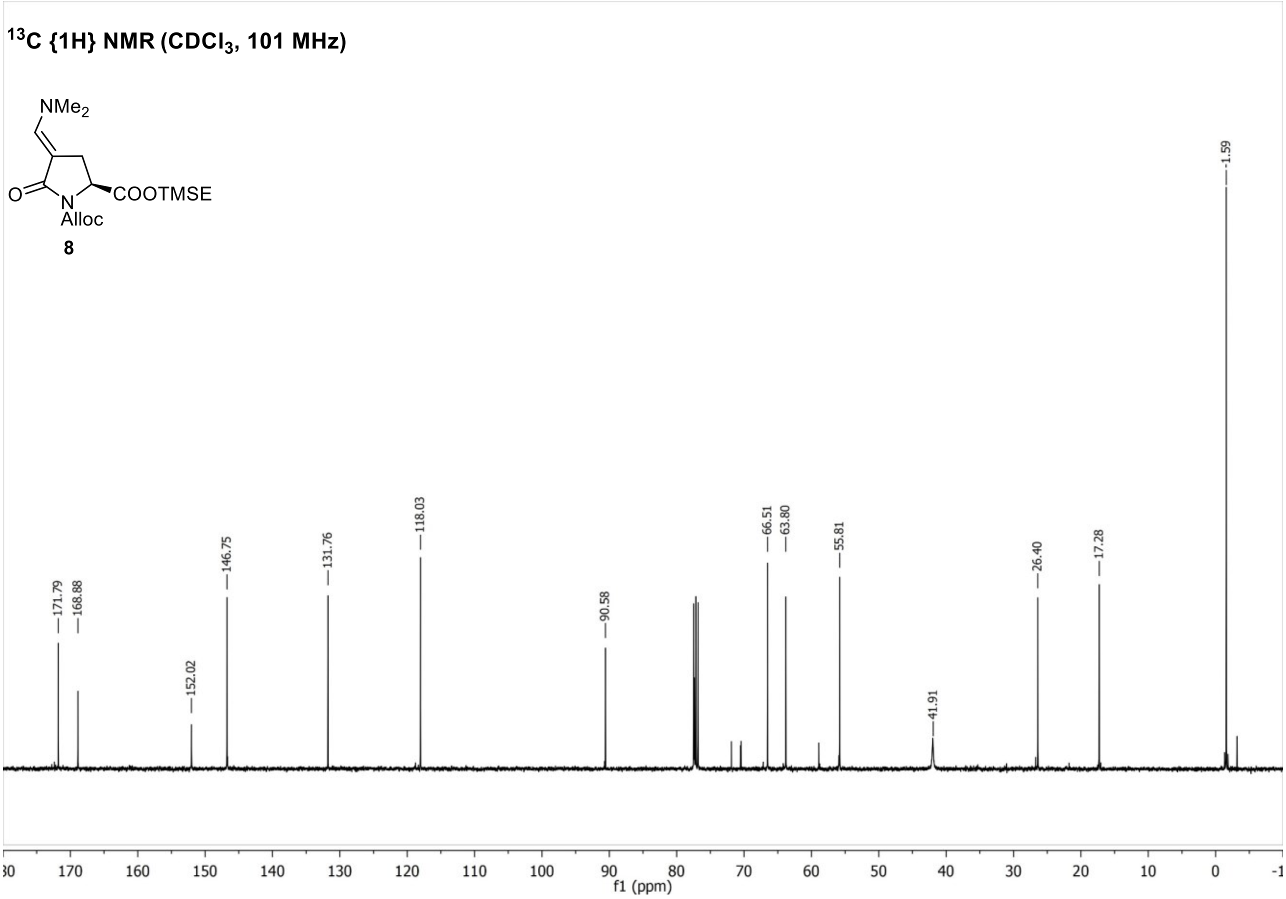
13C {1H} NMR (CDCl3, 101 MHz)
NMe2
O
N
COOTMSE
Alloc
8
171.79
168.88
152.02
146.75
131.76
118.03
90.58
66.51
63.80
55.81
41.91
26.40
17.28
-1.59
f1 (ppm)

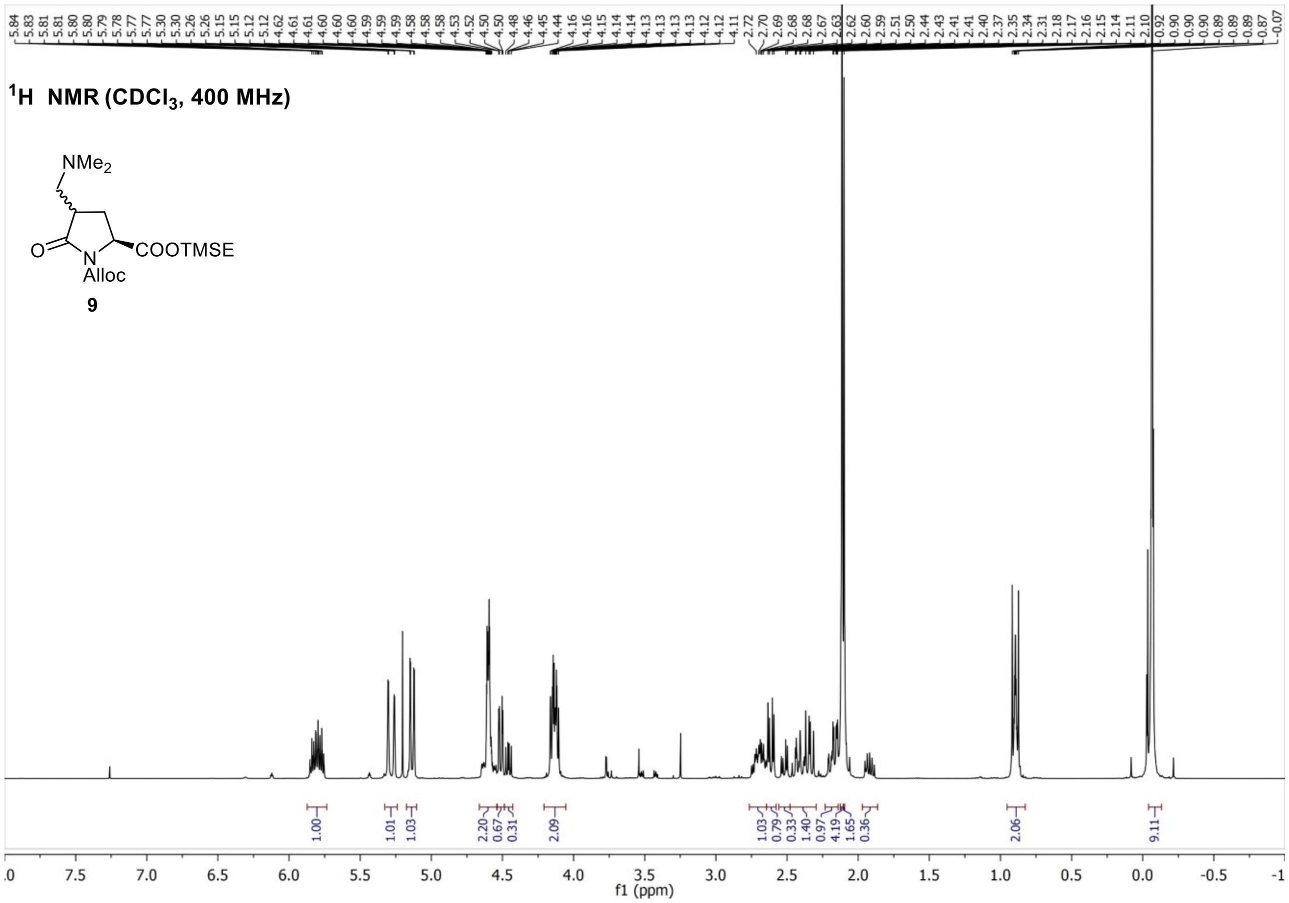

^{1}H NMR (CDCl$_3$, 400 MHz)
NMe$_2$
O
N
COOTMSE
Alloc
9
5.84 5.83 5.81 5.81 5.80 5.80 5.79 5.78 5.77 5.77 5.30 5.30 5.26 5.26 5.15 5.15 5.12 5.12 4.62 4.61 4.61 4.60 4.60 4.60 4.59 4.59 4.59 4.58 4.58 4.58 4.53 4.52 4.50 4.50 4.48 4.46 4.45 4.44 4.16 4.16 4.15 4.14 4.14 4.13 4.13 4.13 4.13 4.12 4.12 4.11 2.72 2.70 2.69 2.68 2.68 2.67 2.63 2.62 2.60 2.59 2.51 2.50 2.44 2.43 2.41 2.40 2.37 2.35 2.34 2.31 2.18 2.17 2.16 2.15 2.14 2.11 2.10 0.92 0.90 0.90 0.90 0.90 0.89 0.89 0.87 -0.07
1.00 1.01 1.03 2.20 0.67 0.31 2.09 1.03 0.79 0.33 1.40 0.97 4.19 1.65 0.36 2.06 9.11
f1 (ppm)

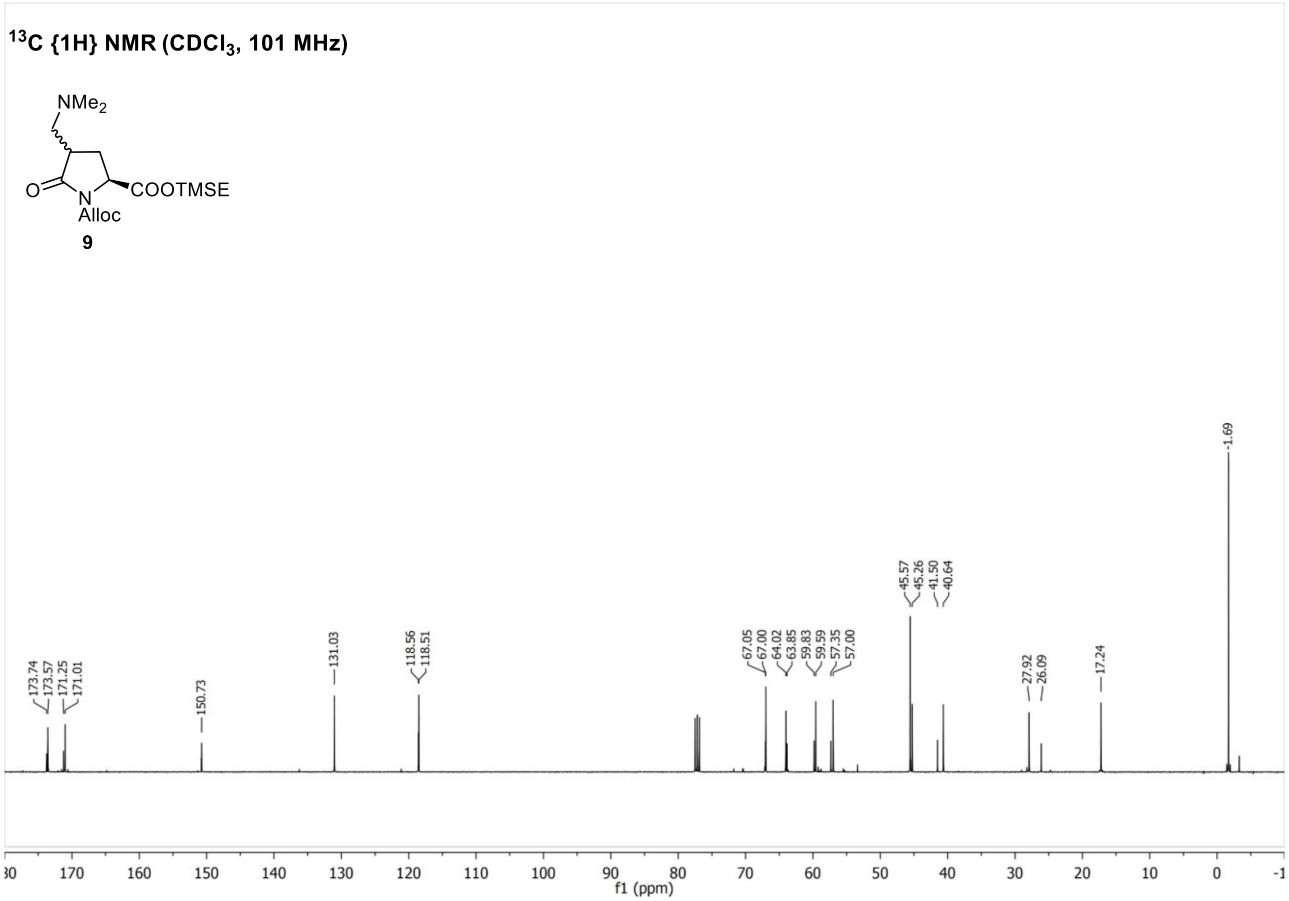

^{13}C {1H} NMR (CDCl$_3$, 101 MHz)
NMe$_2$
O
N
COOTMSE
Alloc
9
173.74 173.57 171.25 171.01
150.73
131.03
118.56 118.51
67.05 67.00 64.02 63.85 59.83 59.59 57.35 57.00
45.57 45.26 41.50 40.64
27.92 26.09
17.24
-1.69
f1 (ppm)

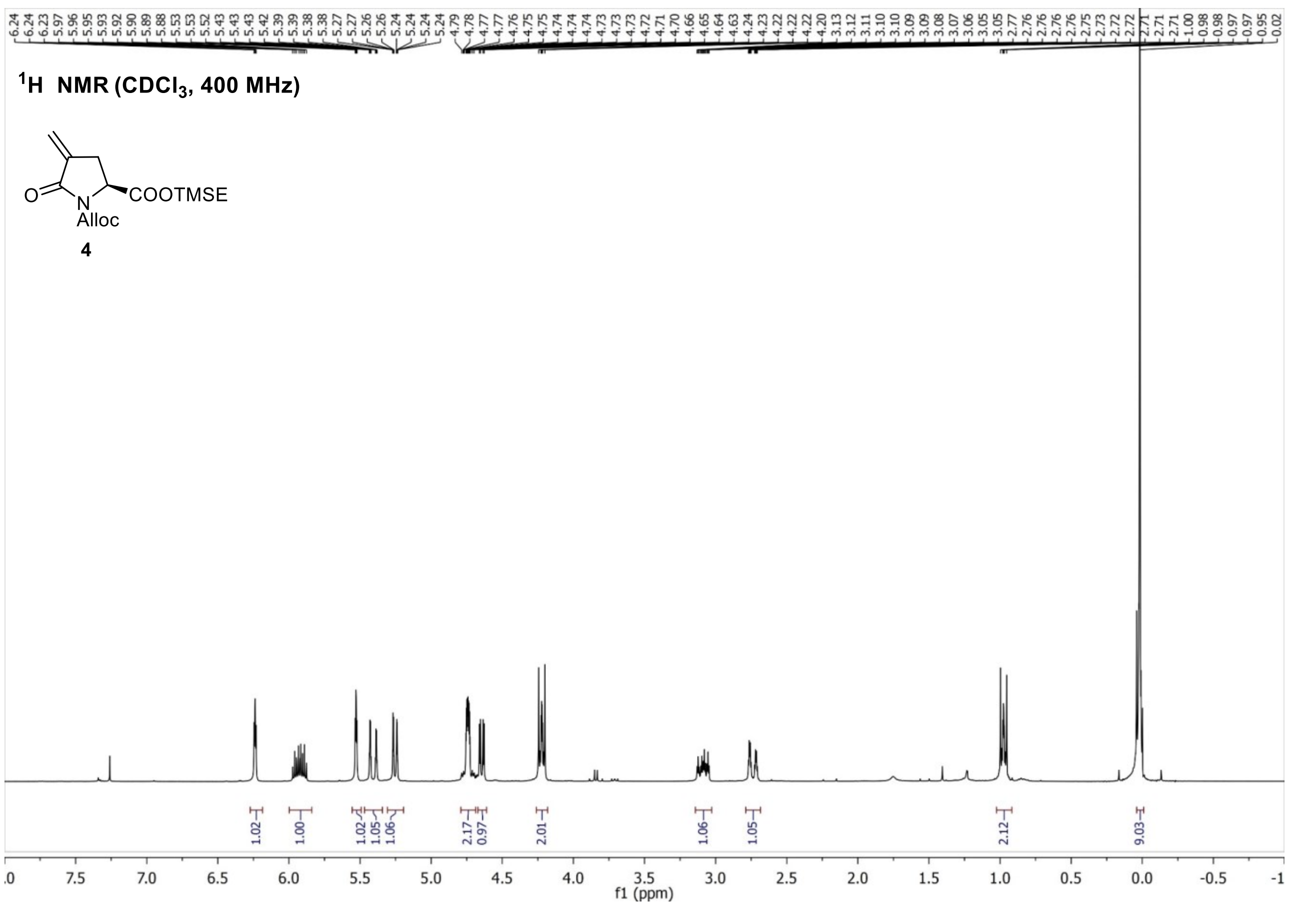

6.24 6.24 6.23 5.97 5.96 5.95 5.93 5.92 5.90 5.89 5.88 5.53 5.53 5.52 5.43 5.43 5.43 5.42 5.39 5.38 5.38 5.27 5.26 5.24 5.24 5.24 5.24 5.24 4.79 4.78 4.77 4.77 4.76 4.75 4.75 4.74 4.74 4.74 4.73 4.73 4.72 4.71 4.70 4.66 4.65 4.64 4.63 4.24 4.23 4.22 4.22 4.20 3.13 3.12 3.11 3.10 3.10 3.09 3.08 3.07 3.06 3.05 2.77 2.76 2.76 2.76 2.75 2.73 2.72 2.72 2.71 2.71 1.00 0.98 0.98 0.97 0.97 0.95 0.02
^{1}H NMR (CDCl$_3$, 400 MHz)
COOTMSE
O
N
Alloc
4
1.02 1.00 1.02 1.05 1.06 2.17 0.97 2.01 1.06 1.05 2.12 9.03
f1 (ppm)

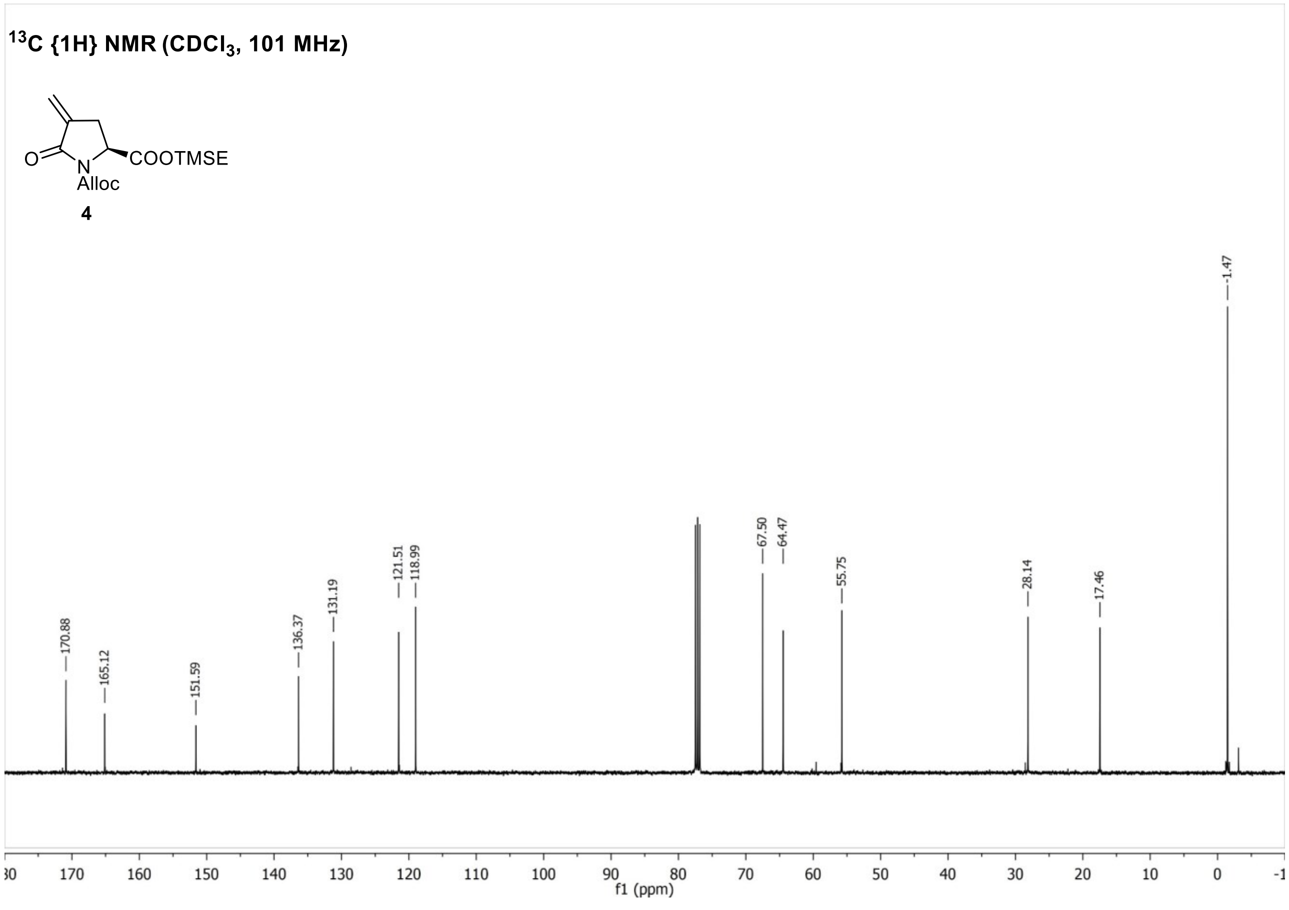

^{13}C {1H} NMR (CDCl$_3$, 101 MHz)
COOTMSE
O
N
Alloc
4
170.88 165.12 151.59 136.37 131.19 121.51 118.99 67.50 64.47 55.75 28.14 17.46 -1.47
f1 (ppm)

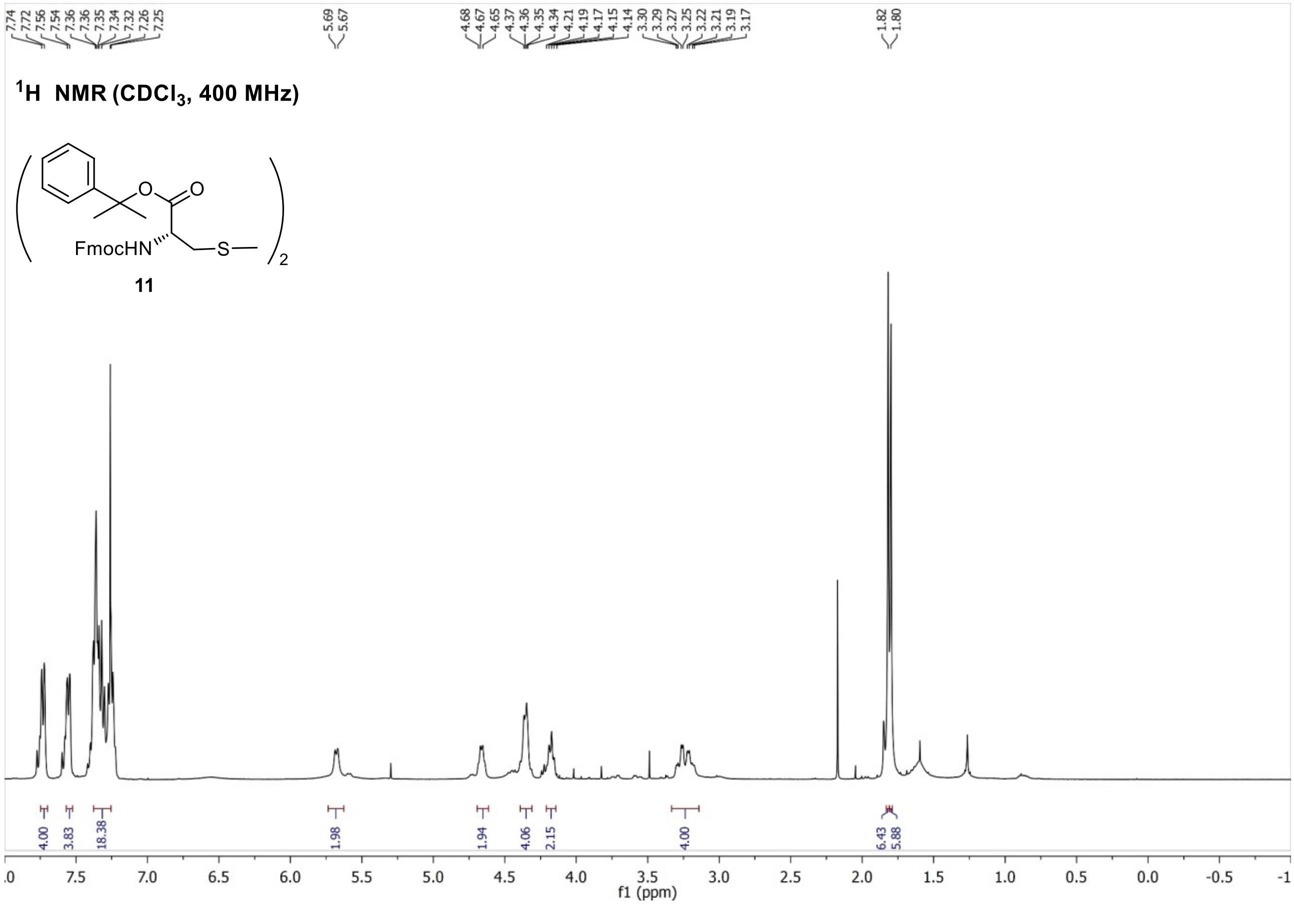
7.74
7.72
7.56
7.54
7.36
7.36
7.35
7.34
7.32
7.26
7.25
5.69
5.67
4.68
4.67
4.65
4.37
4.36
4.35
4.34
4.21
4.19
4.17
4.15
4.14
3.30
3.29
3.27
3.25
3.22
3.21
3.19
3.17
1.82
1.80
^{1}H NMR ($CDCl_3$, 400 MHz)
O
O
FmocHN
S
2
11
4.00
3.83
18.38
1.98
1.94
4.06
2.15
4.00
6.43
5.88
7.5
7.0
6.5
6.0
5.5
5.0
4.5
4.0
3.5
3.0
2.5
2.0
1.5
1.0
0.5
0.0
-0.5
f1 (ppm)

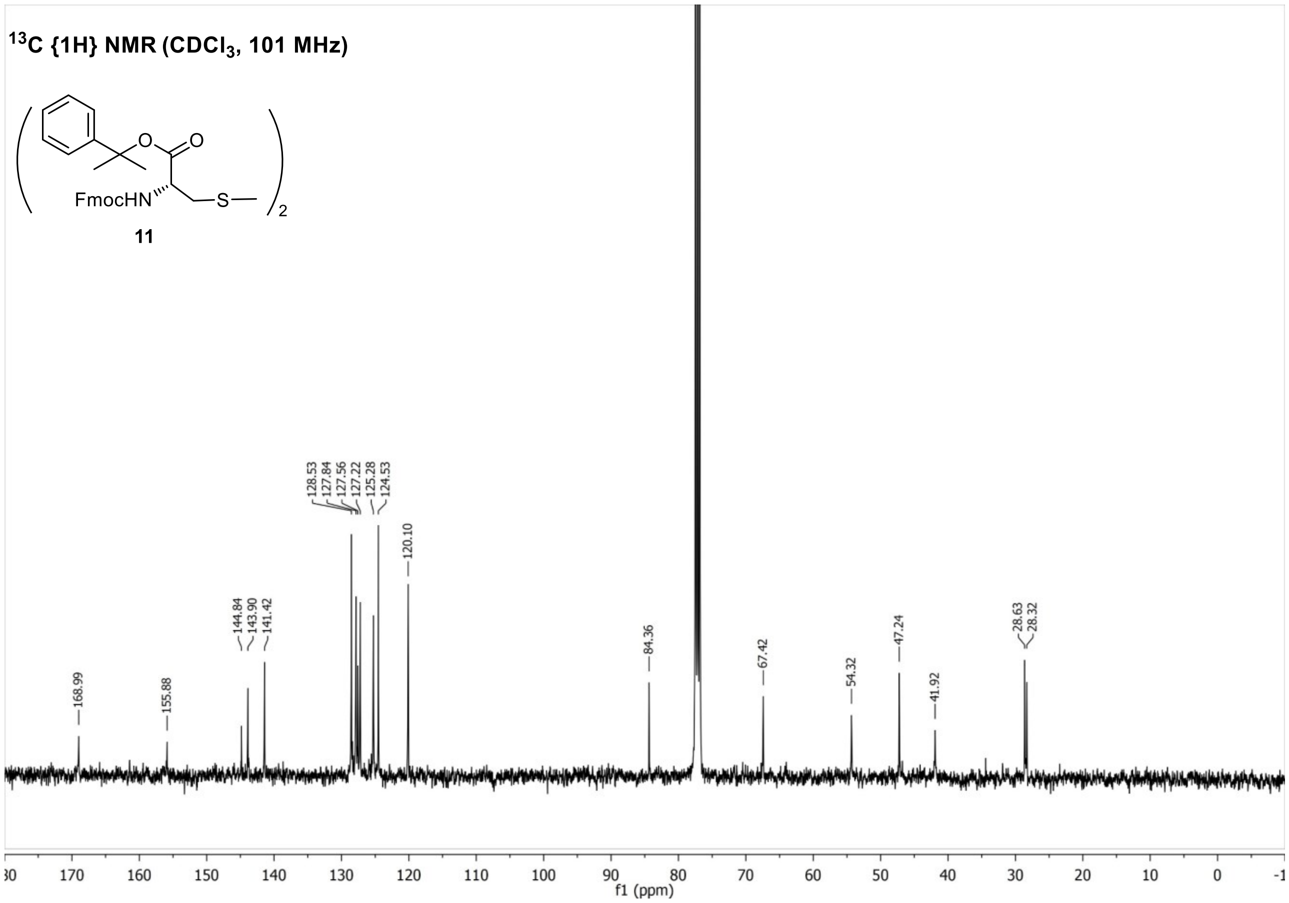
^{13}C {1H} NMR ($CDCl_3$, 101 MHz)
O
O
FmocHN
S
2
11
128.53
127.84
127.56
127.22
125.28
124.53
120.10
144.84
143.90
141.42
168.99
155.88
84.36
67.42
54.32
47.24
41.92
28.63
28.32
170
160
150
140
130
120
110
100
90
80
70
60
50
40
30
20
10
0
f1 (ppm)

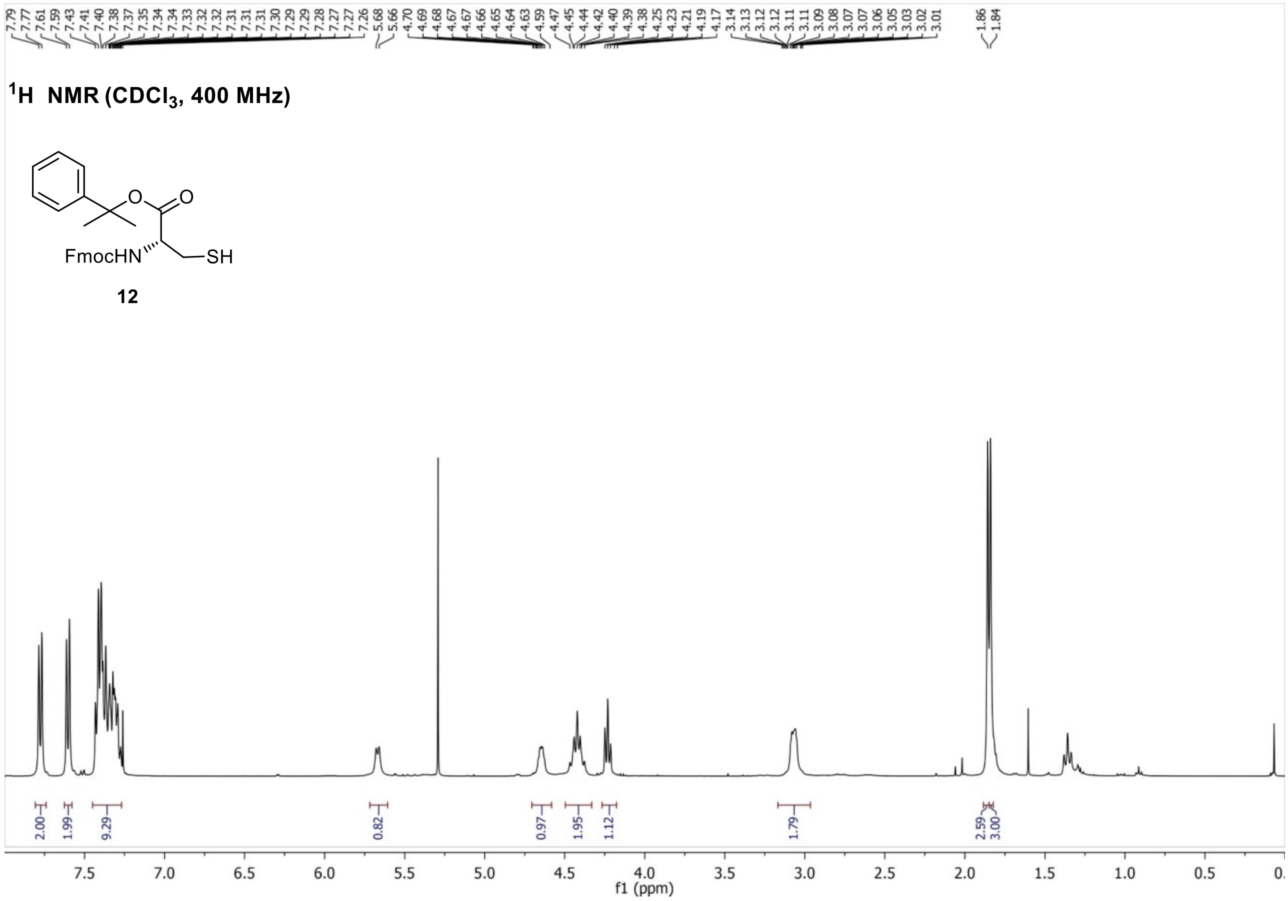

1H NMR (CDCl3, 400 MHz)
FmocHN
SH
12
f1 (ppm)

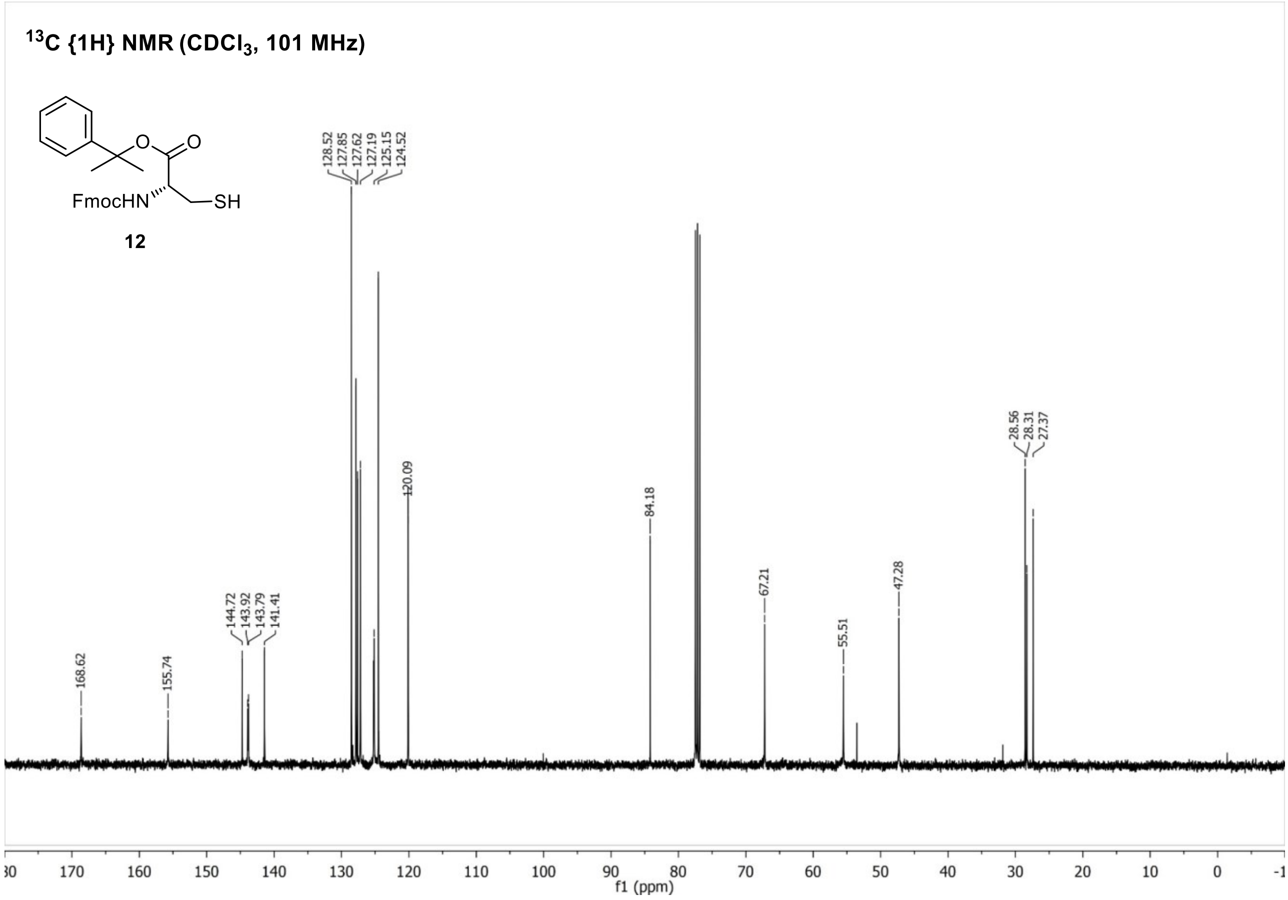

13C {1H} NMR (CDCl3, 101 MHz)
FmocHN
SH
12
f1 (ppm)

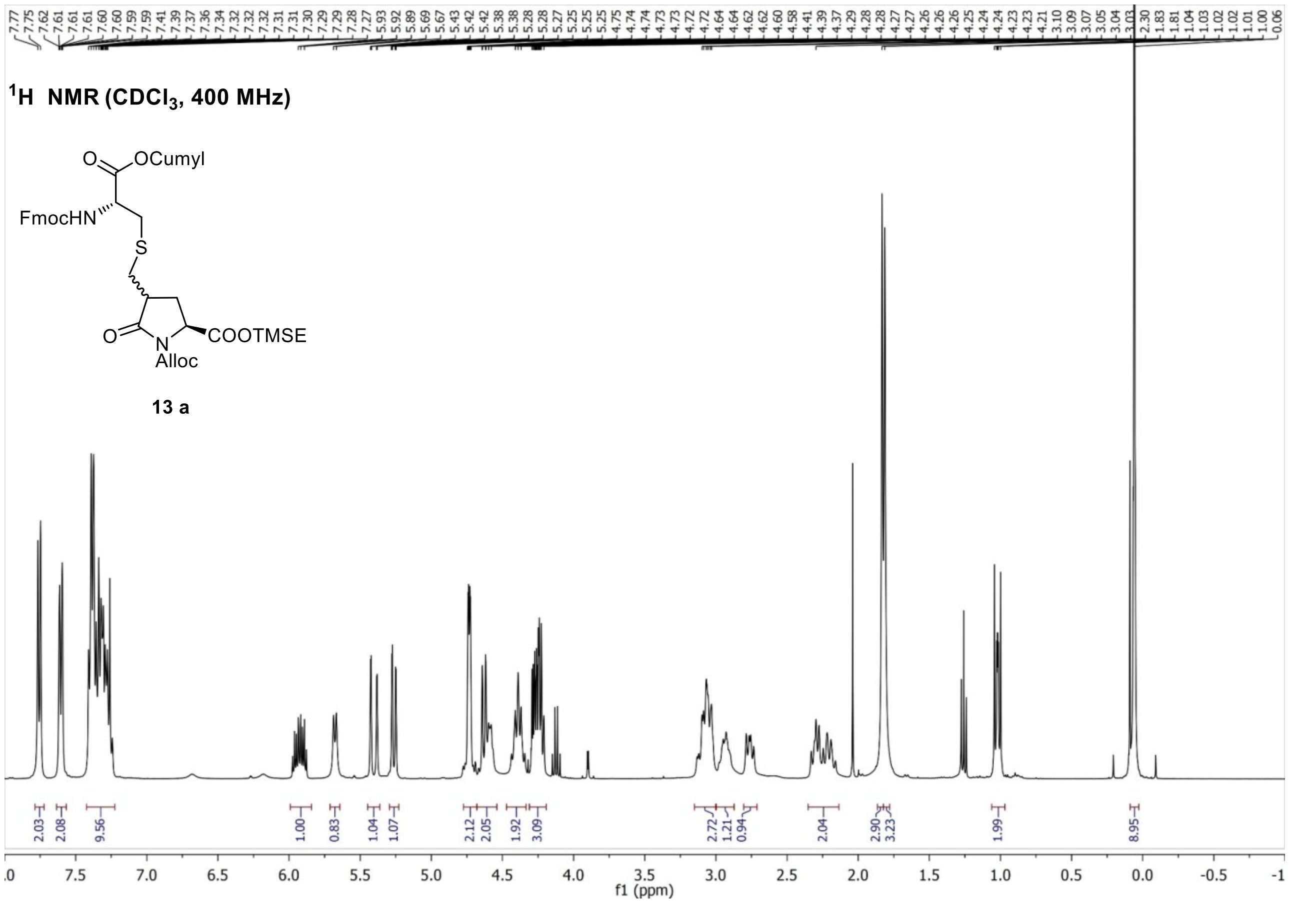

1H NMR (CDCl3, 400 MHz)
OCumyl
FmocHN
S
COOTMSE
N
Alloc
13 a
f1 (ppm)

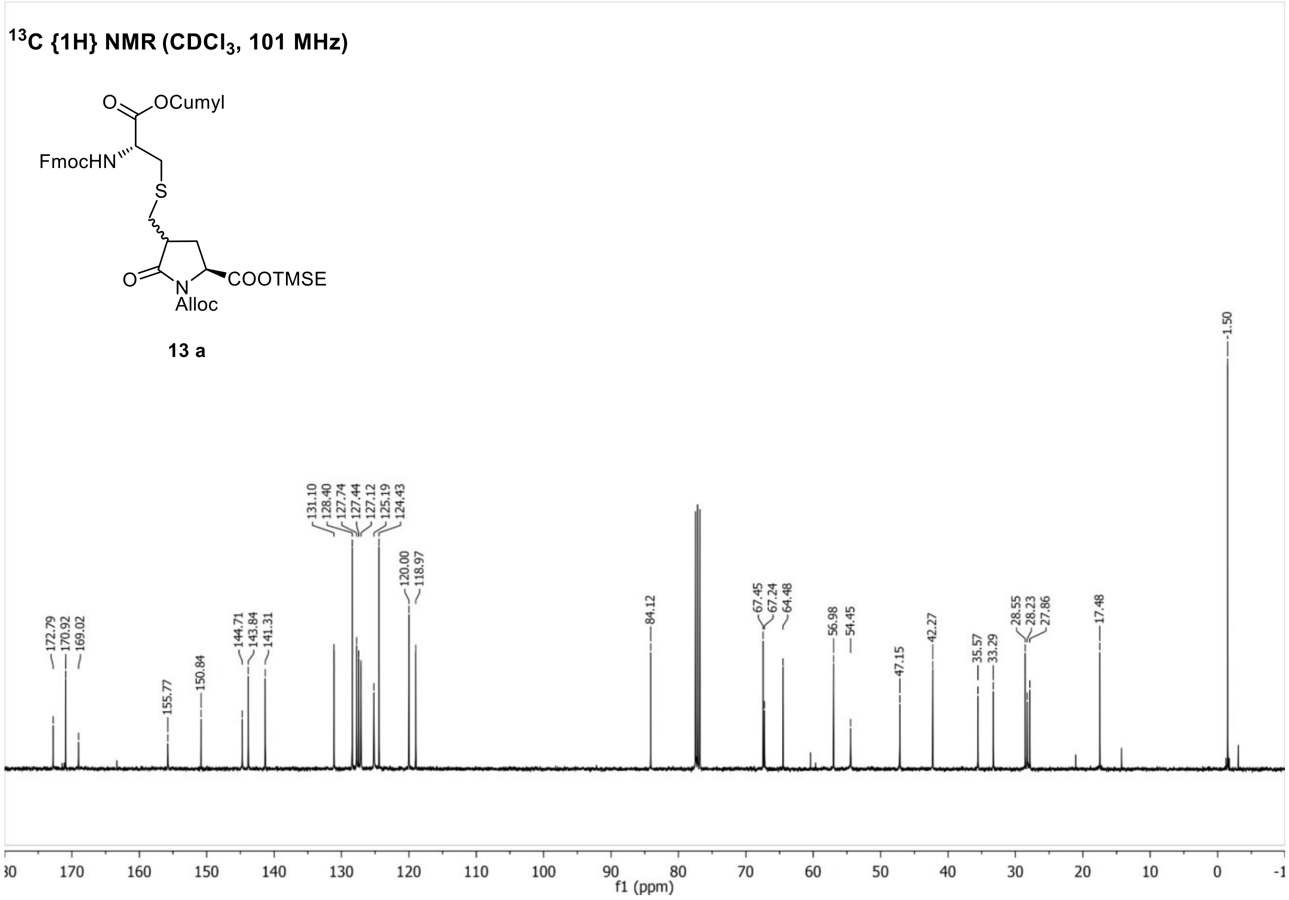

13C {1H} NMR (CDCl3, 101 MHz)
OCumyl
FmocHN
S
COOTMSE
N
Alloc
13 a
172.79
170.92
169.02
155.77
150.84
144.71
143.84
141.31
131.10
128.40
127.74
127.44
127.12
125.19
124.43
120.00
118.97
84.12
67.45
67.24
64.48
56.98
54.45
47.15
42.27
35.57
33.29
28.55
28.23
27.86
17.48
-1.50
f1 (ppm)

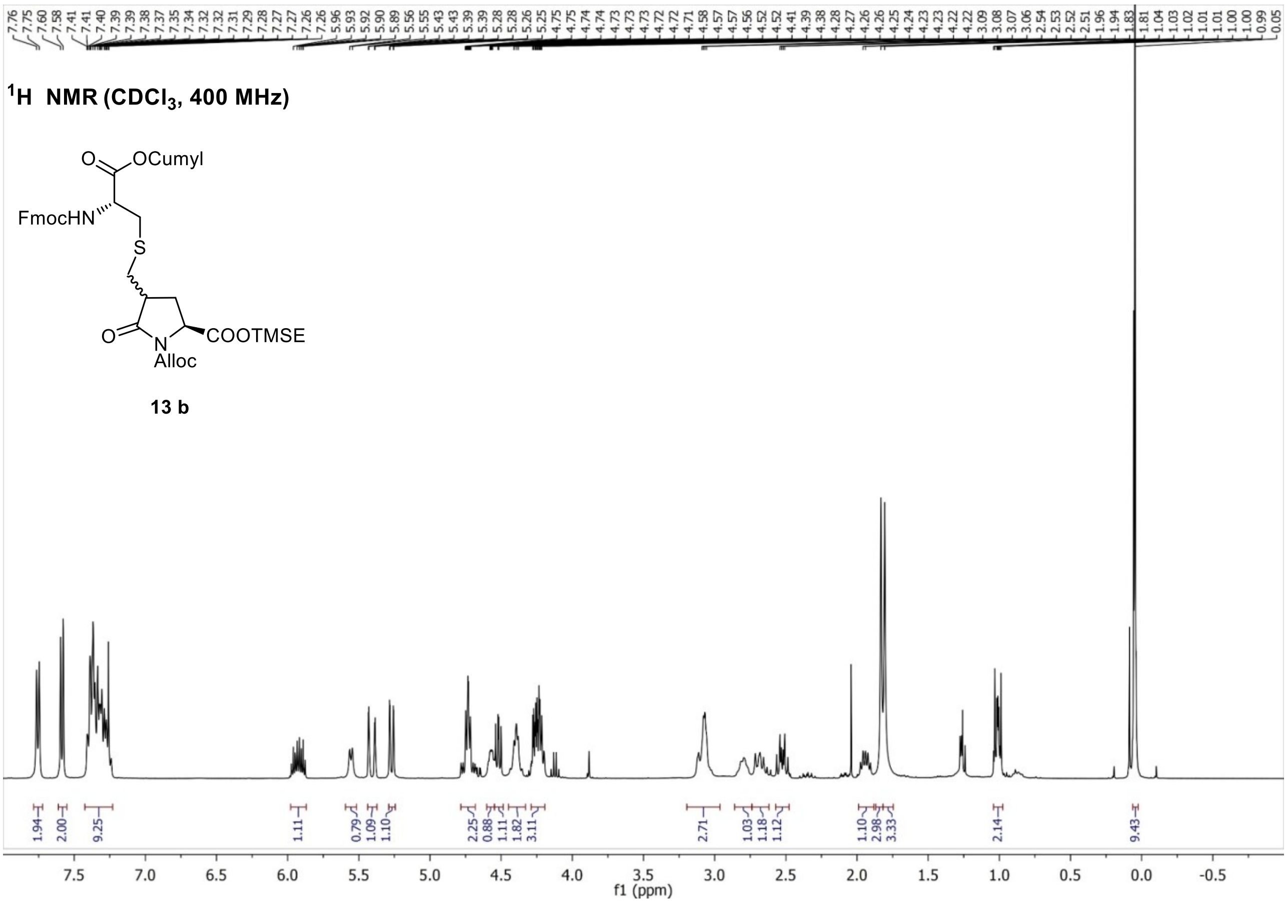
7.76 7.75 7.60 7.58 7.41 7.41 7.40 7.39 7.39 7.38 7.37 7.35 7.34 7.32 7.32 7.31 7.29 7.28 7.27 7.27 7.26 7.26 5.96 5.93 5.92 5.90 5.89 5.56 5.55 5.43 5.39 5.39 5.28 5.28 5.26 5.25 4.75 4.75 4.74 4.74 4.73 4.73 4.73 4.72 4.72 4.71 4.58 4.57 4.57 4.56 4.52 4.52 4.41 4.39 4.38 4.28 4.27 4.26 4.26 4.25 4.24 4.23 4.23 4.22 4.22 3.09 3.08 3.07 3.06 2.54 2.53 2.52 2.52 2.51 1.96 1.94 1.83 1.81 1.04 1.03 1.02 1.01 1.01 1.00 1.00 0.99 0.05
1H NMR (CDCl3, 400 MHz)
O
OCumyl
FmocHN
S
O
N
COOTMSE
Alloc
13 b
1.94 2.00 9.25 1.11 0.79 1.09 1.10 2.25 0.88 1.11 1.82 3.11 2.71 1.03 1.18 1.12 1.10 2.98 3.33 2.14 9.43
7.5 7.0 6.5 6.0 5.5 5.0 4.5 4.0 3.5 3.0 2.5 2.0 1.5 1.0 0.5 0.0 -0.5
f1 (ppm)

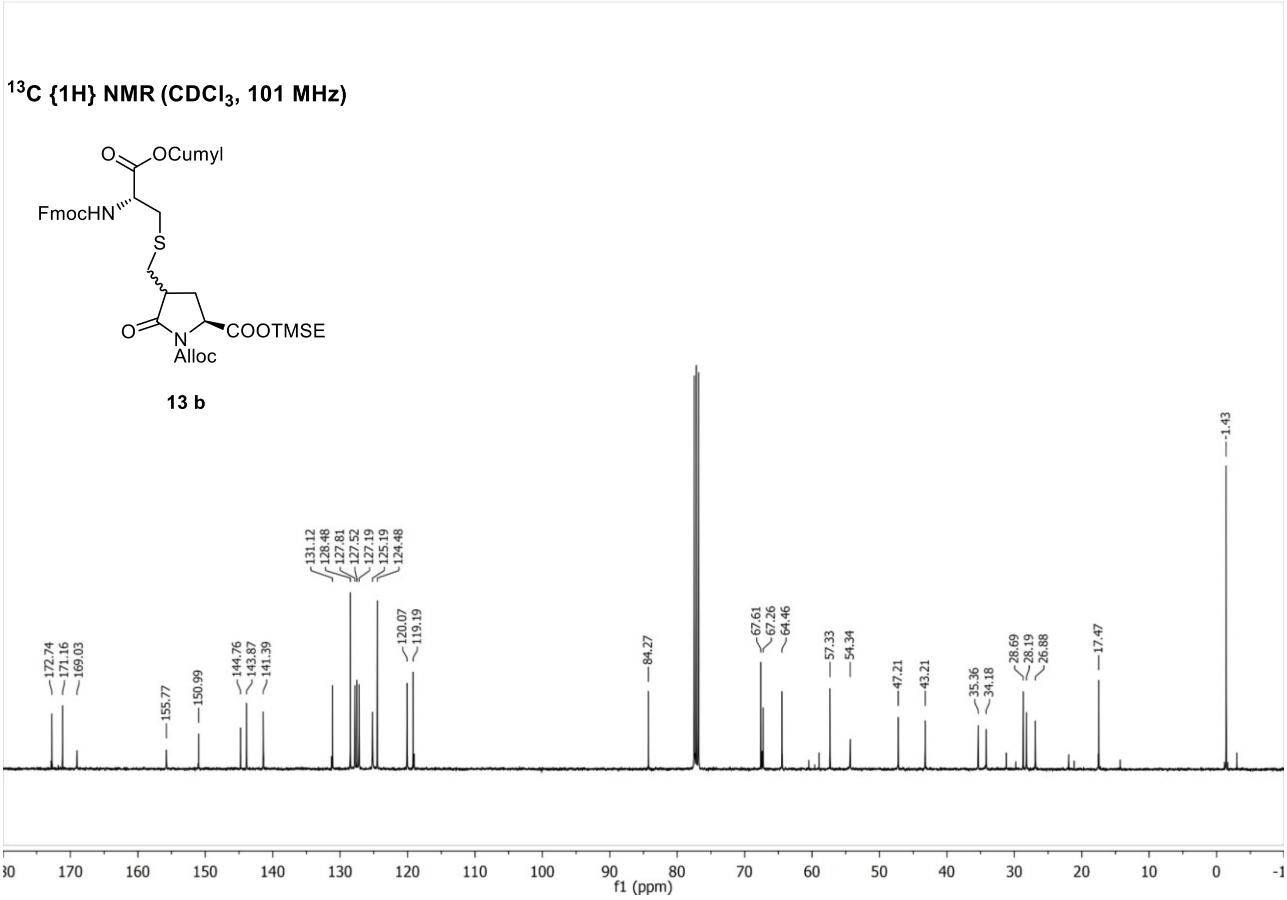
13C {1H} NMR (CDCl3, 101 MHz)
O
OCumyl
FmocHN
S
O
N
COOTMSE
Alloc
13 b
172.74 171.16 169.03 155.77 150.99 144.76 143.87 141.39 131.12 128.48 127.81 127.52 127.19 125.19 124.48 120.07 119.19 84.27 67.61 67.26 64.46 57.33 54.34 47.21 43.21 35.36 34.18 28.69 28.19 26.88 17.47 -1.43
170 160 150 140 130 120 110 100 90 80 70 60 50 40 30 20 10 0
f1 (ppm)

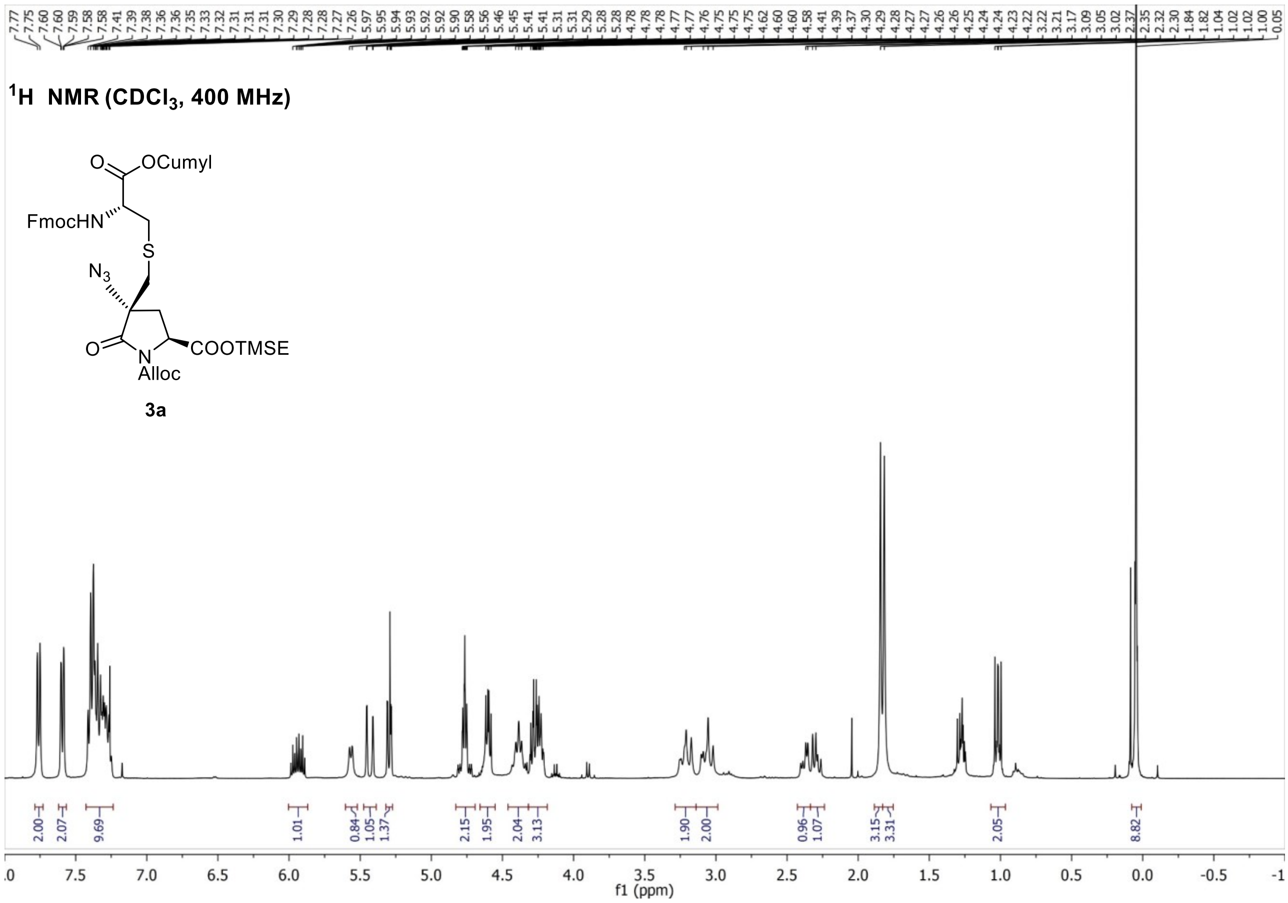
7.77
7.75
7.60
7.60
7.59
7.58
7.58
7.41
7.39
7.38
7.36
7.36
7.35
7.33
7.32
7.31
7.31
7.31
7.30
7.29
7.28
7.28
7.27
7.26
5.97
5.95
5.94
5.93
5.92
5.92
5.90
5.58
5.56
5.46
5.45
5.41
5.41
5.31
5.31
5.29
5.28
4.78
4.78
4.78
4.77
4.77
4.76
4.75
4.75
4.62
4.60
4.60
4.58
4.41
4.39
4.37
4.30
4.29
4.28
4.27
4.27
4.26
4.26
4.25
4.24
4.24
4.23
4.22
3.22
3.21
3.17
3.09
3.05
3.02
2.37
2.35
2.32
2.30
1.84
1.82
1.04
1.02
1.02
1.00
0.05
¹H NMR (CDCl₃, 400 MHz)
O
OCumyl
FmocHN
S
N₃
O
N
COOTMSE
Alloc
3a
2.00
2.07
9.69
1.01
0.84
1.05
1.37
2.15
1.95
2.04
3.13
1.90
2.00
0.96
1.07
3.15
3.31
2.05
8.82
f1 (ppm)

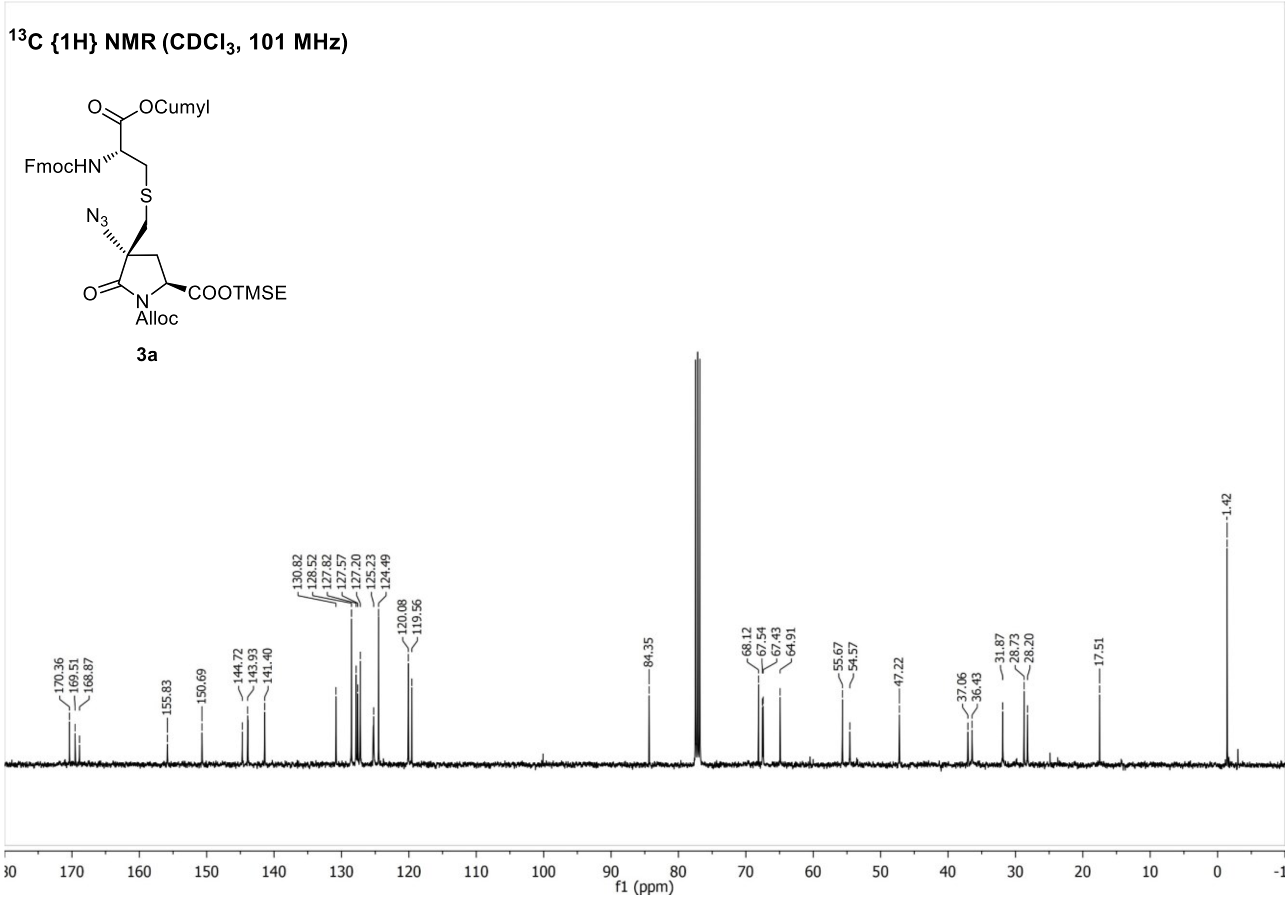
¹³C {1H} NMR (CDCl₃, 101 MHz)
O
OCumyl
FmocHN
S
N₃
O
N
COOTMSE
Alloc
3a
170.36
169.51
168.87
155.83
150.69
144.72
143.93
141.40
130.82
128.52
127.82
127.57
127.20
125.23
124.49
120.08
119.56
84.35
68.12
67.54
67.43
64.91
55.67
54.57
47.22
37.06
36.43
31.87
28.73
28.20
17.51
-1.42
f1 (ppm)

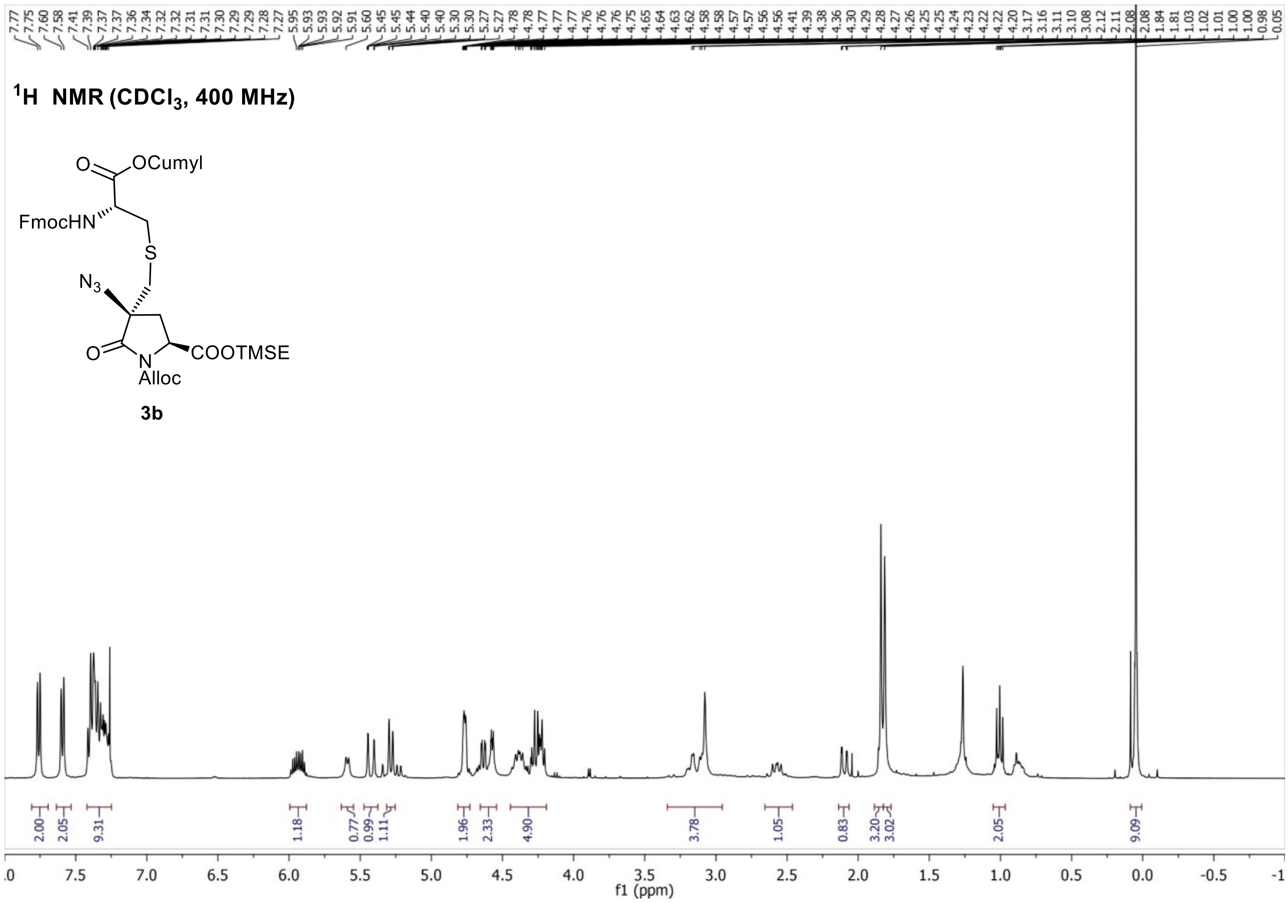

7.77 7.75 7.60 7.58 7.41 7.39 7.37 7.37 7.36 7.34 7.32 7.32 7.31 7.31 7.30 7.29 7.29 7.28 7.27 5.95 5.93 5.93 5.92 5.91 5.60 5.45 5.45 5.44 5.40 5.40 5.30 5.30 5.27 5.27 4.78 4.78 4.77 4.77 4.77 4.76 4.76 4.75 4.65 4.64 4.63 4.62 4.58 4.58 4.57 4.57 4.56 4.56 4.41 4.39 4.38 4.36 4.30 4.29 4.28 4.27 4.26 4.25 4.25 4.24 4.23 4.22 4.22 4.20 3.17 3.16 3.11 3.10 3.08 2.12 2.11 2.08 2.08 1.84 1.81 1.03 1.02 1.01 1.00 1.00 0.98 0.05
1H NMR (CDCl3, 400 MHz)
O
OCumyl
FmocHN
S
N3
O
N
COOTMSE
Alloc
3b
2.00 2.05 9.31 1.18 0.77 0.99 1.11 1.96 2.33 4.90 3.78 1.05 0.83 3.20 3.02 2.05 9.09
f1 (ppm)

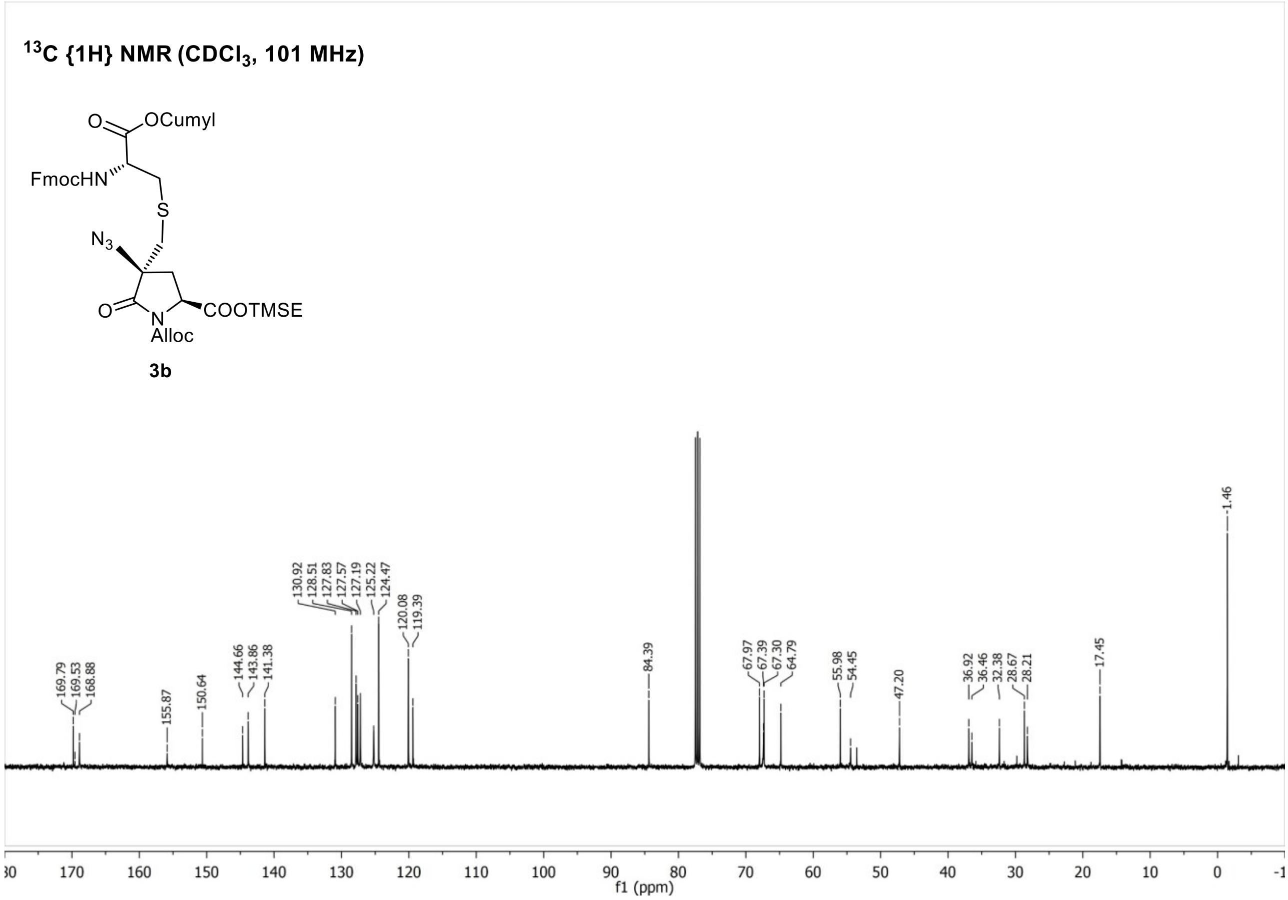

13C {1H} NMR (CDCl3, 101 MHz)
O
OCumyl
FmocHN
S
N3
O
N
COOTMSE
Alloc
3b
169.79 169.53 168.88 155.87 150.64 144.66 143.86 141.38 130.92 128.51 127.83 127.57 127.19 125.22 124.47 120.08 119.39 84.39 67.97 67.39 67.30 64.79 55.98 54.45 47.20 36.92 36.46 32.38 28.67 28.21 17.45 -1.46
f1 (ppm)

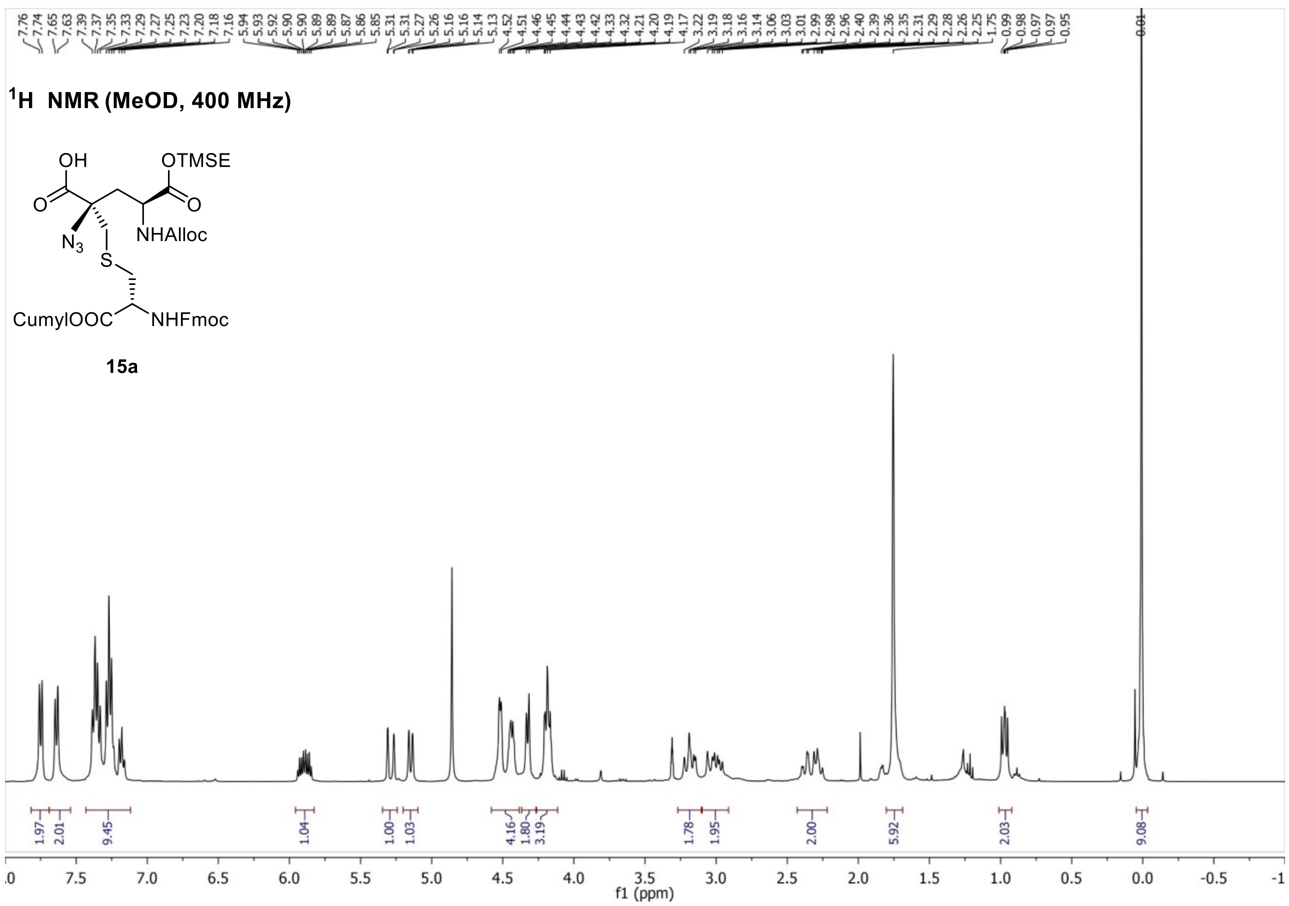
1H NMR (MeOD, 400 MHz)
OH
OTMSE
O
O
N3
NHAlloc
S
CumylOOC
NHFmoc
15a
f1 (ppm)

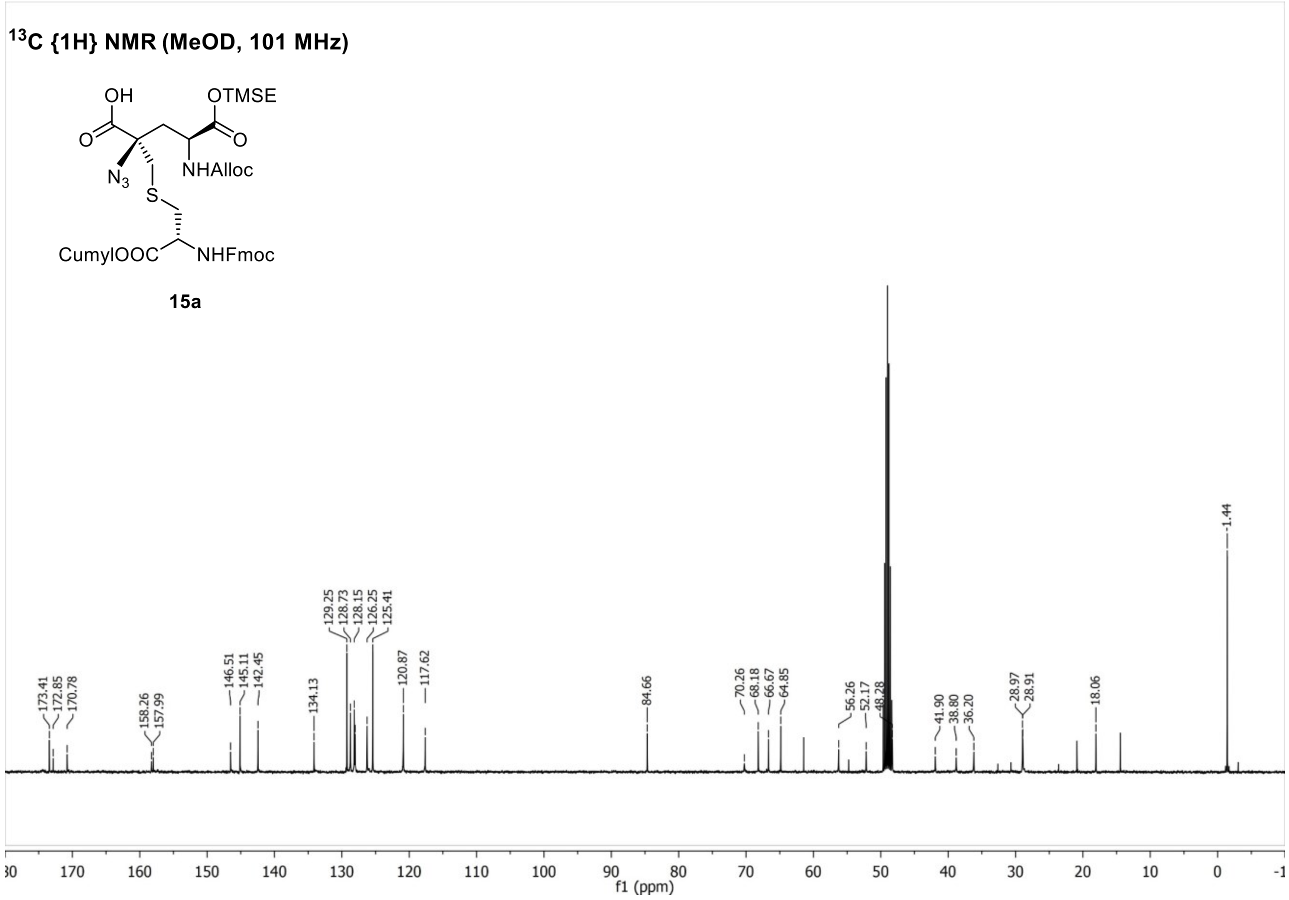
13C {1H} NMR (MeOD, 101 MHz)
OH
OTMSE
O
O
N3
NHAlloc
S
CumylOOC
NHFmoc
15a
f1 (ppm)

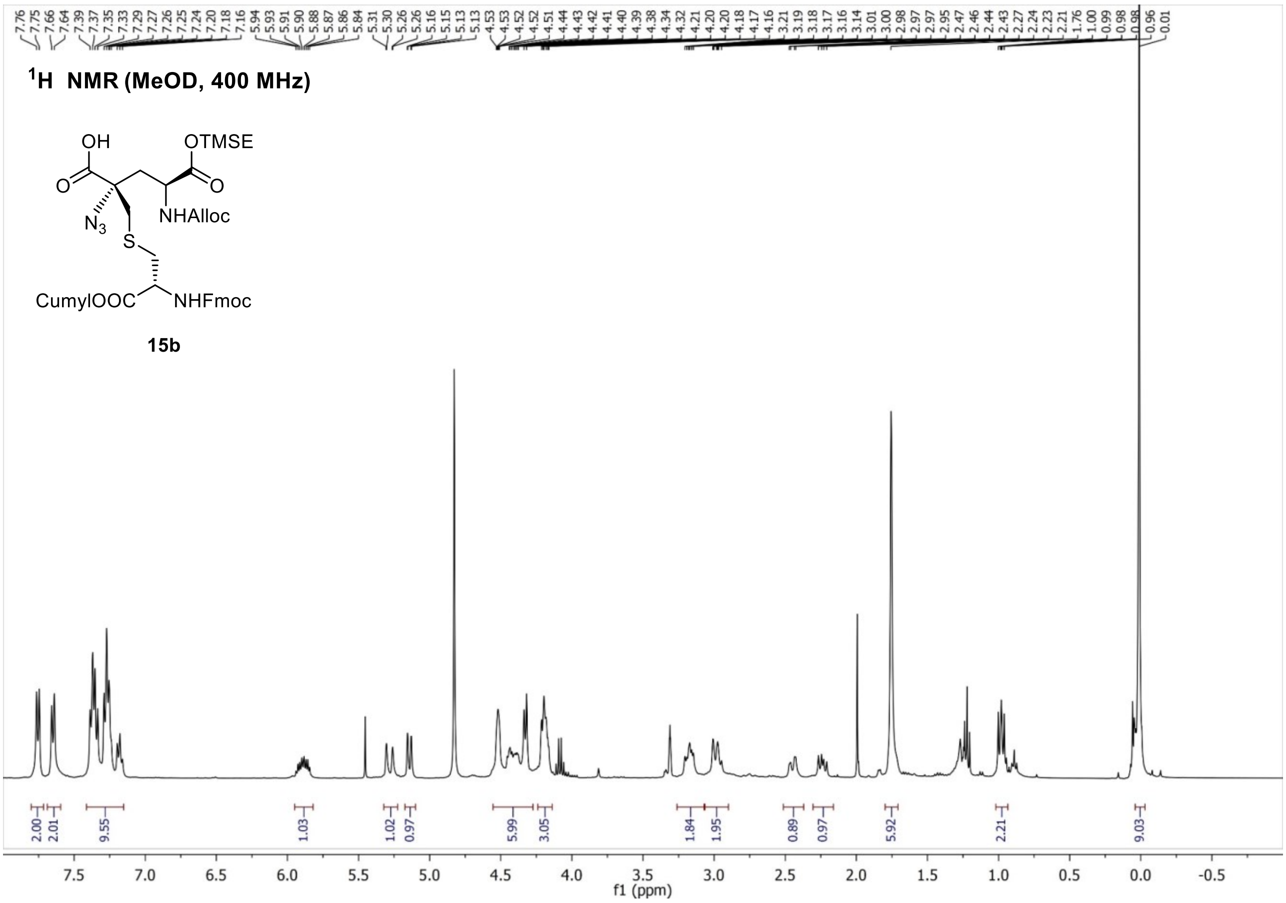

1H NMR (MeOD, 400 MHz)
OH
OTMSE
O
O
N3
NHAlloc
S
CumylOOC
NHFmoc
15b
7.76 7.75 7.66 7.64 7.39 7.37 7.35 7.33 7.29 7.27 7.26 7.25 7.24 7.20 7.18 7.16 5.94 5.93 5.91 5.90 5.88 5.87 5.86 5.84 5.31 5.30 5.26 5.26 5.16 5.15 5.13 5.13 4.53 4.53 4.52 4.52 4.51 4.44 4.43 4.42 4.41 4.40 4.39 4.38 4.34 4.32 4.21 4.20 4.20 4.18 4.17 4.16 3.21 3.19 3.18 3.17 3.16 3.14 3.01 3.00 2.98 2.97 2.97 2.95 2.47 2.46 2.44 2.43 2.27 2.24 2.23 2.21 1.76 1.00 0.99 0.98 0.98 0.96 0.01
2.00 2.01 9.55 1.03 1.02 0.97 5.99 3.05 1.84 1.95 0.89 0.97 5.92 2.21 9.03
7.5 7.0 6.5 6.0 5.5 5.0 4.5 4.0 3.5 3.0 2.5 2.0 1.5 1.0 0.5 0.0 -0.5
f1 (ppm)

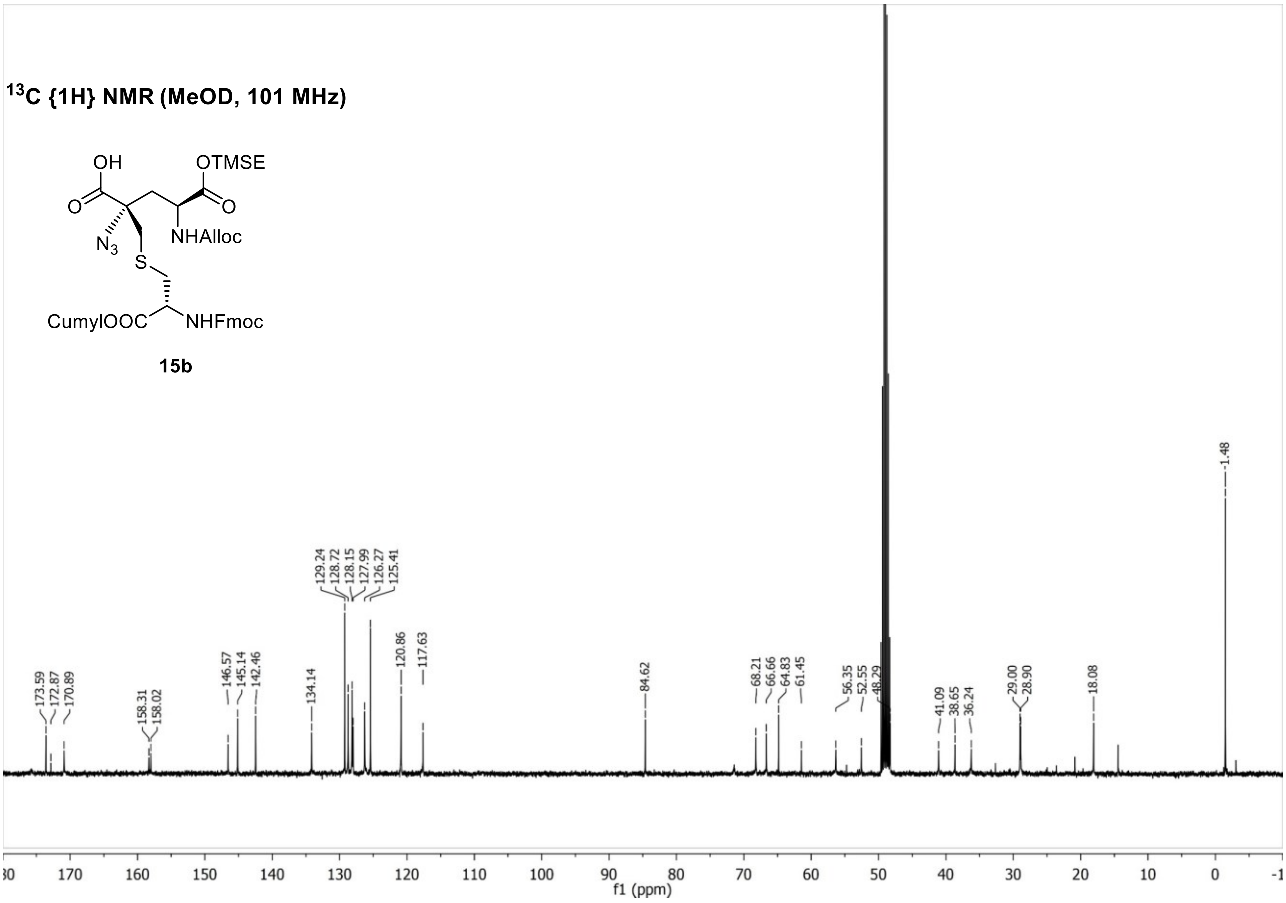

13C {1H} NMR (MeOD, 101 MHz)
OH
OTMSE
O
O
N3
NHAlloc
S
CumylOOC
NHFmoc
15b
173.59 172.87 170.89 158.31 158.02 146.57 145.14 142.46 134.14 129.24 128.72 128.15 127.99 126.27 125.41 120.86 117.63 84.62 68.21 66.66 64.83 61.45 56.35 52.55 48.29 41.09 38.65 36.24 29.00 28.90 18.08 -1.48
170 160 150 140 130 120 110 100 90 80 70 60 50 40 30 20 10 0
f1 (ppm)

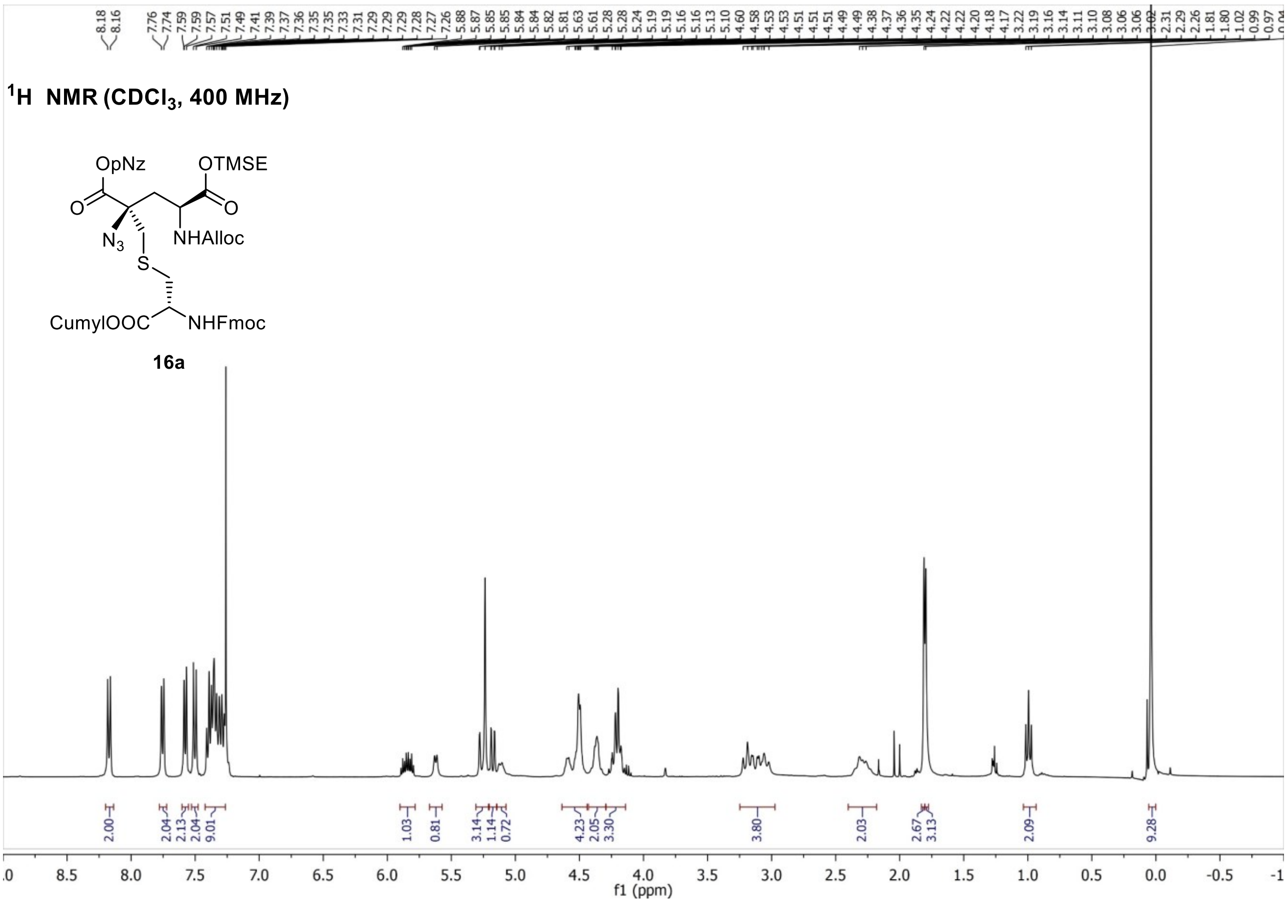
^{1}H NMR ($CDCl_3$, 400 MHz)
OpNz
OTMSE
N_3
NHAlloc
S
CumylOOC
NHFmoc
16a
f1 (ppm)

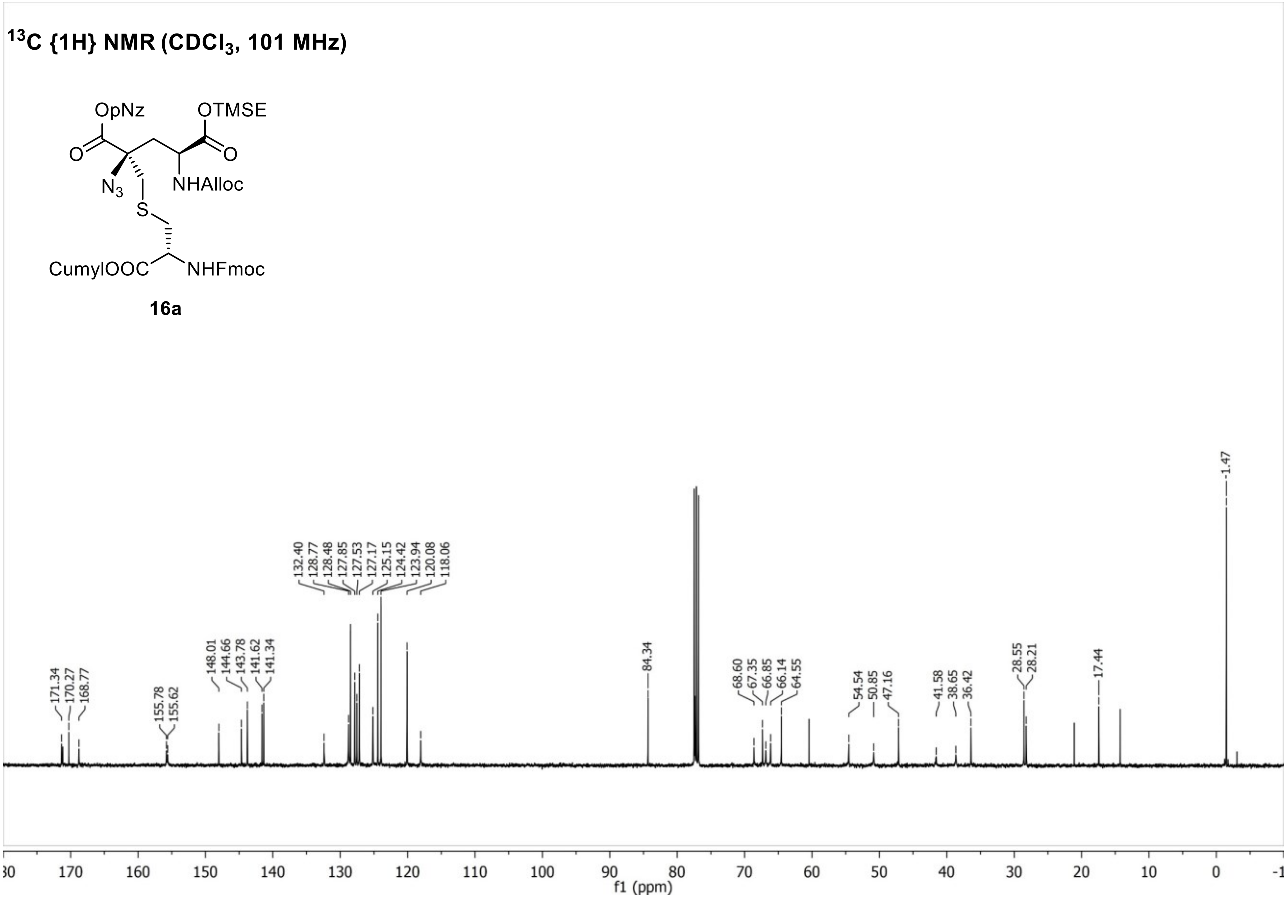
^{13}C {1H} NMR ($CDCl_3$, 101 MHz)
OpNz
OTMSE
N_3
NHAlloc
S
CumylOOC
NHFmoc
16a
f1 (ppm)

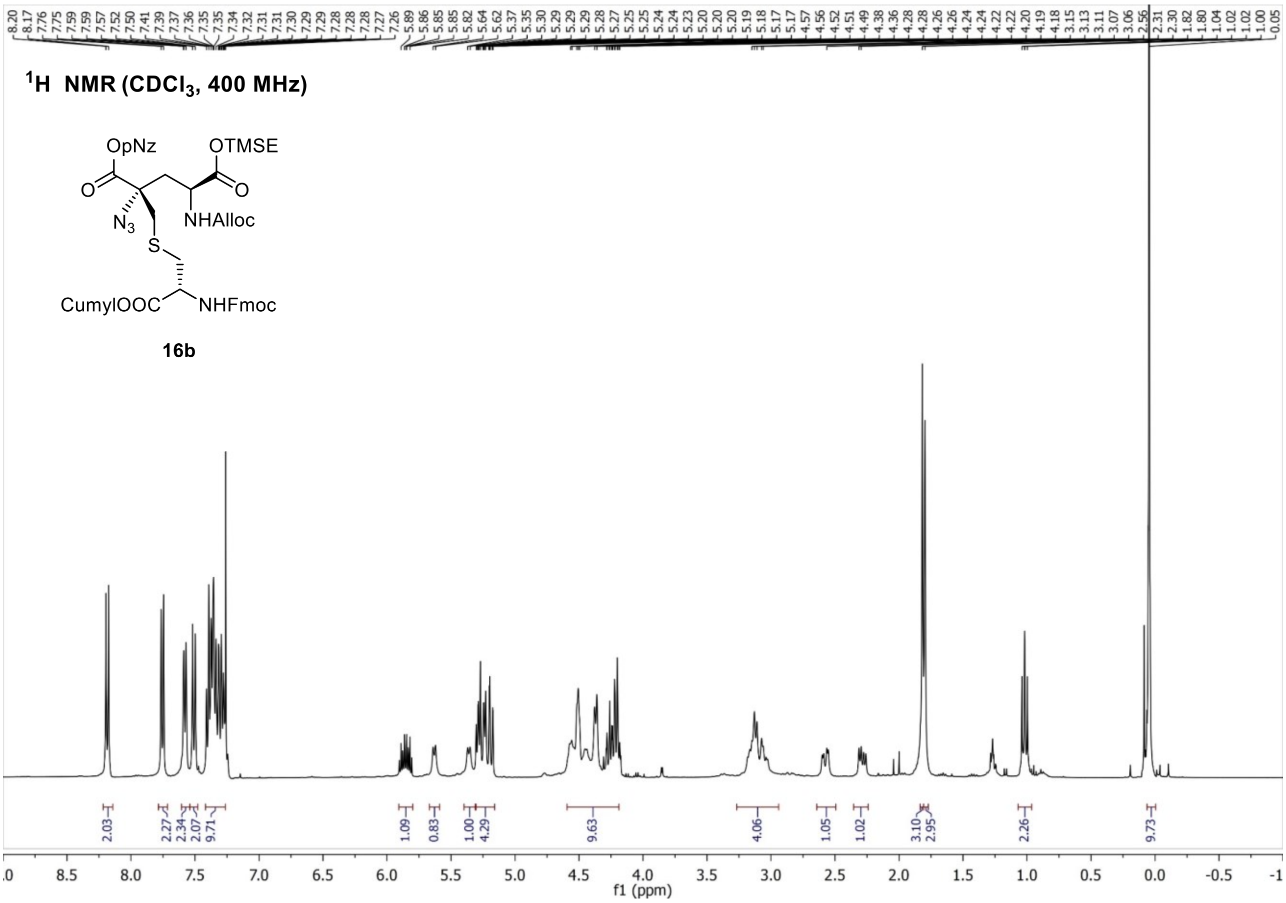
^{1}H NMR (CDCl$_3$, 400 MHz)
OpNz
OTMSE
N$_3$
NHAlloc
S
CumylOOC
NHFmoc
16b
f1 (ppm)

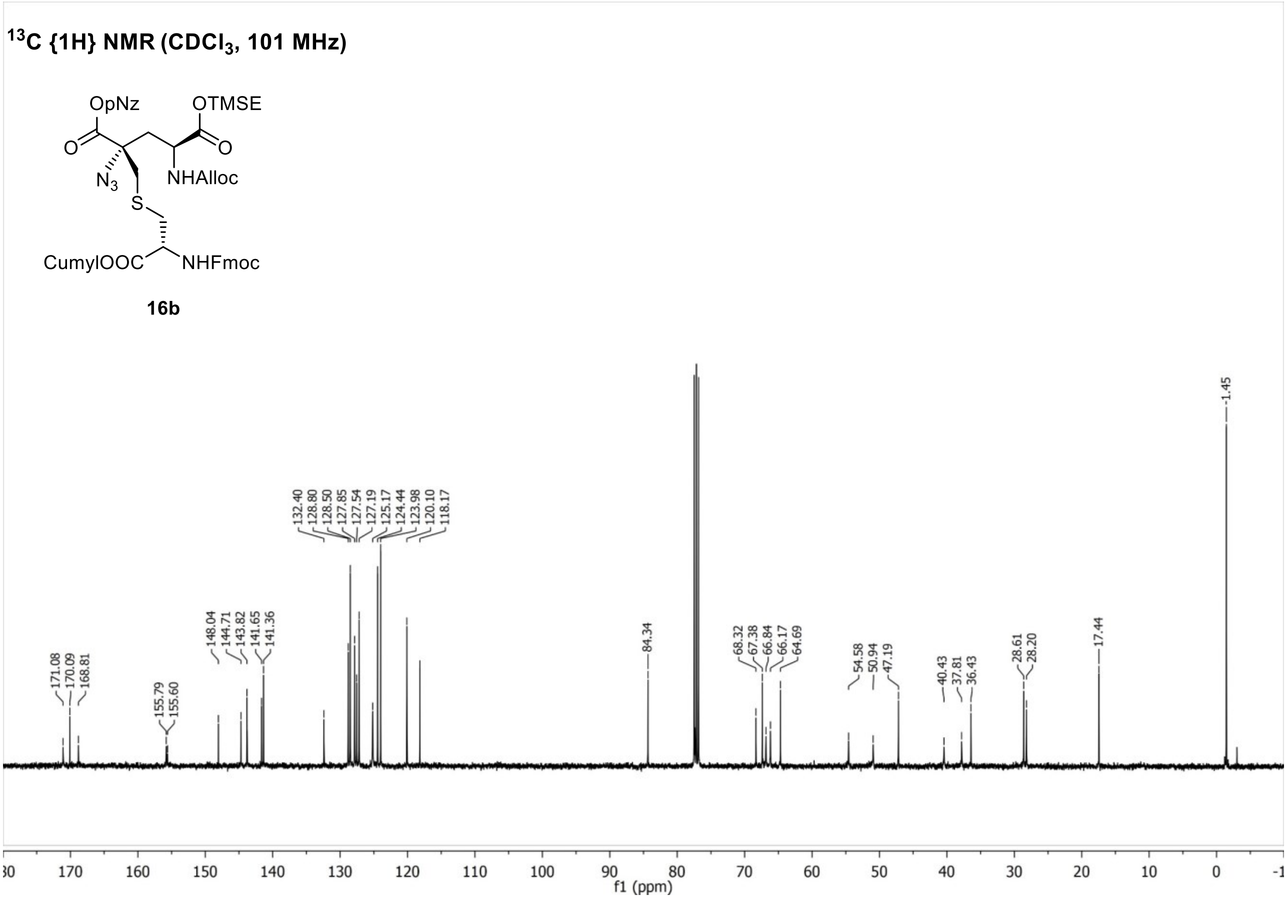
^{13}C {1H} NMR (CDCl$_3$, 101 MHz)
OpNz
OTMSE
N$_3$
NHAlloc
S
CumylOOC
NHFmoc
16b
f1 (ppm)

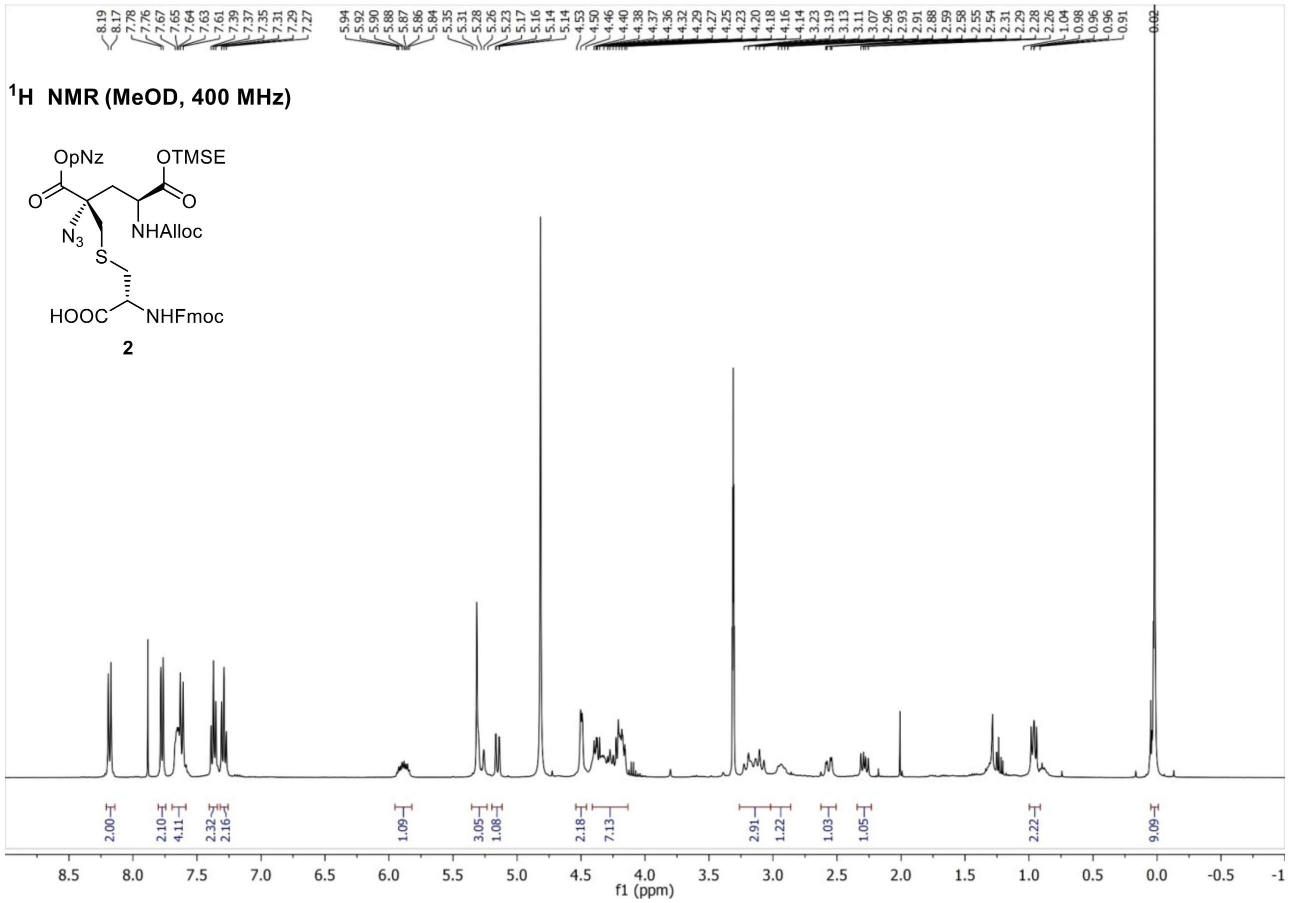

^{1}H NMR (MeOD, 400 MHz)
OpNz
OTMSE
O
O
N3
NHAlloc
S
HOOC
NHFmoc
2
f1 (ppm)

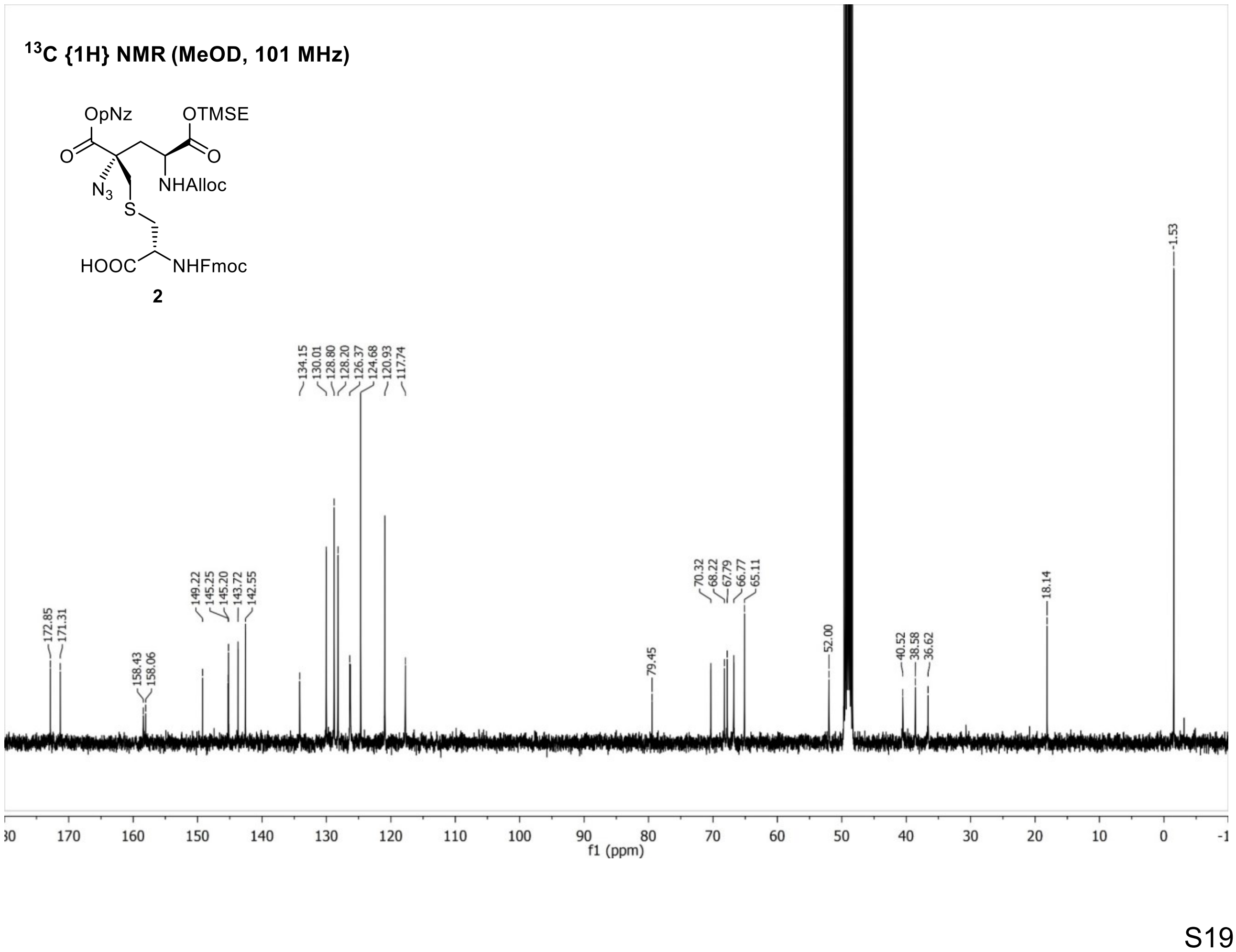

^{13}C {1H} NMR (MeOD, 101 MHz)
OpNz
OTMSE
O
O
N3
NHAlloc
S
HOOC
NHFmoc
2
f1 (ppm)

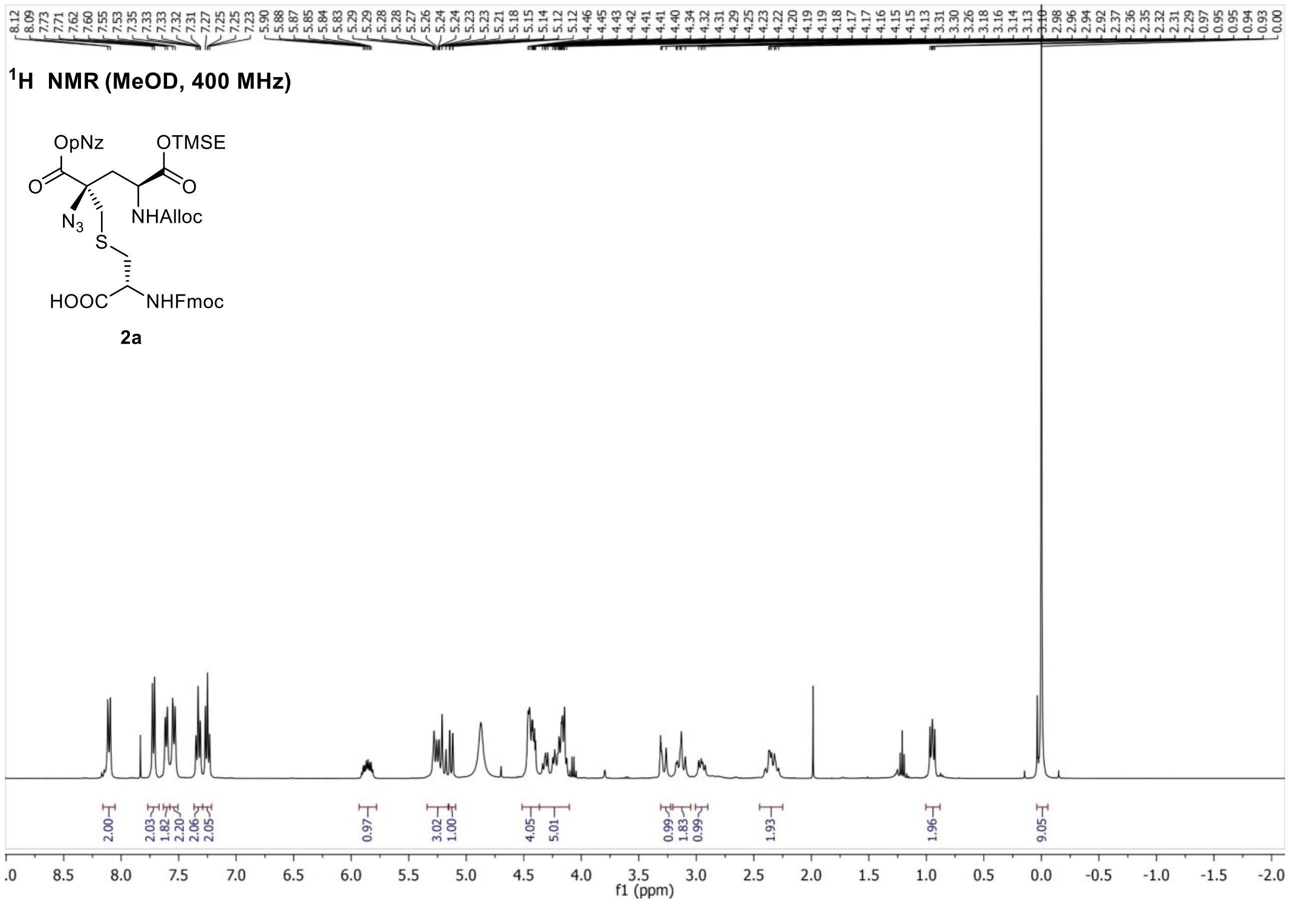

1H NMR (MeOD, 400 MHz)
OpNz
OTMSE
N3
NHAlloc
S
HOOC
NHFmoc
2a
f1 (ppm)

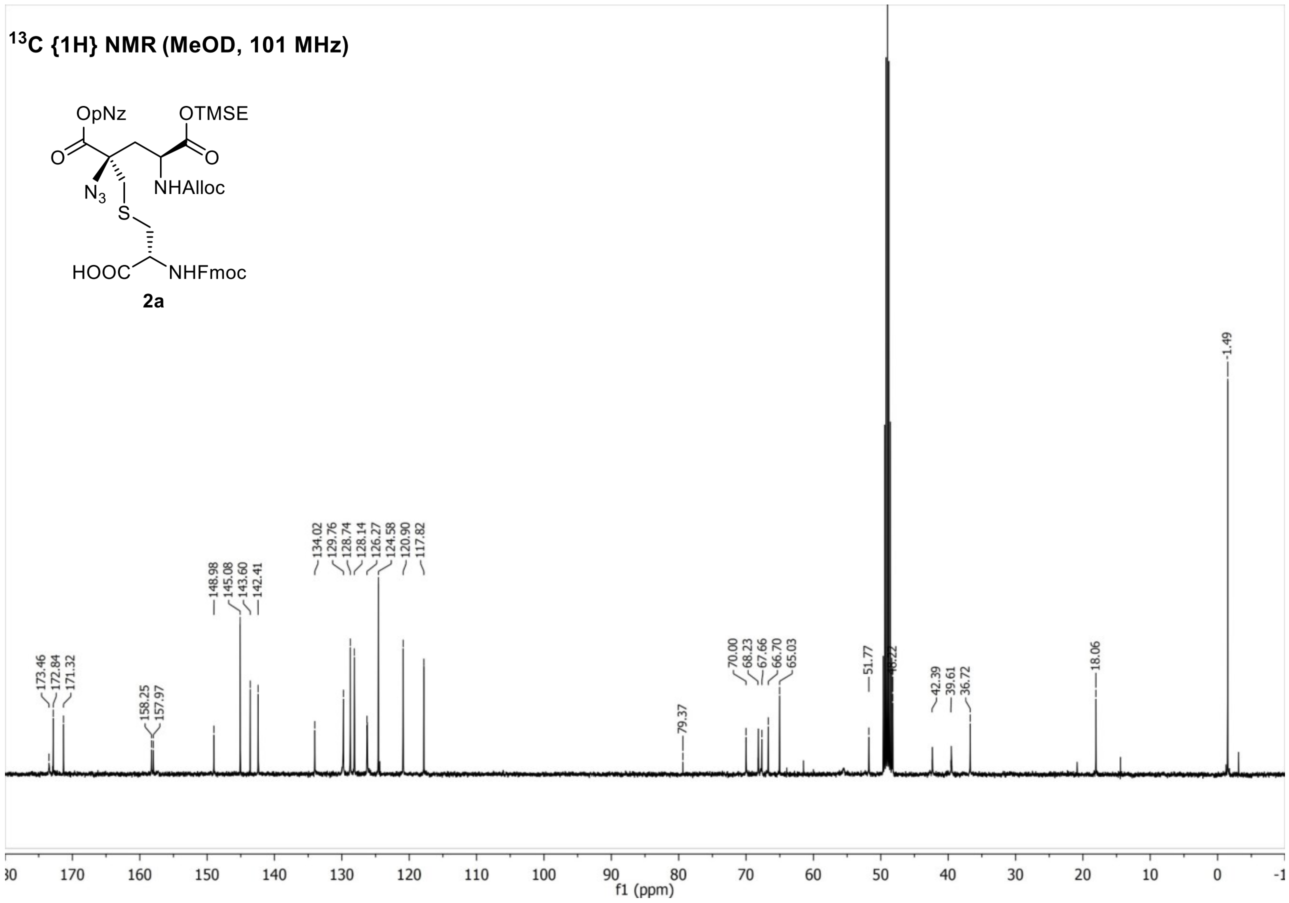

13C {1H} NMR (MeOD, 101 MHz)
OpNz
OTMSE
N3
NHAlloc
S
HOOC
NHFmoc
2a
173.46
172.84
171.32
158.25
157.97
148.98
145.08
143.60
142.41
134.02
129.76
128.74
128.14
126.27
124.58
120.90
117.82
79.37
70.00
68.23
67.66
66.70
65.03
51.77
42.39
39.61
36.72
18.06
-1.49
f1 (ppm)

Mass Spectra of compounds

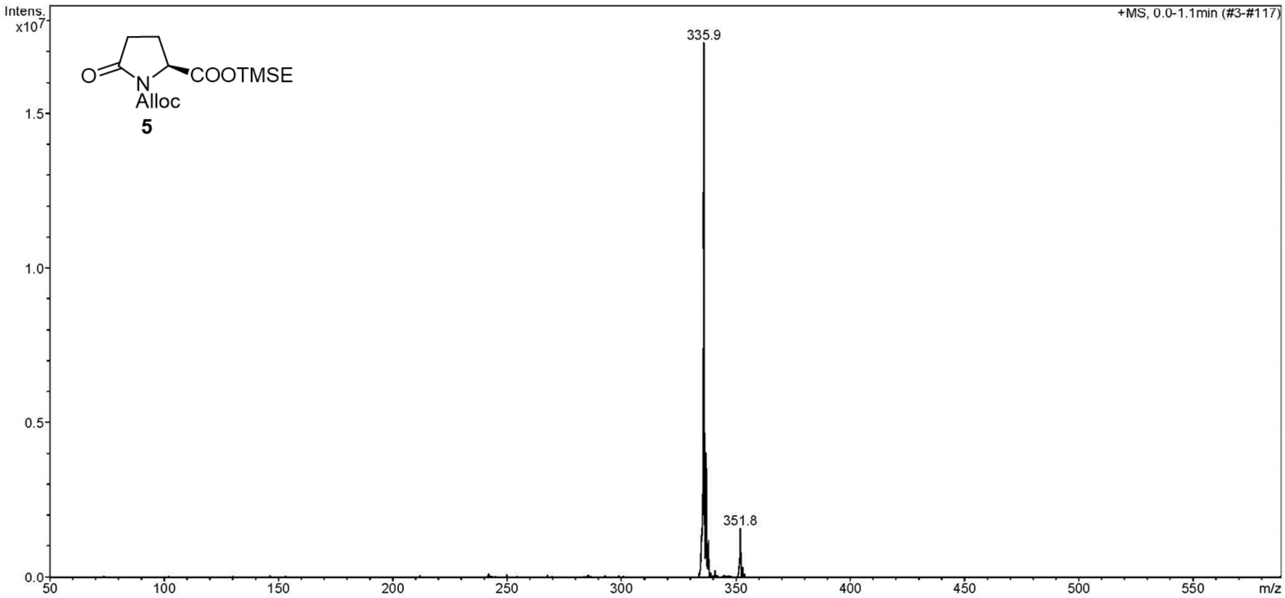

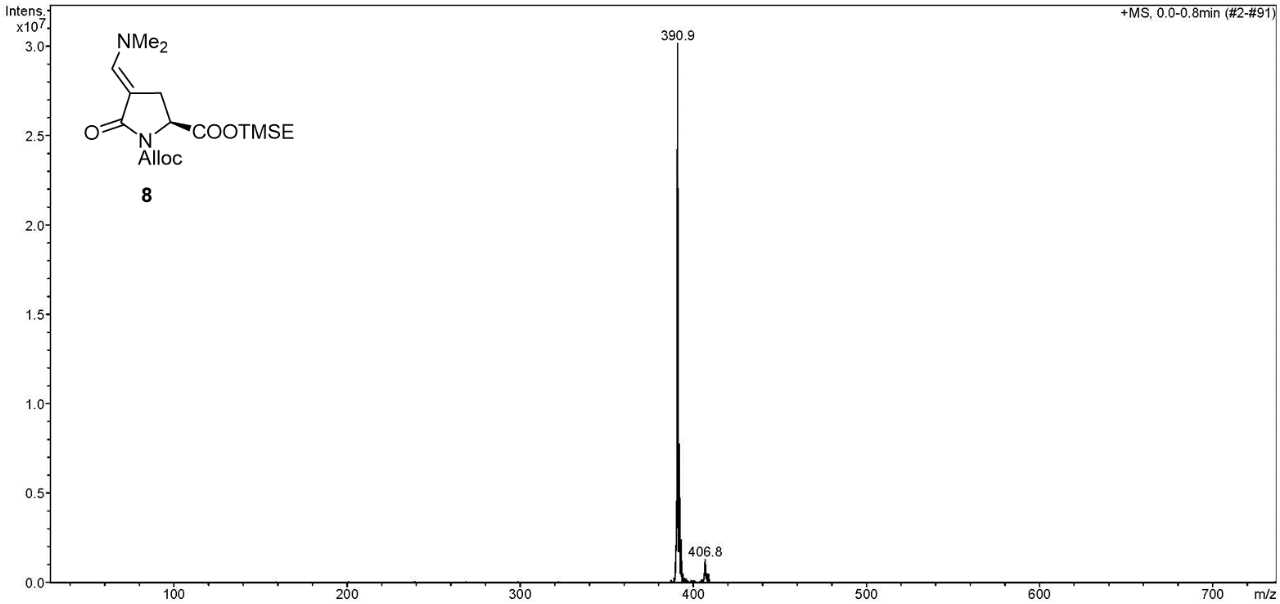

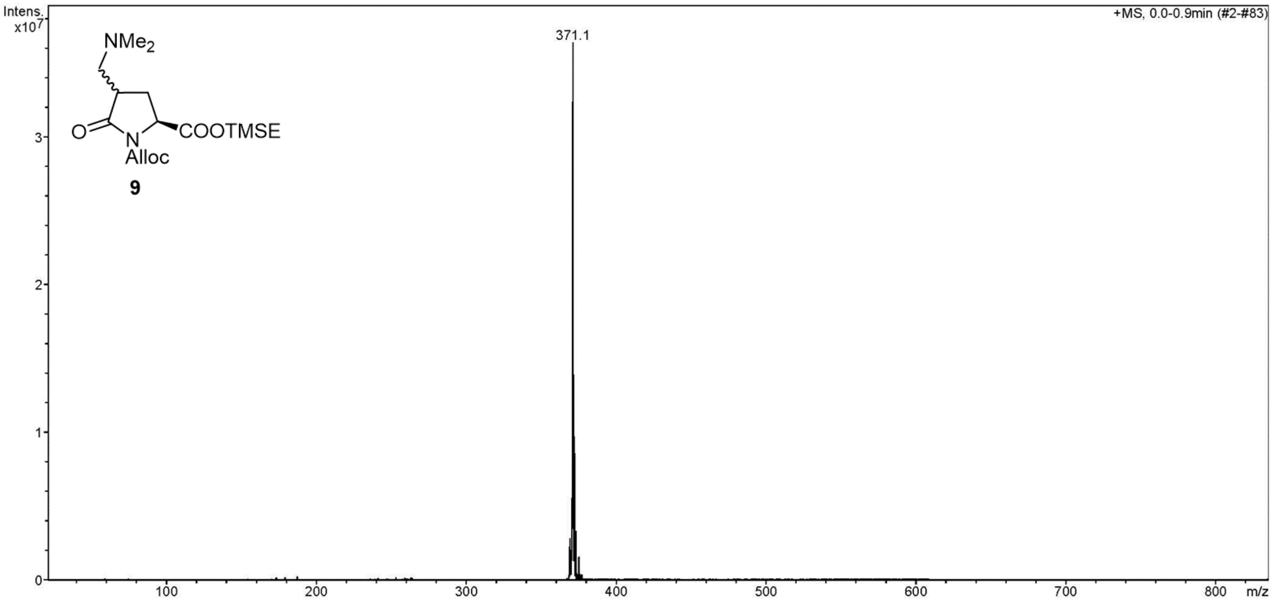

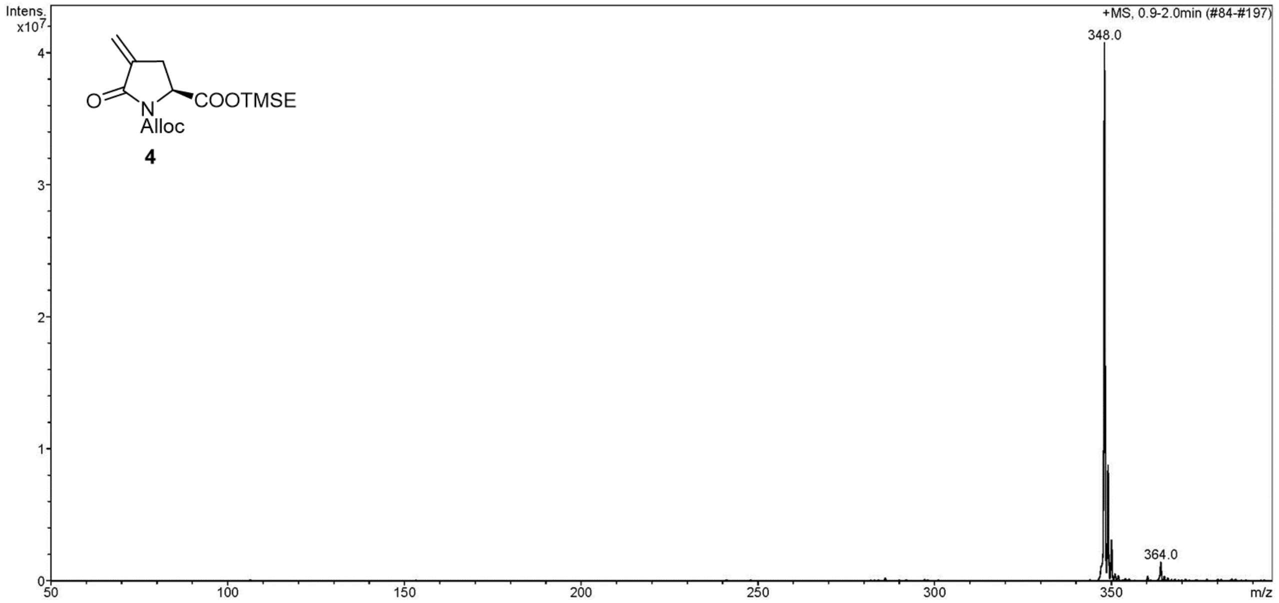
Intens.
x10^7
+MS, 0.9-2.0min (#84-#197)
348.0
364.0
O
N
Alloc
COOTMSE
4
m/z

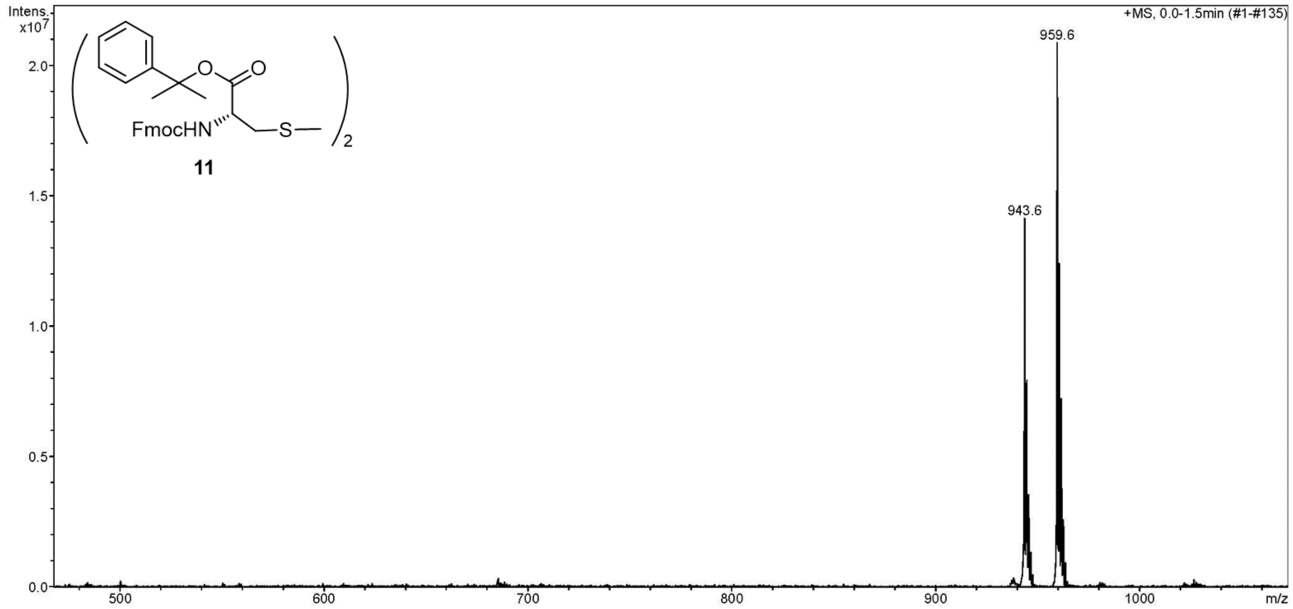
Intens.
x10^7
+MS, 0.0-1.5min (#1-#135)
959.6
943.6
O
O
FmocHN
S
2
11
m/z

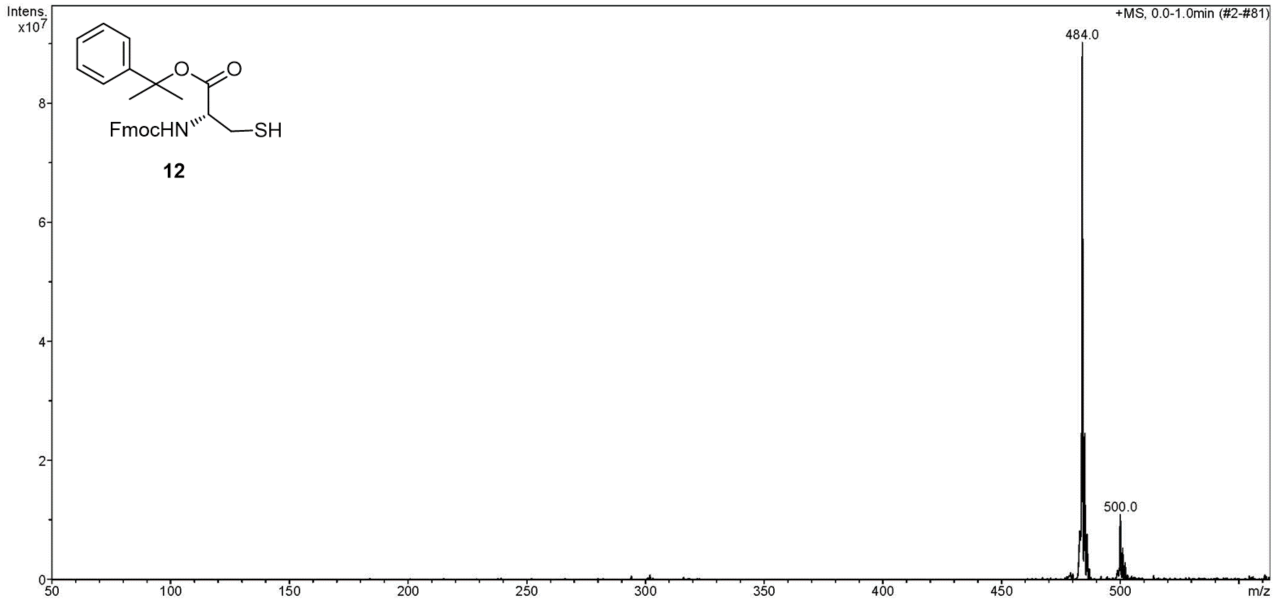
Intens.
x10^7
+MS, 0.0-1.0min (#2-#81)
484.0
500.0
O
O
FmocHN
SH
12
m/z

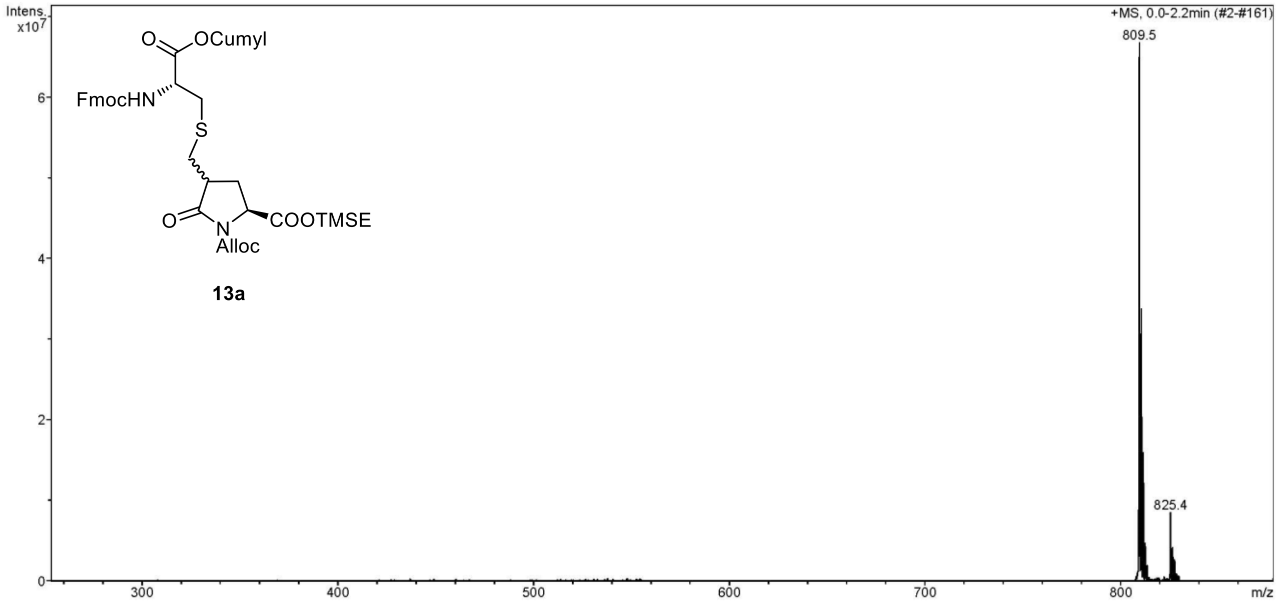
Intens.
x10^7
+MS, 0.0-2.2min (#2-#161)
809.5
825.4
m/z
O
OCumyl
FmocHN
S
O
N
COOTMSE
Alloc
13a

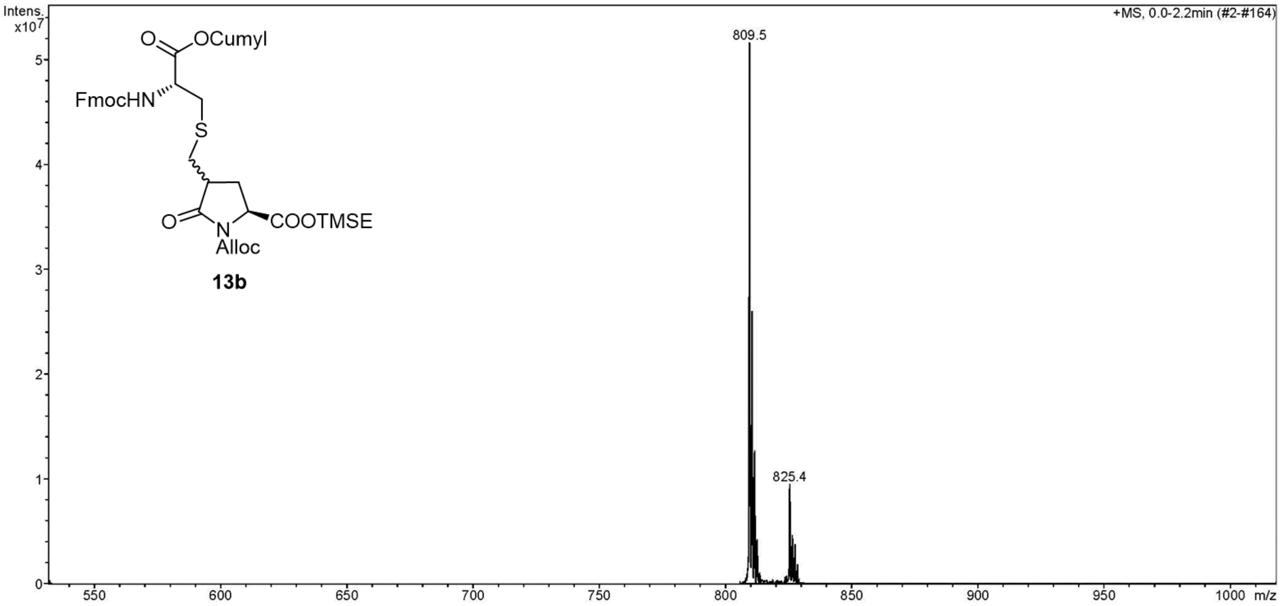
Intens.
x10^7
+MS, 0.0-2.2min (#2-#164)
809.5
825.4
m/z
O
OCumyl
FmocHN
S
O
N
COOTMSE
Alloc
13b

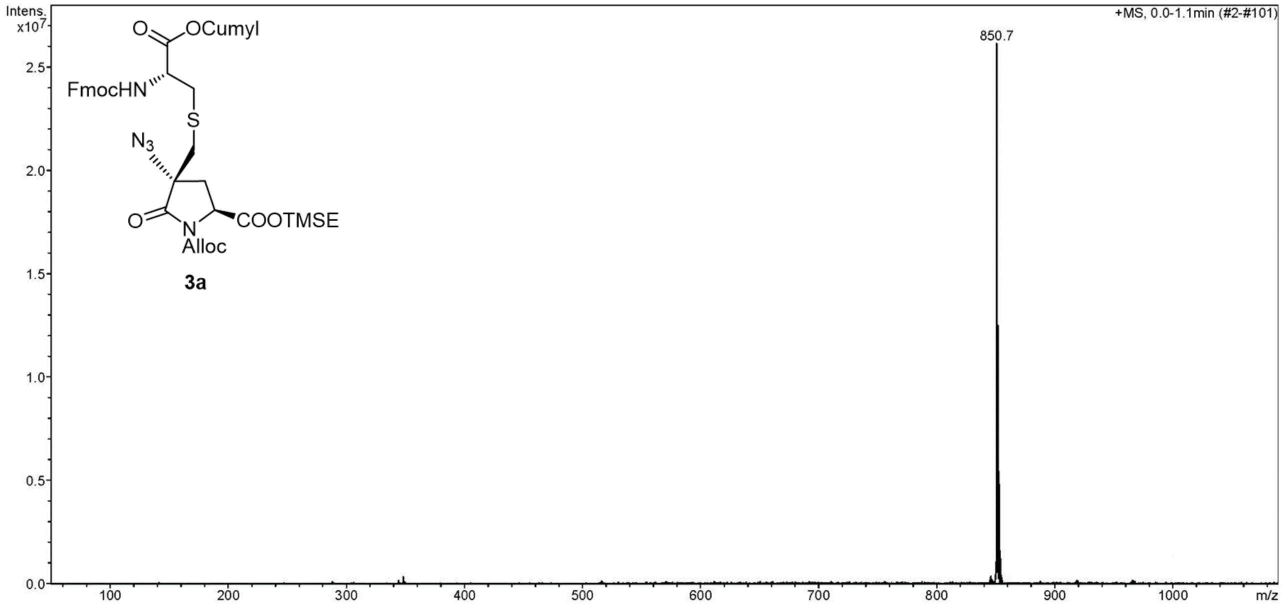
Intens.
x10^7
+MS, 0.0-1.1min (#2-#101)
850.7
m/z
O
OCumyl
FmocHN
S
N3
O
N
COOTMSE
Alloc
3a

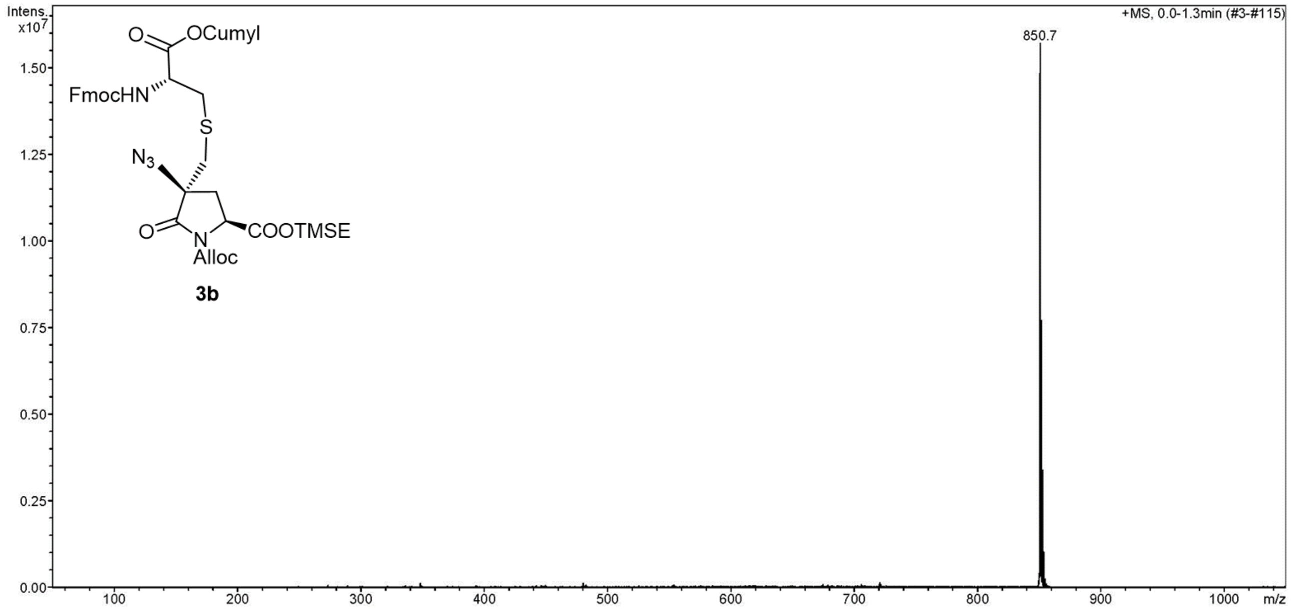
Intens. x10^7
+MS, 0.0-1.3min (#3-#115)
850.7
OCumyl
FmocHN
S
N3
COOTMSE
Alloc
3b
m/z

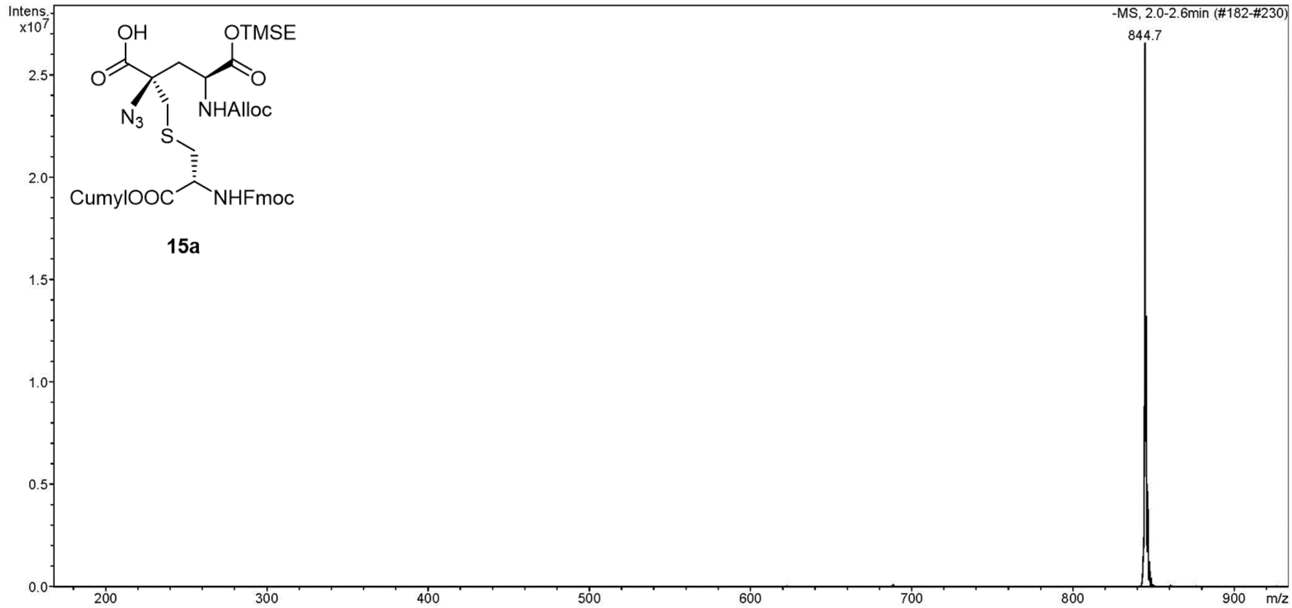
Intens. x10^7
-MS, 2.0-2.6min (#182-#230)
844.7
OH
OTMSE
N3
NHAlloc
S
CumylOOC
NHFmoc
15a
m/z

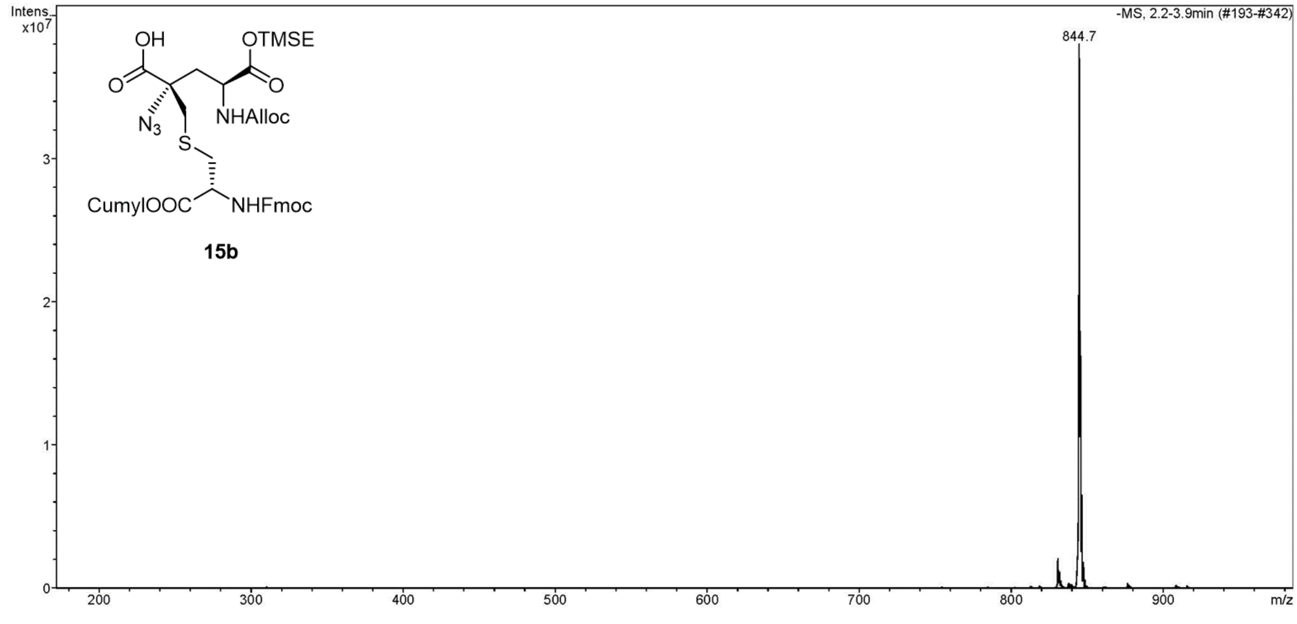
Intens. x10^7
-MS, 2.2-3.9min (#193-#342)
844.7
OH
OTMSE
N3
NHAlloc
S
CumylOOC
NHFmoc
15b
m/z

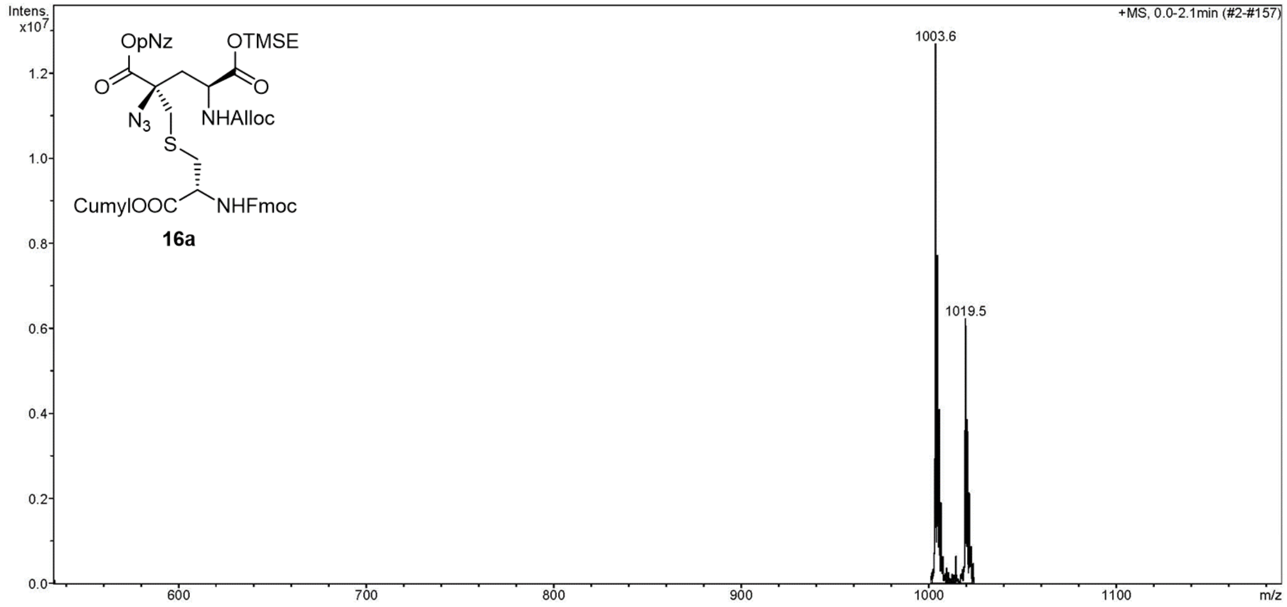
Intens.
x10^7
+MS, 0.0-2.1min (#2-#157)
1003.6
1019.5
OpNz
OTMSE
N3
NHAlloc
S
CumylOOC
NHFmoc
16a
m/z

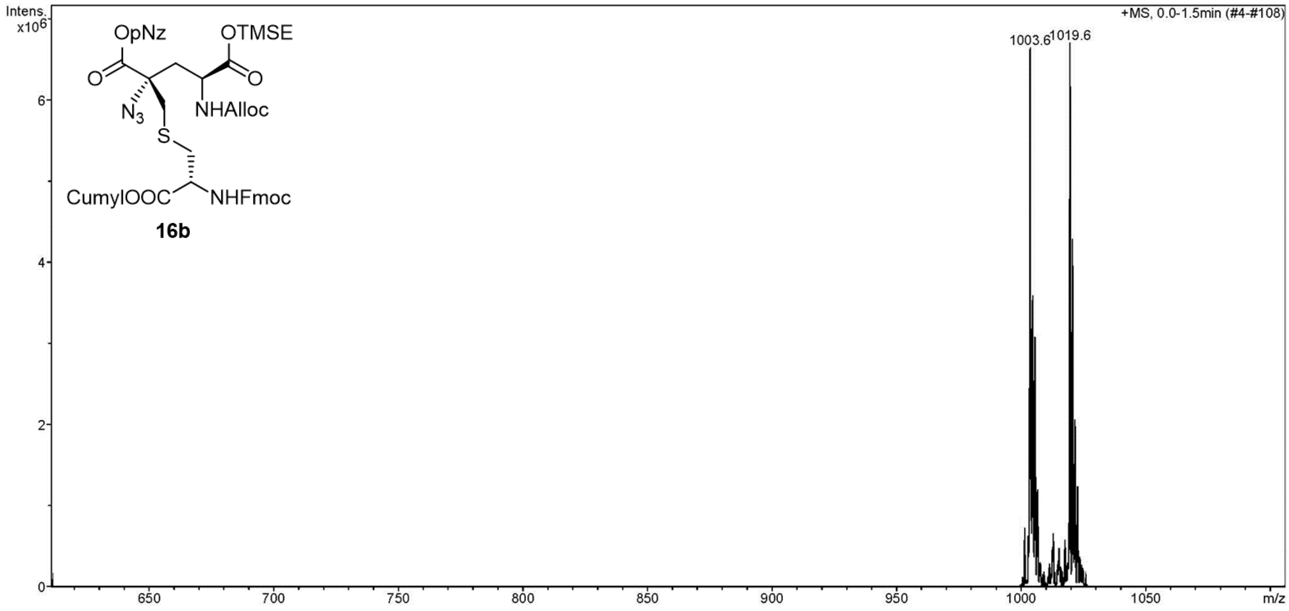
Intens.
x10^6
+MS, 0.0-1.5min (#4-#108)
1003.6
1019.6
OpNz
OTMSE
N3
NHAlloc
S
CumylOOC
NHFmoc
16b
m/z

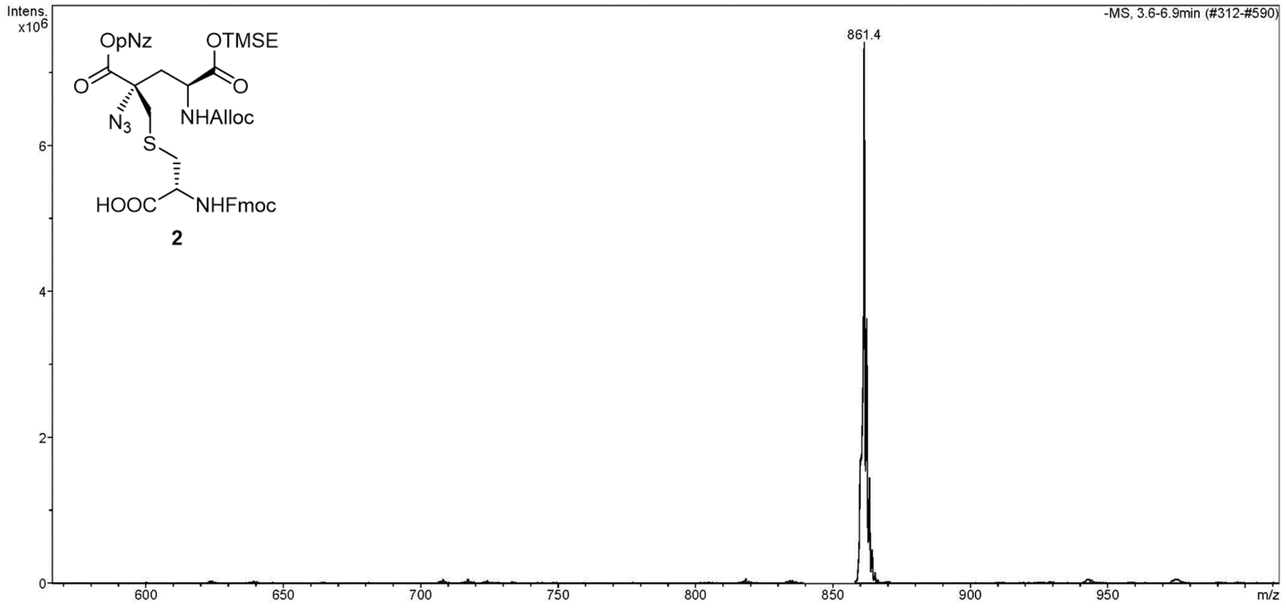
Intens.
x10^6
-MS, 3.6-6.9min (#312-#590)
861.4
OpNz
OTMSE
N3
NHAlloc
S
HOOC
NHFmoc
2
m/z

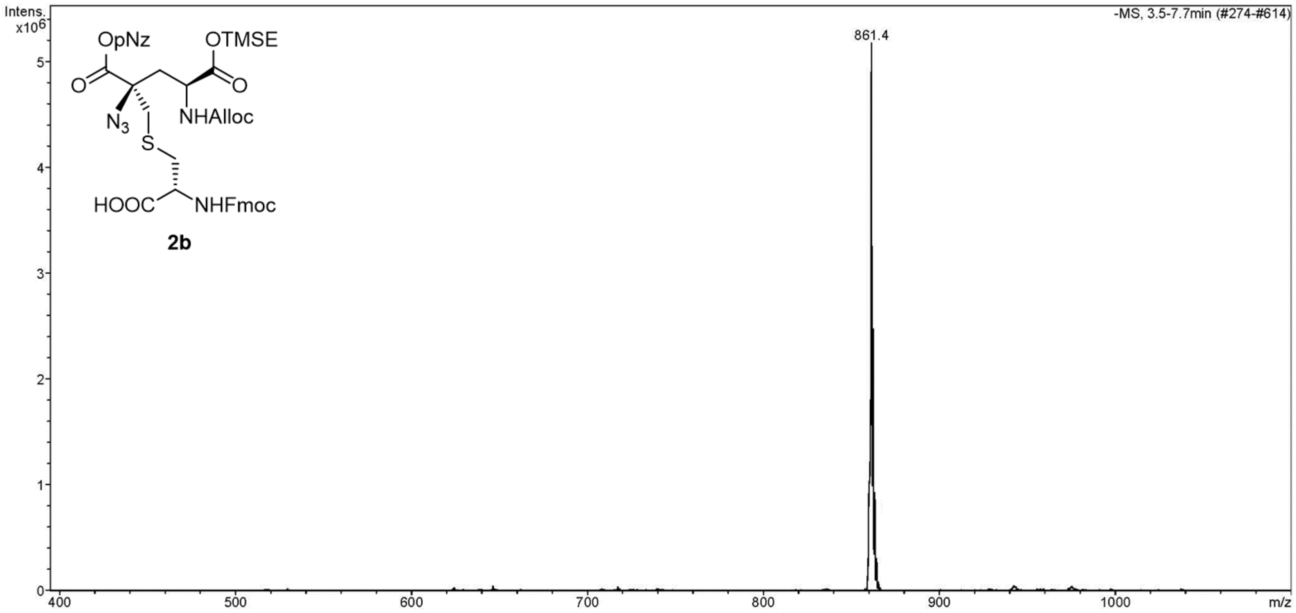
Intens.
x10^6
-MS, 3.5-7.7min (#274-#614)
861.4
OpNz
OTMSE
N3
NHAlloc
S
HOOC
NHFmoc
2b
m/z
